# Plant-based foods containing cell wall polysaccharides rich in specific active monosaccharides protect against myocardial injury in rat myocardial infarction models

**DOI:** 10.1038/srep38728

**Published:** 2016-12-08

**Authors:** Sun Ha Lim, Yaesil Kim, Ki Na Yun, Jin Young Kim, Jung-Hee Jang, Mee-Jung Han, Jongwon Lee

**Affiliations:** 1 Department of Biochemistry, School of Medicine, Catholic University of Daegu, Daegu 42472, Korea; 2 Hypoxi Co., LTD., Daegu 42472, Korea; 3 Biomedical Omics Group, Korea Basic Science Institute, Ochang 28119, Korea; 4 Sogang University, Seoul 04107, Korea; 5 Department of Pharmacology, School of Medicine, Keimyung University, Daegu 42601, Korea; 6 Department of Biomolecular and Chemical Engineering, Dongyang University, Yeongju, Gyeongbuk 36040, Korea

## Abstract

Many cohort studies have shown that consumption of diets containing a higher composition of foods derived from plants reduces mortality from coronary heart disease (CHD). Here, we examined the active components of a plant-based diet and the underlying mechanisms that reduce the risk of CHD using three rat models and a quantitative proteomics approach. In a short-term myocardial infarction (MI) model, intake of wheat extract (WE), the representative cardioprotectant identified by screening approximately 4,000 samples, reduced myocardial injury by inhibiting apoptosis, enhancing ATP production, and maintaining protein homeostasis. In long-term post-MI models, this myocardial protection resulted in ameliorating adverse left-ventricular remodelling, which is a predictor of heart failure. Among the wheat components, arabinose and xylose were identified as active components responsible for the observed efficacy of WE, which was administered via ingestion and tail-vein injections. Finally, the food components of plant-based diets that contained cell wall polysaccharides rich in arabinose, xylose, and possibly fucose were found to confer protection against myocardial injury. These results show for the first time that specific monosaccharides found in the cell wall polysaccharides in plant-based diets can act as active ingredients that reduce CHD by inhibiting postocclusion steps, including MI and heart failure.

Coronary heart disease (CHD) typically manifests as angina pectoris and myocardial infarction (MI) and has become the leading cause of death^1^. CHD is usually caused by atherosclerosis, and subsequent plaque buildup narrows the coronary arteries, leading to angina pectoris (preocclusion steps). Occasional plaque rupture and consequent thrombus formation result in artery occlusion (postocclusion steps). The resultant ischaemia is responsible for myocardial cell death via apoptosis and necrosis, and the extensive cell death leads to MI. Subsequently, post-MI events trigger adverse remodelling in the heart, leading to heart failure. Close to 70% of patients presenting with CHD die before receiving reperfusion therapy at a hospital^2^. Thus, the development of improved prevention strategies would play a crucial role in reducing CHD-related deaths.

To lower CHD risk, the consumption of a diet high in plant-derived foods has been recommended. Based on various cohort studies, the adherence to plant-based diets, such as Mediterranean and pescetarian diets^3^, and a higher consumption of their food components, such as whole grains, fruits, vegetables, pulses, nuts, and seafood^4^,^5^,^6^,^7^, are associated with a reduced risk of CHD mortality. It has been proposed that plant-based diets and their food components reduce the risk of CHD via preocclusion mechanisms that reduce the associated risk factors, resulting in improved lipid profiles and glycaemic control and reduced blood pressure^4^,^5^,^6^,^7^. Furthermore, no specific constituents have been demonstrated as active food components; rather, it appears that various constituents, including dietary fibre, polyphenols, phytosterols, unsaturated fatty acids, and vegetable proteins, act synergistically^4^,^5^. In addition, the majority of related studies have examined gene-diet interactions at the genomic and epigenomic levels, rather than the transcriptomic and proteomic levels, to identify molecular mechanisms^8^. Thus, the active components and their underlying mechanisms at the system level in plant-based diets that prevent CHD risk must be identified using various animal models in combination with omics tools.

Previously, we showed that the hot water extract of apple^9^ and apple pectin^10^, a cell wall polysaccharide, protects against ischaemia-reperfusion (IR) injury by inhibiting the apoptotic cascade in a rat MI model. Based on these results, we hypothesize that cell wall polysaccharides, which are the predominant constituents of dietary fibre in plant-based diets, contribute to the reduction of CHD risk by blocking postocclusion steps. To demonstrate this hypothesis, we used three rat IR models in combination with quantitative proteomics to investigate whether cell wall polysaccharides from various food components, which may also contain active monosaccharides, can indeed protect against myocardial injury.

## Results

### Short-term IR experiments indicate that wheat extract (WE) intake prevents myocardial injury by inhibiting apoptosis

In this study, cardioprotectants derived from plant extracts were identified by first screening approximately 4,000 samples supplied by the Korea Plant Extract Bank (Korea Research Institute of Bioscience & Biotechnology, Daejeon, Korea) using an *in vitro* cell culture system that mimics *in vivo* ischaemia. We selected WE as a representative cereal grain of plant-based diets from the candidates^11^,^12^, as it improved cell survival under ischaemic conditions by inhibiting apoptosis (Supplementary Fig. S1).

**Figure 1:**
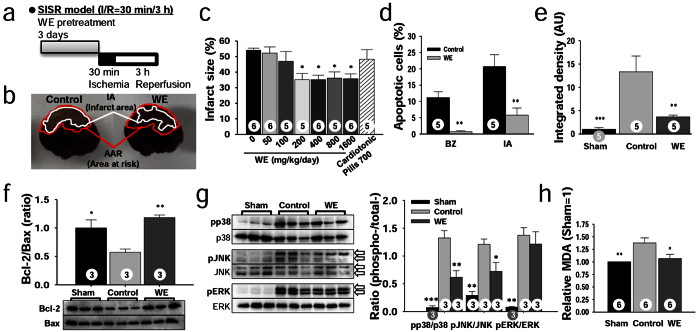
WE protects against myocardial injury from SISR by inhibiting apoptosis. (**a**) Design of the SISR experiments. (**b**) Representative heart sections demarcating the area at risk (AAR) and infarct area (IA) for the control and WE-treated groups, respectively. (**c**) Effect of WE intake on infarct size (IS). (**d**) Ratio of apoptotic cells to total cells in the border zone (BZ) and IA as assessed by TUNEL and methyl green staining. (**e**) Relative levels of cleaved caspase-3 in the AAR assessed by immunohistochemical staining. AU, arbitrary unit. (**f**) Bcl-2 and Bax levels presented as the Bcl-2/Bax ratio in the AAR, measured by Western blotting. (**g**) Phosphorylated p38, JNK, and ERK levels in the AAR as measured by Western blotting (left) and pMAPK/total MAPK ratios (right). The original bands are presented in Supplementary Fig. S2. (**h**) Relative malondialdehyde (MDA) levels in the AAR analysed through a chromogenic assay. In panels (**d–h**), WE was supplemented at a dose of 400 mg/kg/day. *P < 0.05, **P < 0.01, ***P < 0.001 compared with the controls. The numbers inside the bars indicate the number of animals per group.

To determine whether WE could protect against myocardial injury *in vivo* during the postocclusion steps of CHD, rats were supplemented with various doses of WE (0–1,600 mg/kg/day) for 3 days, and the rats then underwent short-term ischaemia (30 minutes) followed by short-term reperfusion (3 hours) (SISR; Fig. 1a–c). WE supplementation over 200 mg/kg/day significantly reduced the infarct size (IS) compared with the control group (35.1 ± 4.1% vs. 54.1 ± 1.4%) (Fig. 1c). A comparison was performed by employing Simjukhwan, referred to as a Compound Danshen dripping pill (CDDP), an herbal medicine developed in China to treat angina pectoris^13^. CDDP supplementation at 700 mg/kg/day, which is equivalent to 10 times the dose calculated using a conversion factor provided by the Food and Drug Administration^14^, was not effective (P > 0.05) (Fig. 1c). These results show that WE acts through different underlying mechanisms than CDDP to protect against CHD due to different target diseases.

We next examined whether WE intake could inhibit apoptotic cascades *in vivo*. The ratios of apoptotic cells [terminal deoxynucleotidyl transferase dUTP nick-end labelling (TUNEL)-positive cells] to total cells in both the border zone (BZ) and the infarct area (IA) were significantly attenuated in the WE-treated group (400 mg/kg/day) compared with the control group (0.69 ± 0.3% vs. 11.2 ± 1.8% in the BZ; 5.8 ± 2.2% vs. 20.7 ± 3.7% in the IA) (Fig. 1d), indicating that WE intake inhibits apoptosis *in vivo*. In addition, the relative colour intensity, indicating the level of the cleaved caspase-3 derived from procaspase-3, was significantly attenuated in the WE-treated group compared with the control group (3.7 ± 0.4 vs. 13.3 ± 3.4) (Fig. 1e). These findings show that WE intake inhibits the activation of procaspase-3 to caspase-3. Furthermore, the Bcl-2/Bax ratio was significantly increased in the WE-treated group compared with the control group (1.2 ± 0.04 vs. 0.6 ± 0.05) (Fig. 1f, Supplementary Fig. S2), suggesting that WE intake modulates Bcl-2 and Bax expression and inhibits apoptosis. With regard to MAPK levels, the pp38/p38 and pJNK/JNK ratios were significantly attenuated in the WE-treated group compared with the control group (0.62 ± 0.12 vs. 1.3 ± 0.13 for pp38/p38; 0.72 ± 0.16 vs. 1.2 ± 0.10 for pJNK/JNK) (Fig. 1g, Supplementary Fig. S2), indicating that WE intake inhibits p38 and JNK phosphorylation. Then, we assessed the reactive oxygen species (ROS) levels in the heart as an upstream element of MAPK, for which we measured malondialdehyde (MDA) levels^15^. The relative MDA levels in the area at risk (AAR) were significantly reduced in the WE-treated group compared with the control group (1.07 ± 0.1 vs. 1.38 ± 0.1) (Fig. 1h). These findings show that WE acts as an antioxidant by removing ROS formed during IR. Taken together, the results from the rat SISR model indicate that WE intake protects against myocardial injury during the postocclusion steps by inhibiting apoptosis through decreased ROS generation, decreased MAPK activation, an increased Bcl-2/Bax ratio, decreased caspase-3 production, and decreased DNA-nick generation.

### Quantitative proteomics and immunoblotting reveal that WE intake mainly affects apoptosis, energy production, and protein homeostasis

**Figure 2:**
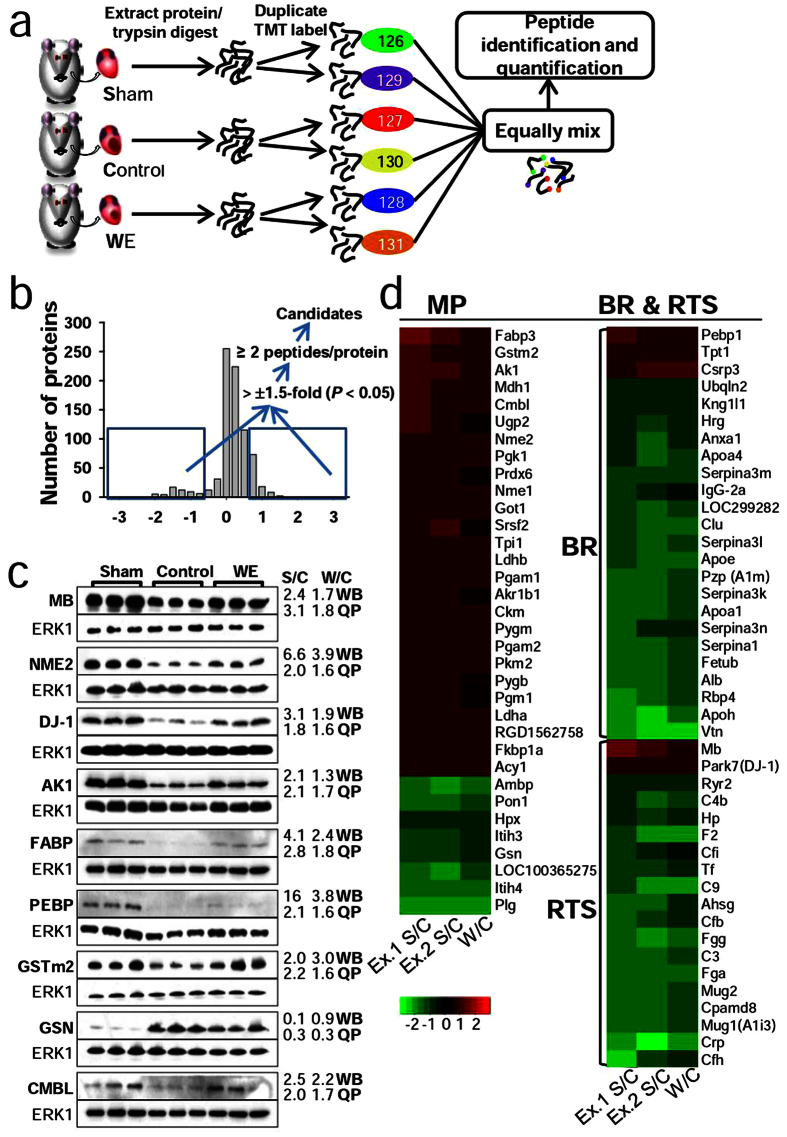
Quantitative proteomics analysis for the identification of proteins modulated by WE intake. (**a**) Flow chart of the experimental scheme. In each experiment, peptide digests from the sham, control, and WE-treated group were individually labelled with two different TMT-sixplex tags to exclude technical variations. The labelled peptides were equally mixed and subjected to identification and quantification via tandem MS. (**b**) The scheme for candidate identification and log_2_ ratio plot for heart proteins obtained from the sham and control. (**c**) Representative Western blot results of 9 candidates (MB, NME2, DJ-1, FABP, AK-1, RKIP, GSTm2, CMBL, and GSN) and a loading control (ERK1). Immunoblotting was performed using the protein extracts from batches of rat heart tissues, which were different from those used for the TMT labelling experiments. Average fold changes of the quantitative Western blot (WB) results from biological triplicates and quantitative proteomics (QP). The original bands are presented in Supplementary Fig. S3. MB, myoglobin; NME2, nucleoside diphosphate kinase; FABP, fatty acid-binding protein; AK-1, adenylate kinase; RKIP, phosphatidylethanolamine-binding protein; GSTm2, glutathione S-transferase; CMBL, carboxymethylenebutenolidase; GSN, gelsolin. (**d**) Log_2_ heat map for overlapping candidates between the sham/control and WE/control data sets.

To identify additional key proteins modulated by WE intake in the SISR model and the underlying mechanisms at the system level, we applied isobaric tandem mass tag (TMT)-based quantitative mass spectrometry (MS) (Fig. 2a). The protein expression levels in the sham and WE-treated groups were quantified relative to those of the control group to identify proteins influenced by WE intake. Proteins were selected according to a fold-change threshold of >±1.5 (*P* < 0.05) and the presence of two or more peptides, and they were subsequently filtered against the relative abundance of the proteome, which was independently measured by liquid chromatography (LC)-MS/MS (Fig. 2b). We identified 207 proteins that were significantly differentially expressed between the sham and control groups, and 80 were found to be common in two independent biological experiments (Table S1). We also identified 112 significantly altered proteins between the WE-treated and control groups, and 74 exhibited alterations in the same direction in both the WE/control and sham/control groups (Table S2). This approach allowed for the robust identification and quantification of the proteins intimately associated with MI. For example, the results are consistent with those of previous reports for fatty acid-binding protein (FABP)^16^, glutathione S-transferase (GSTm2)^17^, and gelsolin (GSN)^18^. Thus, the proteins filtered by our criteria appear to have the potential to facilitate an understanding of the underlying mechanisms of key proteins involved in protein modulation by WE intake in the SISR model. To the best of our knowledge, this is the largest dataset generated for rat cardiac injury to date.

To verify the protein quantification determined by the proteome analysis with Western blotting, nine candidates (MB, NME2, DJ-1, FABP, AK-1, RKIP, GSTm2, CMBL, and GSN) were selected according to their importance based on a literature search and commercial antibody availability. The Western blotting results were consistent with the results of the proteome analysis, although the changes in magnitude were slightly different (Fig. 2c, Supplementary Figs S3 and 4).

**Figure 3:**
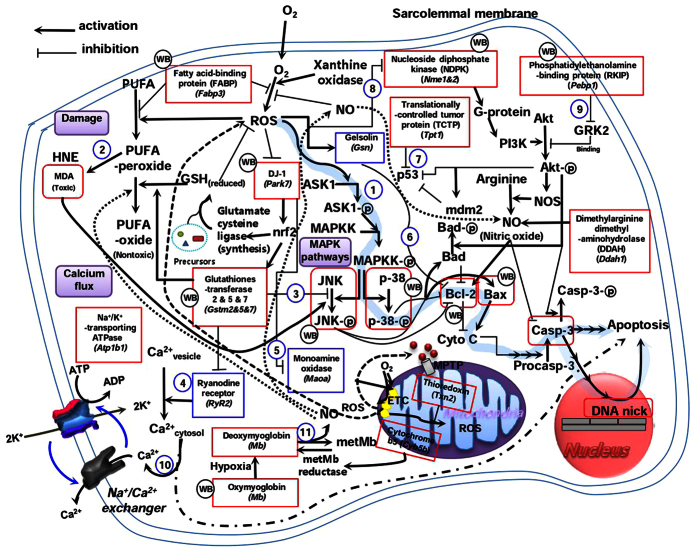
Apoptotic cascade (pathway 1) modulated by WE treatment. The results indicate that reduced MDA generation inhibits JNK phosphorylation (pathway 2); increased DJ-1 expression promotes the expression of glutathione S-transferase (Gstm2), which inhibits JNK phosphorylation (pathway 3) and ryanodine receptor (RyR2) activity; and inhibited RyR2 activity subsequently reduces the cytosolic calcium concentration (pathway 4), which is also influenced by the increased expression of Na^+^/K^+^-transporting ATPase (Atp1b1) (pathway 10). However, increased DJ-1 expression inhibits the activity of monoamine oxidase (Maoa) (pathway 5); decreased gelsolin (Gsn) expression promotes Bcl-2 expression (pathway 6); increased translationally controlled tumour protein (Tpt1) expression promotes p53 degradation (pathway 7); and increased nucleoside diphosphate kinase (Nme) (pathway 8) and phosphatidylethanolamine-binding protein (Pebp1) (pathway 9) expression promote Akt phosphorylation. Proteins in the rectangles were confirmed in this study. WB indicates the proteins that were further confirmed by Western blot analysis. Proteins coloured red and blue are up-regulated and down-regulated, respectively. The unboxed components were arranged according to published sources. GSH, glutathione; ETC, electron transport chain; GRK2, G-protein coupled receptor kinase; HNE, 4-hydroxynonenal; metMb, metmyoglobin; MPTP, mitochondrial permeability transition pore; NOS, nitric oxide synthase; PI3K, phosphoinositide 3-kinase; PUFA, polyunsaturated fatty acids.

Furthermore, the proteins that were differentially regulated by WE intake were analysed to identify relevant functional pathways using DAVID bioinformatics resources. The top three ranking biological processes were response to stimulus (RTS), metabolic process (MP), and biological regulation (BR) (Fig. 2d and Table S3). The majority of proteins involved in the MP category were increased, whereas those in the BR and RTS categories were decreased. The alterations in gene expression for the BR and RTS categories can be linked to the apoptotic cascade (Fig. 3). Overall, gene expression is modulated to reduce ROS production as well as the resultant deleterious events. These modulations eventually inhibit the apoptotic cascade. In the MP category (Supplementary Fig. S5), WE intake enhances carbohydrate and fat metabolism aided by increased oxygen capacity to generate more ATP through respiration. The intake also increases ATP levels by converting ADP, GTP, and phosphocreatine into ATP. In addition, the intake simultaneously enhances protein synthesis and degradation as well as cytoskeleton assembly and disassembly. Collectively, these findings suggest that the beneficial effects of WE stem from its ability to inhibit apoptosis and maintain cellular ATP levels and protein homeostasis.

### WE intake ameliorates adverse left ventricular (LV) remodelling in long-term IR experiments

**Figure 4:**
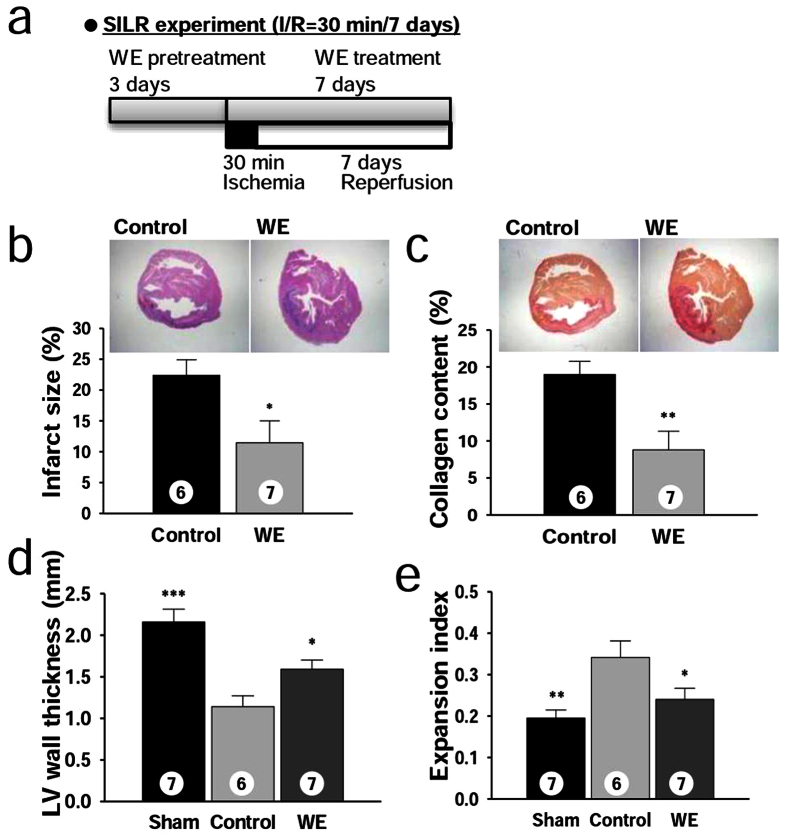
WE mitigates adverse LV remodelling after MI in the SILR model. (**a**) Design of the SILR experiments with a WE dose of 400 mg/kg/day. (**b**) Representative sections stained with haematoxylin-eosin staining (top), and infarct size (bottom). (**c**) Representative sections stained with Sirius red (top), and collagen content presented as the stained area/left ventricular (LV) area. (**d**) Average LV wall thickness. (**e**) Expansion index. *P < 0.05, **P < 0.01, ***P < 0.001 compared with the controls. The numbers inside bars indicate the number of animals per group.

As several genes (MB, DJ-1, and GSN) identified via quantitative proteomics are also involved in heart failure following MI^18^,^19^,^20^, we examined whether WE intake could modulate pathological LV remodelling after MI. In the first experiment, WE (400 mg/kg/day) was supplemented for 3 days prior to short-term ischaemia (30 minutes) followed by long-term reperfusion (7 days) (SILR; Fig. 4a), and supplementation was then maintained throughout the reperfusion period. The IS was significantly attenuated in the WE-treated group compared with the control group (11.4 ± 3.6% vs. 22.4 ± 2.5%) (Fig. 4b). These findings indicate that the protective effects of WE intake are sustained even during the long-term periods (Fig. 1c) of reperfusion. The collagen content was significantly reduced in the WE-treated group compared with the control group (8.8 ± 2.5% vs. 19 ± 1.8%) (Fig. 4c), suggesting that less space is available for fibroblasts to express collagen in the WE-treated hearts because of cardiomyocyte survival in the lesion. Furthermore, the LV wall thickness was significantly increased in the WE-treated group compared with the control group (1.6 ± 0.11 vs. 1.1 ± 0.13) (Fig. 4d). In addition, we investigated whether a reduction of the IA and preservation of the LV wall thickness prevented LV expansion by calculating the expansion index (Eq. 1)^21^. The expansion index was significantly reduced in the WE-treated group compared with the control group (0.24 ± 0.03 vs 0.34 ± 0.04), indicating that WE intake preserves LV morphology (Fig. 4e).

**Figure 5:**
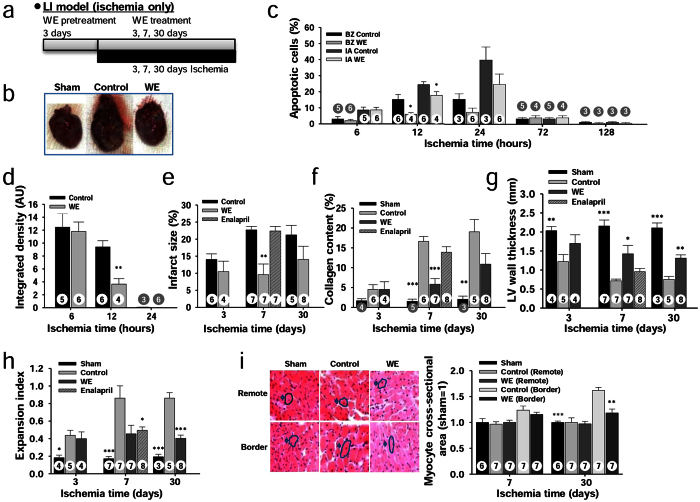
WE mitigates adverse LV remodelling after MI in the LI model. (**a**) Design of long-term ischaemia-only (LI) experiments with a WE dose of 400 mg/kg/day. (**b**) Representative heart morphologies after 30 days of LI. (**c**) Ratio of apoptotic cells to total cells in the infarct area. (**d**) Relative levels of cleaved caspase-3. (**e**) Infarct size assessed through H&E staining. (**f**) Collagen content. (**g**) Average LV wall thickness. (**h**) Expansion index. (**i**) Representative sections showing demarcated cardiomyocytes (left) and cardiomyocyte cross-sectional area (right). Effect of 10 mg/kg/day enalapril supplementation at 7 days is shown as a striped line on bar graphs for comparative purposes (**e**–**h**) (see Supplementary Fig. S6 for more accurate data). *P < 0.05, **P < 0.01, ***P < 0.001 compared with the controls. The numbers inside bars indicate the number of animals per group.

In the second experiment, the LAD coronary artery was ligated during a long-term period of ischaemia (3, 7 or 30 days) without subsequent reperfusion (LI model; Fig. 5a). After 30 days of occlusion, WE intake prevented LV expansion, which resulted in heart morphology that was similar to that observed for the sham group (Fig. 5b). WE supplementation significantly reduced the number of apoptotic cells in both the BZ and IA after 12 hours of occlusion compared with the control group (6.0 ± 1.0% vs. 15.2 ± 3.1% in the BZ; 17.8 ± 2.3% vs. 24.4 ± 1.8% in the IA) (Fig. 5c). Cleaved caspase-3 levels were also significantly reduced at 12 hours of occlusion in the WE-treated group compared with the control group (3.65 ± 0.8 vs. 9.40 ± 0.9) (Fig. 5d). These findings show that WE intake inhibits the apoptotic cascade over a short-term period of permanent occlusion (<1 day). Furthermore, the IS was significantly reduced in the WE-treated group compared with the control group at 7 days of occlusion (9.60 ± 3.1 vs. 22.7 ± 1.0%) (Fig. 5e). This myocardial protection resulted in decreased collagen deposition in the IA in the WE-treated group compared with the control group again at 7 days of occlusion (5.82 ± 1.4% vs. 16.6 ± 1.2%) (Fig. 5f). Myocardial protection attributable to WE intake also resulted in increased LV wall thickness (Fig. 5g) and a reduced expansion index (Fig. 5h) in the WE-treated group compared with the control group (1.43 ± 0.22 vs. 0.72 ± 0.04 and 0.45 ± 0.10 vs. 0.86 ± 0.14 at 7 hours, respectively). These findings indicate that WE intake preserves LV morphology, which is attributable to less damage in the IA. Finally, the cardiomyocyte size was significantly reduced in the BZ in the WE-treated group compared with the control group at 30 days of occlusion (1.18 ± 0.07 vs. 1.62 ± 0.06) (Fig. 5i). These results indicate that myocardial protection attributable to WE intake incurs less wall stress, which leads to preservation of the original cardiomyocyte size.

As a positive control, we further tested enalapril, an angiotensin-converting enzyme inhibitor that decreases LV dilation and reduces mortality caused by heart failure. Supplementation with enalapril at 10 mg/kg/day, a dose that improves LV function in myocardial-infarcted rats^22^, significantly increased the LV wall thickness (0.96 ± 0.08 vs. 0.72 ± 0.04) and decreased the expansion index (0.50 ± 0.04 vs. 0.86 ± 0.14) compared with the control group at 7 days in the LI model (Fig. 5e–h, Supplementary Fig. S6). However, enalapril supplementation did not significantly decrease the IS (*P* > 0.05) or collagen content (*P* > 0.05). These findings indicate that WE supplementation provides more effective amelioration of adverse LV remodelling than enalapril under our experimental conditions. Taken together, WE intake mitigated adverse LV remodelling through reduced IS, increased LV wall thickness, reduced expansion index, reduced collagen content, and reduced myocyte hypertrophy.

### Arabinose and xylose are key wheat components that protect against myocardial injury

To identify the components of WE responsible for the observed efficacy of WE treatment, we tested various commercially available ingredients that constitute the wheat grain. The structures and detailed descriptions of the polysaccharides examined in this study are presented in Table S4. The wheat grain is primarily composed of starch and protein as nutrients and cell wall polysaccharides as a dietary fibre. Specifically, the cell wall polysaccharides include arabinoxylan, cellulose, β-glucan, and arabinogalactan-peptide^23^,^24^. Phenolic acids, including ferulic acid, are minor components^24^.

**Figure 6:**
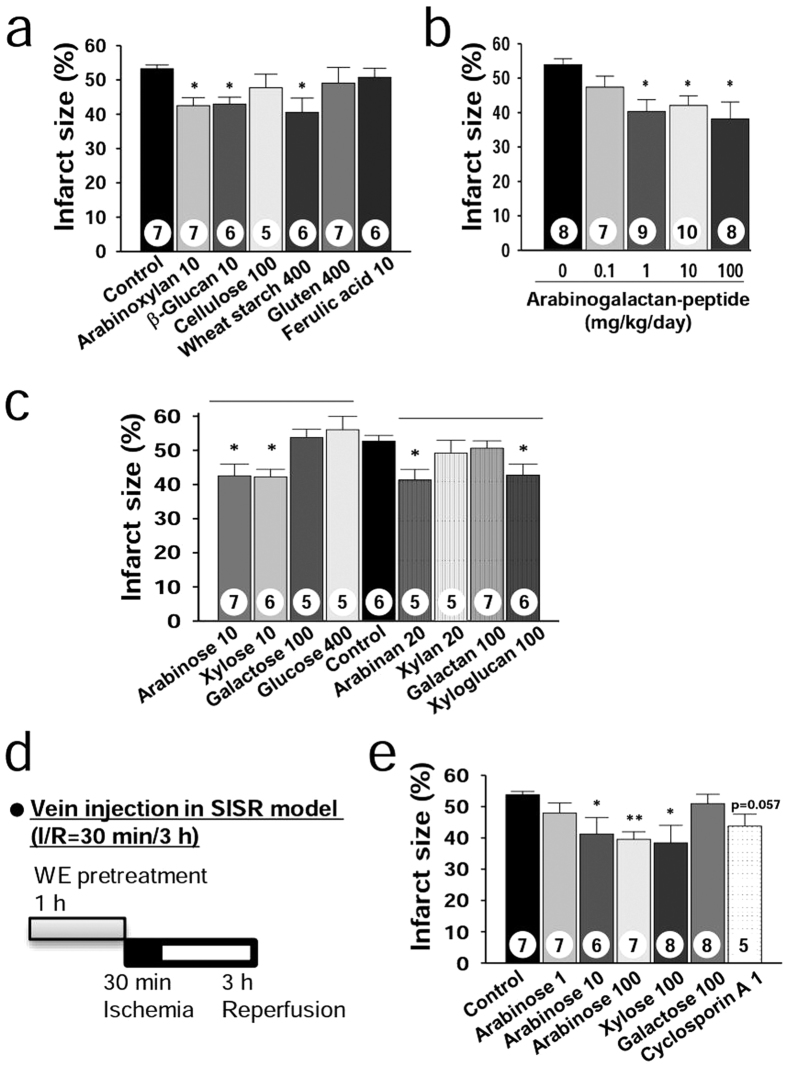
Foods containing specific monosaccharide-rich constituents protect against SISR injury. (**a**) Infarct size for various wheat constituents in the SISR model. The doses for arabinoxylan and β-glucan (10 mg/kg/day) were chosen from Supplementary Fig. S7. The dose for cellulose (100 mg/kg/day) that accounts for 25% of 400 mg/kg/day WE was chosen as the excessive dose because cellulose was not soluble in water. The doses for starch and gluten were chosen by assuming that WE consists of only starch or gluten, based on the fact that starch and protein, which consists mainly of gluten, account for 60–75% and 10–18% of the wheat grains, respectively^63^. The dose of ferulic acid, the major phenolic acid in wheat, was chosen as the excessive dose because the content of ferulic acid in a WE dose of 400 mg/kg/day was calculated as 0.4 mg/kg/day by assuming that ferulic acid accounts for 0.1% of the wheat grains^24^. (**b**) Infarct size for arabinogalactan-peptide. The range of the doses was calculated to include arabinogalactan-peptide content in wheat flour (0.27–0.38%)^64^, which is less than 2 mg/kg/day of arabinogalactan-peptide in 400 mg/kg/day WE. (**c**) Infarct size for various plant cell wall monosaccharides and polysaccharides. The dose of galactose was chosen as the excessive dose by assuming that arabinogalactan-peptide at 100 mg/kg/day consists only of galactose. Data divisions according to polysaccharide and monosaccharide groups are shown as horizontal lines. (**d**) Design of the SISR experiments with samples administered through tail-vein injection 1 hour before LAD artery ligation. (**e**) Infarct size for arabinose, xylose, galactose, and cyclosporine A. *P < 0.05, **P < 0.01, ***P < 0.001 compared with the controls. The numbers inside bars indicate the number of animals per group.

Using the SISR model, we first tested arabinoxylan and β-glucan at various doses ranging from 0 to 10 mg/kg/day (Supplementary Fig. S7). For both arabinoxylan and β-glucan, supplementation over 5 mg/kg/day significantly reduced the IS compared with that of the control group (42.5 ± 2.3% and 43.0 ± 2.0% vs. 53.2 ± 1.1%, respectively) (Fig. 6a, Supplementary Fig. S7). For cellulose, even supplementation at an excessive dose (100 mg/kg/day) was not effective (*P* > 0.05) (Fig. 6a). For starch and gluten at excessive doses (400 mg/kg/day), only starch supplementation significantly reduced the IS compared with the control group (40.5 ± 4.2 vs. 53.2 ± 1.1%) (Fig. 6a). Supplementation with arabinogalactan-peptide over 1 mg/kg/day significantly reduced the IS compared with that of the control group (Fig. 6b). However, supplementation with 10 mg/kg/day of ferulic acid as a sufficient dose was not effective (*P* > 0.05) (Fig. 6a). These results clearly show that water-soluble polysaccharides are active constituents of WE that protect against myocardial injury during the postocclusion steps.

We next addressed how water-soluble polysaccharides reduce myocardial injury when taken orally. Certain residues attached to the backbone may be released from polysaccharides via acid hydrolysis in the stomach^25^ and via glycoside hydrolase (GH) hydrolysis in the large intestine^26^. For example, arabinose can be released from arabinoxylan, and the arabinose generated can be absorbed into the body^27^,^28^. Therefore, we hypothesized that the monosaccharides generated in the stomach and large intestine can be active components. To prove this hypothesis, we first tested supplementation with arabinose and xylose at 0–100 mg/kg/day (Supplementary Fig. S8). Both arabinose and xylose supplementation over 10 mg/kg/day significantly reduced the IS compared with that of the control group (42.6 ± 3.4% and 42.2 ± 2.2% vs. 52.7 ± 1.7%, respectively) (Fig. 6c). However, supplementation with galactose (a component of arabinogalactan-peptide) and glucose (a component of β-glucan and starch) was not effective in reducing IS, even at high doses (100 and 400 mg/kg/day, respectively) (*P* > 0.05) (Fig. 6c). These findings show that the monosaccharide composition of polysaccharides is an important factor that determines treatment efficacy.

To further confirm this hypothesis, we examined three polysaccharides that primarily consist of arabinose (arabinan), xylose (xylan), and galactose (galactan). In addition, we also examined xyloglucan, which is composed of a glucose backbone substituted with xylose residues. Both arabinan (20 mg/kg/day) and xyloglucan (100 mg/kg/day) supplementation were effective in reducing IS compared with the control group (41.3 ± 3.1% and 42.7 ± 3.3% vs. 52.7 ± 1.7%, respectively), whereas xylan (20 mg/kg/day) and galactan (100 mg/kg/day) were not (*P* > 0.05) (Fig. 6c). These findings indicate that xylose linked to the glucan backbone as a substituent is a major contributor to the efficacy of xyloglucan. The findings also indicate that arabinose linked to xylan and galactan backbones as substituents are active components in both arabinoxylan and arabinogalactan-peptide, respectively.

In addition, supplementation with arabinoxylan, β-glucan, arabinose, and xylose significantly reduced the amount of apoptotic cells in the border zone (BZ) at 10 mg/kg/day compared with the control group (2.3 ± 0.9%, 3.9 ± 1.5%, 3.9 ± 2.3% and 4.1 ± 0.7% vs. 11.3 ± 1.5%, respectively) (Supplementary Fig. S9a); however, a reduction was not observed with starch supplementation. Furthermore, arabinogalactan-peptide supplementation significantly reduced the amount of apoptotic cells in the BZ at 100 mg/kg/day compared with the control group (7.4 ± 1.4% vs. 14.2 ± 2.2%) (Supplementary Fig. S9b). These findings indicate that apoptosis inhibition through the active components in WE also contributes to myocardial protection.

Finally, arabinose and xylose were administered through tail-vein injection in the SISR model (Fig. 6d), and both treatments at a dose of 100 mg/kg significantly reduced IS compared with the control group (39.5 ± 2.4% and 38.4 ± 5.6% vs. 53.8 ± 1.1%, respectively) (Fig. 6e). These results clearly show that the arabinose and xylose generated and absorbed into the body contribute to myocardial protection. As a positive control, we further examined cyclosporine A, an experimental myocardial protectant. Tail-vein injection with cyclosporine A at 1 mg/kg 29 tended to reduce the IS compared with the control group (P = 0.057) (Fig. 6e). One plausible reason for the lack of efficacy might be due to the treatment scheme (one bolus injection at −1 hour of occlusion in this study vs. 15 minutes infusion at 5 minutes in ref. 27). These results indicate that arabinose and xylose show similar efficacies as cyclosporine A under our experimental conditions.

### Plant-based foods rich in complex polysaccharides that primarily contain arabinose and/or xylose as monosaccharides can prevent CHD

**Figure 7:**
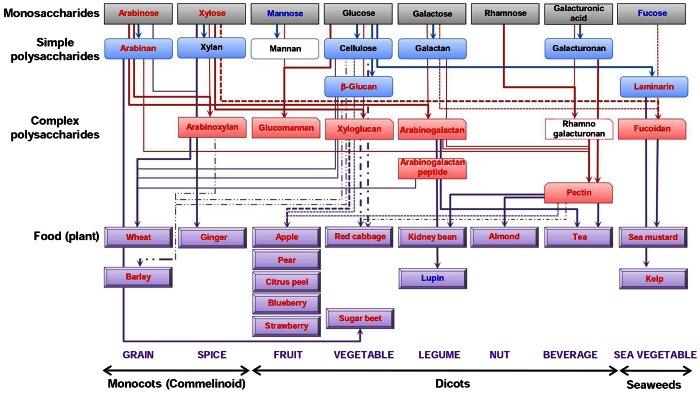
Summary of the protective effects of various foods and their constituents. The data for the cell wall monosaccharides, cell wall polysaccharides, and food components (classified as commelinoid monocots, dicots, and seaweeds) are presented. Food components were broadly classified into land plants, including monocots (commelinoid) and dicots, and algae, including seaweeds, because they all contained different polysaccharide compositions. We performed a literature search and selected the following food components that contain high arabinose and/or xylose levels as candidates for testing. For the commelinoid monocots, wheat and barley were selected as representative grains, and ginger was selected as a spice. For the dicots, apple, pear, blueberry, strawberry, and citrus peel were selected as representative fruits; red cabbage and sugar beet were selected as vegetables; kidney bean and lupin were selected as legumes; almond was selected as a nut; and tea was selected as a beverage. Among the sea vegetables, kelp and sea mustard were selected because they contain a high fucose content as a constituent of fucoidan. Red, blue, and black indicate significant, weak, and no efficacy, respectively. Constituents with white backgrounds were not tested.

We further investigated several more samples with different complexities, and the results are summarized in Fig. 7. At the monosaccharide level, we tested mannose, rhamnose, galacturonic acid, and fucose in addition to those already tested (arabinose, xylose, glucose, and galactose) using the SISR model. Of the eight monosaccharides tested, arabinose and xylose were effective (as demonstrated above), and supplementation with D-mannose and L-fucose (10 mg/kg/day) tended to reduce the IS compared with that of the control group, although the difference was not statistically significant (43.9 ± 5.0% (*P* = 0.143) and 45.1 ± 3.4% (*P* = 0.069) vs. 52.9 ± 2.16%, respectively) (Supplementary Fig. S10a). These results indicate that arabinose and xylose are the best monosaccharides for myocardial protection.

At the polysaccharide level, more commercially available cell wall polysaccharides derived from plant and seaweed cell walls were examined. At the simple polysaccharide level, we tested galacturonan and laminarin (Table S4) in addition to those already tested (arabinan, xylan, cellulose, β-glucan, and galactan). Supplementation with laminarin at 100 mg/kg/day significantly reduced the IS compared with the control group (42.5 ± 4.0% vs. 53.8 ± 1.1%) (Supplementary Fig. S10b). Of the simple polysaccharides tested, we found that arabinan (a domain of pectin), β-glucan, and laminarin provided effective myocardial protection. At the complex polysaccharide level, we examined glucomannan, arabinogalactan, and fucoidan (Table S4) in addition to those already tested (arabinoxylan, xyloglucan, and arabinogalactan-peptide) and another already published (pectin)^10^. Supplementation with glucomannan, arabinogalactan, and fucoidan significantly reduced the IS compared with the control group (43.8 ± 2.3%, 42.6 ± 2.6% and 43.7 ± 3.8% vs. 53.8 ± 1.1%, respectively) (Supplementary Fig. S10b). Of the complex polysaccharides tested, those composed of more than one active monosaccharide, such as arabinose, xylose, mannose, and/or fucose, provided effective myocardial protection.

At the food component level, we examined food components containing a high content of active polysaccharides. We performed a literature search and selected representative food components in each food category that contain high arabinose, xylose, and/or fucose levels^30^,^31^,^32^,^33^,^34^,^35^,^36^,^37^. After samples were prepared with the most appropriate cooking methods (Supplementary Fig. S11), rats were supplemented with 400 mg/kg/day of extracted supernatant for wheat and barley and 400 mg/kg/day of extracted whole for the remaining food components. Supplementation with all of the food components except lupin significantly reduced the IS compared with that of the control group (Supplementary Fig. S12). These findings indicate that food components containing high levels of arabinose, xylose, and/or fucose as the constituents of cell wall polysaccharides are effective in protecting against myocardial injury during the postocclusion steps.

## Discussion

**Figure 8:**
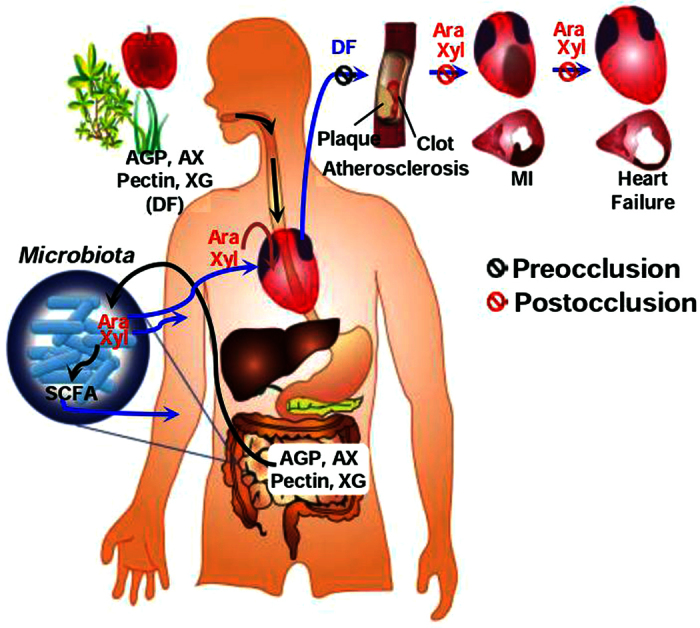
Summary of the steps involved in CHD development and the proposed roles played by plant-based diets in preventing CHD. CHD development can be divided into the preocclusion and postocclusion steps. Traditionally, plant-based diets have been thought to delay artery occlusion through inhibition of atherosclerosis (preocclusion steps). Here, we demonstrated that plant-based diets protect the heart after the onset of occlusion (postocclusion steps); when we consume plant-based diets, we also ingest plant cell wall polysaccharides constituting the diets. Arabinose and xylose generated from the hydrolysis of polysaccharides by microbiota inhabiting the large intestine and absorbed into the body protect the heart against ischaemic injury. Subsequently, they may also be involved in inhibiting the development of heart failure through mitigation of adverse left-ventricular remodelling (See the text for more details). AGP, arabinogalactan-peptide; AX, arabinoxylan; XG, xyloglucan; DF, dietary fibres; SCFA, short-chain fatty acids; MI, myocardial infarction.

In this study, we provided two new aspects of plant-based diets. First, several specific monosaccharides, such as arabinose, xylose, and possibly fucose, constituting cell wall polysaccharides, the major ingredient of dietary fibre, were identified as active constituents in an acute MI rat model, thus acting as cardioprotectants by inhibiting postocclusion steps (Fig. 8). Consequently, plant food components rich in active monosaccharides were effective in the same acute MI rat model. Second, WE protected against myocardial injury in the acute MI rat model and also mitigated adverse LV remodelling, a predictor of heart failure. Consistent with this conclusion, WE, arabinoxylan, and arabinose reduced brain injury in a chronic hypoperfusion rat model^38^,^39^.

Various previous cohort studies have shown that high consumption of food components, such as whole grains^40^, fruits^41^, vegetables^42^, legumes (including beans)^43^, nuts^44^, and tea^45^ is associated with a reduced risk of CHD mortality. The food components confirmed in the previous studies are generally consistent with those identified in this study. In previous studies, however, no specific constituents in the food components were identified as responsible for the reduced CHD risk. In this study, we identified cell wall polysaccharides rich in specific monosaccharides as the key constituents. Our findings may explain some inconsistencies observed between dietary fibre sources and CHD risk in previous cohort studies. For example, higher cereal fibre intake was associated with reduced CHD risk in all three publications in which meta-analyses of cohort studies were performed^46^,^47^,^48^, with fruit fibre intake in two publications^46^,^48^ and vegetable fibre intake in only one publication^47^. This difference in the efficacy depending on fibre sources can be explained through active polysaccharide and active monosaccharide content. In the active polysaccharide content, parenchymatous tissues of cereals contain higher active polysaccharides (β-glucan plus arabinoxylan: 80–85wt% of cell walls) than those of fruits and vegetables (pectin plus xyloglucan: 45–50wt%)^30^. In the active monosaccharide content, wheat endosperm (cereal) contains higher active monosaccharides (arabinose plus xylose) than apple (fruit). Apple, in turn, contains higher active monosaccharides than potato and onion (vegetables)^30^. In the same context, apple, pear, berries, and peach (fruits) contain higher active monosaccharides than cucumber and squash (vegetables)^32^. Taken together, a higher intake of active polysaccharides and active monosaccharides is associated with reduced CHD risk.

Our findings can be applied to the preparation of better plant-based diets. Although food components were classified according to food categories (grains, fruits, and vegetables in Mediterranean and pescetarian diets^3^ and fruits, vegetables, tea, and seaweeds in a Japanese dietary pattern^49^), no further sub-classification has yet been proposed. We propose that, for maximal efficacy, food components in each food category that contain larger amounts of active polysaccharides and active monosaccharides should be included.

Regarding the underlying mechanisms for preventing CHD, the intake of plant-based diets and their food components was presumed to show efficacy by inhibiting the preocclusion and postocclusion steps in previous studies and the present study, respectively. This difference provides different approaches for developing CHD prevention strategies; to delay atherosclerosis, it is important to ingest certain amounts of dietary fibre each day for a long period because it takes several decades for atherosclerotic plaques to form^50^. To reduce ischaemic myocardial injury, however, it is crucial to ingest dietary fibre as frequently as possible to maintain blood active monosaccharide concentrations above threshold values when arteries are abruptly occluded. Taken together, frequency and amount of dietary fibre intake are important considerations in maximizing efficacy.

From an in-depth study with WE, we found implications of plant-based diets in several clinical settings. In rat MI models with reperfusion, WE intake initiated apoptosis inhibition through the removal of oxidative stress, a crucial trigger of apoptosis^51^. ROS generation was reduced by up-regulation of fatty-acid binding protein^52^,^53^ and myoglobin^54^ in the cytosol and by down-regulation of monoamine oxidase^55^ in the mitochondria. Inhibition of ROS generation was confirmed through reduction of MDA levels in the myocardial tissue, a surrogate biomarker for ROS^15^. Reduction of ROS generation ultimately inhibited apoptosis through various putative apoptotic pathways, which are linked to MAPK, the Bcl-2 family, and caspase-3. These results are also supported by our previous findings in which WE treatment attenuated ROS production and subsequent apoptosis caused by β-amyloid in human blastoma cells^56^. In addition, WE intake contributed to maintaining ATP levels through enhancing carbohydrate and fat metabolism and the conversion of energy intermediates to ATP. WE intake also contributed to maintaining protein homeostasis through enhancing the synthesis and degradation of protein and the cytoskeleton. As a result, apoptosis inhibition and maintenance of high ATP levels and protein homeostasis synergistically led to myocardial protection. Myocardial protection in the early phase of reperfusion by WE intake resulted in the mitigation of adverse LV remodelling. Therefore, prophylactic WE intake by patients at high risk of developing MI protects the heart against IR injury and inhibits consequent progression to heart failure in a clinical setting in which occluded arteries are opened through reperfusion therapy, such as percutaneous coronary angioplasty^57^. In a rat MI model without reperfusion, WE intake also protected the heart by inhibiting apoptosis in the early phase of ischaemia, which subsequently mitigated adverse LV remodelling. These findings indicate that prophylactic WE intake is beneficial, even for those who are ineligible for reperfusion therapy, such as those who arrive at a hospital more than 12 hours after symptom onset^58^. Taken together, prophylactic WE intake may be beneficial in all emergency settings of heart attack. It is of interest to determine whether other active food components in plant-based diets have similar efficacies as WE.

## Methods

### Supplements

L-Arabinose, D-xylose, D-mannose, D-glucose, D-galactose, L-rhamnose, D-galacturonic acid, L-fucose, xylan (oat spelts) (xylose:arabinose:glucose = 75:10:15), cellulose (cotton linters), laminarin (*Laminaria digitate*), starch (wheat), and enalapril were purchased from Sigma-Aldrich. Arabinan (sugar beet) (arabinose:galactose:rhamnose: galacturonic acid = 88:3:2:7), galactan (potato) (arabinose:galactose:rhamnose: galacturonic acid = 87:3:3:7), galacturonan (citrus pectin) (galacturonic acid:xylose:galactose:rhamnose = 96:1:1:1.2), β-glucan (barley), arabinoxylan (wheat) (arabinose:xylose = 38:62), glucomannan (Konjac) (glucose:mannose = 40:60), and xyloglucan (tamarind seed) (xylose:glucose:arabinose:galactose = 34:45:3:18) were purchased from Megazyme. Fucoidan (*Undaria pinnatifida*) (fucose:galactose:glucose:xylose = 50:48:1:1) was kindly provided by Professor Kim at Catholic University of Daegu (Daegu, Korea). Arabinogalactan-peptide was prepared from wheat flour as previously described^59^. The neutral monosaccharide composition (wt/wt) of arabinogalactan-peptide was 40.0% arabinose, 57.7% galactose, 0.8% xylose, 1.2% glucose, and 0.3% mannose. WE containing water soluble ingredients was analysed and determined to be composed of 4.9% water, 5.9% ash, 7.9% protein, 0.2% fat, and 81.1% carbohydrate, including 15.4% total dietary fibre (wt%). The neutral monosaccharide composition of WE was 1% arabinose, 1.6% xylose, 0.7% galactose, and 77.1% glucose (wt%). The composition of neutral monosaccharides in the arabinogalactan-peptide and WE was assessed as previously described^9^.

### Preparation of food component samples

Sugar beets and tea were provided by the Agricultural Research Center for Climate Change (Jeju, Korea) and the Institute of Hadong Green Tea (Gyeongnam, Korea), respectively. Wheat (variety: Kumkang) was purchased from Woorimil (Jeonnam, Korea). All of the other food components were purchased locally. The WE was prepared as previously described^56^. Briefly, whole wheat grains were ground in a mill, and the ground grains were extracted in 10-fold dilutions of hot water at approximately 90 °C for 1 hour. The whole extract was centrifuged at 14,000 × *g* for 30 minutes, and the supernatant was concentrated and freeze-dried to yield WE (i.e., extracted supernatant). In addition, the whole extract after the hot water extraction was directly freeze dried without centrifugation (extracted whole); the whole wheat grains were steam-cooked and then freeze-dried (steamed whole grains) or steam cooked, ground with a mixer (HM-5000, Hyun Dae Household Appliances Co., Ltd., Incheon, Korea), and then freeze dried (steamed ground grains). The barley extract was similarly prepared. All of the other food samples except the wheat and barley grains were prepared as follows: raw food samples were cut into pieces if necessary, homogenized in a mixer, and extracted with 10–20-fold dilutions of hot water for 1 hour. The whole extracts were freeze dried without centrifugation.

### Myocardial experiments

The animal experiments were conducted according to the guidelines for the animal care and use of laboratory animal protocols approved by the Institutional Animal Care and Research Advisory Committee of Catholic University of Daegu. Myocardial injury was generated through LAD coronary artery ligation in male Sprague-Dawley rats (250–300 g, 8 weeks) (Samtaco Inc., Osan, Korea) as previously described^10^,^60^. The rats were anaesthetized with intramuscular injections of ketamine (100 mg/kg) and xylazine (5 mg/kg), intubated, and ventilated with air. The heart was exposed by a left thoracic incision, and the LAD artery was ligated approximately 5 mm down from the aortic origin by passing a 5–0 Prolene suture (BV-1, Ethicon) around the LAD artery and then double-knotting the suture. Whether the LAD artery was occluded or not was confirmed by observing LV wall alterations via the development of a pale colour. Subsequently, IR or ischaemia-only experiments were performed as follows. In the SISR experiments, the hearts were ligated for 30 minutes and then reperfused for 3 hours. In the SILR experiments, the hearts were ligated for 30 minutes and then reperfused for 7 days. In the LI experiments, the heart remained ligated for up to 30 days. IS was assessed through 2,3,5-triphenyltetrazolium chloride (TTC) (Sigma-Aldrich) or haematoxylin & eosin (H&E) staining for the SISR and LI experiments, respectively. In the case of TTC staining, the LAD was religated. Then, 1 ml of 1.0% Evans blue (Sigma-Aldrich) was infused through the jugular vein; subsequently, the AAR was defined as the area that was not infiltrated by the dye. The heart was excised into 4 pieces approximately 3-mm thick, and the pieces were stained in 1 ml of 1.5% TTC at 23 °C for 5 minutes. Subsequently, IA was defined as the area that was not stained by TTC. BZ was defined as the region in which the IA is excluded from the AAR. AAR and IA were determined by computerized planimetry using ImageJ software (NIH, v1.47), and IS was defined as a percentage of the IA relative to the AAR. In the case of H&E staining, the pieces were fixed, sectioned on 5-μm thick slides, and stained with H&E. The IA was identified by viewing the sections under a microscope, and the IS was calculated as a percentage of the IA relative to the LV area. To assess the average infarcted LV wall thickness, five evenly spaced radians were passed through the infarct with the centre of the LV cavity as a reference, and the average infarcted LV wall thickness was calculated. To determine the average non-infarcted LV wall thickness, the septal LV wall thickness was measured in a similar manner. To quantify the infarct expansion, the expansion index (EI), which accounts for LV dilation and infarct wall thinning, was employed, for which LV cavity area was also determined by computerized planimetry^21^.


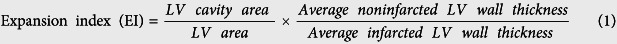


For the oral sample administration, rats were randomly assigned to sham, control, or sample-treated groups. In the sample-treated group, the rats received the samples supplemented at various doses with a standard chow diet for 3 days prior to occlusion. For the tail-vein injection sample administration, the rats received the samples once 1 hour prior to occlusion. In the control group, the rats received the standard chow diet only. In the sham group, the experimental procedures were the same as those in the control group but without ligation.

Assessments of IS, apoptotic cells, LV wall thickness, collagen content, and expansion index were performed as previously described^9^,^10^,^21^. The MDA levels in the AAR were measured as previously described^61^. Detailed procedures are provided in Supplementary Methods.

### Quantitative proteomics

For the preparation of proteome tissue samples, the AAR was incised from the harvested hearts after the infusion of Evans blue in the SISR model. The AAR was snap frozen in liquid nitrogen and stored at −80 °C until analysis. Frozen AAR tissue (approximately 100 mg) was homogenized in 0.5 ml of lysis buffer [8 M urea, 4% CHAPS, 5 mM magnesium acetate, and 30 mM Tris (pH 8.5)] using Precellys 24 (Bertin Technologies) at a frequency of 5,800 per minute for 20 seconds and repeated three times with a 3-minute pause in between to minimize heat generation. The supernatant was separated by centrifugation at 20,800 × *g* for 30 minutes at 4 °C. Protein concentrations were determined using a Bradford protein assay (Bio-Rad), and the aliquots were stored at −80 °C. Protein extracts (100 μg) from each heart tissue were subsequently tagged for quantitative mass spectrometry using a TMT sixplex reagent kit (No. 90064, Thermo Scientific) following the manufacturer’s protocol (See the Supplementary Methods for more details). The TMT-labelled samples were analysed using a 2D-LC-MS/MS system consisting of a nanoACQUITY UltraPerformance LC System (Waters) and an LTQ Orbitrap Elite mass spectrometer (Thermo Scientific) equipped with a nanoelectrospray source. The MS/MS spectra were analysed using the IPI rat database (IPI.Rat_v387. 9.11.2012) with the reversed sequences of all proteins. A detailed description of the 2D-LC-MS/MS analysis and protein identification and quantification can be found in our previous study^62^ and in Supplementary Methods for more details.

### Western blots

The levels of selected proteins of interest were verified using Western blot analyses. Western blotting and analysis were performed using standard protocols (See Supplementary Methods for more details). Immunoblotting was performed using the protein extracts from batches of rat heart tissues, which were different from those used for the TMT labelling experiments. Detailed information on the specific antibodies used is provided in Table S5.

### Statistical analysis

The sample size in the MI experiments was pre-determined using a power analysis (G*Power 3.1.7 program) as previously described^9^. The data are expressed as the mean ± SEM. All of the statistical analyses were performed in SPSS (IBM Corporation, v19). For all of the data, Shapiro–Wilk and Levene statistics were applied a priori to verify the normality and homogeneity of variances, respectively. The data that met the assumptions for a one-way ANOVA were analysed using ANOVA with post hoc tests. Non-normal datasets were analysed by the Kruskal–Wallis test using a post hoc pairwise comparison of mean ranks. Between two groups, an unpaired two-sided Student’s t-test was used. Differences between the control and sample-treated groups were considered statistically significant at *P* < 0.05.

## Additional Information

**How to cite this article**: Lim, S. H. *et al*. Plant-based foods containing cell wall polysaccharides rich in specific active monosaccharides protect against myocardial injury in rat myocardial infarction models. *Sci. Rep.* **6**, 38728; doi: 10.1038/srep38728 (2016).

**Publisher's note:** Springer Nature remains neutral with regard to jurisdictional claims in published maps and institutional affiliations.

## Supplementary Material

Supplementary Information
