# Supplementary material for: Plant-based foods containing cell wall polysaccharides rich in specific active monosaccharides protect against myocardial injury in rat myocardial infarction models
Source: Sci Rep. 2016 Dec 8;6:38728. doi: 10.1038/srep38728 (PMC5143980; doi:10.1038/srep38728)
Supplement: Supplementary Information [file srep38728-s1.pdf]

Supplementary Information

Plant-based foods containing cell wall polysaccharides rich in specific active monosaccharides protect against myocardial injury in rat myocardial infarction models

Sun Ha Lim[1], Yaesil Kim[2], Ki Na Yun[3,4], Jin Young Kim[3], Jung-Hee Jang[5], Mee-Jung Han[6,*] & Jongwon Lee[1,*]

[1]Department of Biochemistry, School of Medicine, Catholic University of Daegu, Daegu 42472, Korea. [2]Hypoxi Co., LTD., Daegu 42472, Korea. [3]Biomedical Omics Group, Korea Basic Science Institute, Ochang 28119, Korea. [4]Sogang University, Seoul 04107, Korea. [5]Department of Pharmacology, School of Medicine, Keimyung University, Daegu 42601, Korea. [6]Department of Biomolecular and Chemical Engineering, Dongyang University, Yeongju, Gyeongbuk 36040, Korea. *Correspondence and requests for materials should be addressed to J.L. (email: leejw@cu.ac.kr) or M.-J.H. (email: mjhan75@dyu.ac.kr).

Supplementary methods

Cell viability assessment and DNA fragmentation assay. Cell viability was assessed, as previously described[1]. Briefly, human hepatocellular carcinoma cells (HepG2) were plated at 2×10^5 cells in minimum Eagles's medium (MEM) supplemented with 10% fetal bovine serum (FBS, Gibco BRL) for 48 h under normoxic condition. The culture medium was exchanged with a fresh medium containing 100 or 1,000 μg/ml of the WE pre-dispersed in 50% ethanol, or that containing no WE (Control), respectively. Then the cells were cultured further either under normoxic (5% CO_2, balanced with air) or hypoxic (1% O_2, 5% CO_2, balanced with N_2) condition in a humidified chamber (Vision Scientific Co., Daejeon, Korea) for another 48 h. At 1 and 2 days of cell culture, viable cells were qualitatively assessed with a colorimetric method, 3-(4,5-dimethylthiazol-2-yl)-2,5-diphenyltetrazolium bromide (MTT) assay or quantitatively with 0.4% trypan blue dye solution and counted using a hemocytometer. Glucose concentrations were also measured with the hexokinase method on the Hitachi 747 analyzer (Roche Diagnostics,). For DNA fragmentation assay, the HepG2 cells were plated at 1×10^6 cells and cultured under hypoxic conditions in the presence of WE (400 μg/ml) or in the absence of WE (Control), as described above. The viable cells at various time points of cell culture were counted with trypan blue dye method, and also lysed in the lysis buffer containing 0.5% Triton buffer. DNA obtained from the lysate was electrophoresed on a 1.5 % agarose gel.

Determination of the neutral monosaccharide composition. The composition

of neutral monosaccharides in the arabinogalactan-peptide and WE was assessed as previously described[2]. Briefly, purified arabinogalactan-peptide and WE (10 and 20 mg samples, respectively) were hydrolyzed with 2 M trifluoroacetic acid, reduced with 0.5 M sodium borohydride, and acetylated with 2 ml of acetic anhydride to yield samples containing alditol acetates of the corresponding monosaccharides. The alditol acetate composition was analyzed using gas chromatography (GC).

Assessment of myocardial cell hypertrophy. The cross-sectional areas of cardiomyocytes were evaluated in histological sections. The LV sections stained with H&E were captured on a digital microscope (400×) (Olympus). Photomicrographs were taken from the BZ and remote zone. To maintain consistency among the results, the cardiomyocytes positioned perpendicularly to the plane of the section with visible nuclei and with cell membranes that were clearly outlined and unbroken were then selected for the cross-sectional area measurement. The cross-sectional areas of the cardiomyocytes were traced with ImageJ software. From each heart, 100 cardiomyocytes from the BZ and remote zone were each assessed.

Assessment of the MDA levels in myocardial tissues. The MDA levels in the AAR were measured as previously described[3]. The heart tissue was weighed and homogenized in a solution containing 5 mM butylated hydroxytoluene, and the MDA levels were then measured using BIOXYTECH MDA-586 kits (OxisResearch). The relative MDA levels in the control and WE-treated groups were assessed by setting the MDA level of the sham group to 1.

TUNEL staining. TUNEL staining was performed as previously described[4].

Briefly, the heart sections were stained using the in situ Cell Death Detection Kit (Roche Diagnostic) according to the manufacturer's protocol. For positive controls, the sections were treated with DNase I before TUNEL staining. For negative controls, TUNEL staining was performed in the absence of terminal deoxynucleotidyl transferase. For counter staining, the sections were stained with methyl green. To assess the percentage of apoptotic cells, micrographs of the TUNEL-positive nuclei and methyl green-stained nuclei were captured using an Olympus microscope and counted using ImageJ in 10 to 20 random fields at 400× magnification.

Picrosirius staining. The heart slides were rinsed with Milli-Q water and incubated with 0.1% Sirius red F3BA (Direct Red 80, Sigma-Aldrich) in saturated picric acid for 1 hour. The slides were then rinsed with acidified water (0.5% acetic acid) twice and with 0.01 N HCl for 1 minute twice and then immersed in Milli-Q water. After dehydration with 70% ethanol for 30 seconds, the slides were visualized. The cardiac collagen density (%) was expressed as the ratio of the collagen-positive area to the left ventricular area.

Immunohistochemical staining. Cleaved caspase-3 levels in the heart sections were assessed as previously described[4]. Briefly, the heart sections were pretreated at boiling temperatures, incubated with hydrogen peroxide, bovine serum albumin, and goat serum subsequently treated with anti-cleaved caspase-3 primary antibody (1:50 dilution, Cell Signaling Technology) and stained using a Vectastain Elite ABC kit (Vector Laboratories) according to the manufacturer's instructions. Color was developed with 3,3′-diaminobenzidine tetrahydrochloride (DAB; Roche Life Science). The sections were evaluated at a 200× magnification.

The intensity of DAB staining was measured using ImageJ. The caspase-3 levels were assessed by calculating the relative color intensity of the image to that of the sham group, and the color intensity of the latter was set to 1.

TMT labeling. The protein extracts (100 µg) from each heart tissue were reduced with 500 mM Tris(2-carboxyethyl)phosphine (TCEP) at room temperature for 1 hour and then alkylated with 500 mM iodoacetamide (IAA) at room temperature in the dark for 1 hour. The samples were desalted using a membrane filter of 10 kMW and dissolved in 200 mM triethylammonium bicarbonate (TEAB) buffer to a final concentration of 1 µg/µl. Sequencing-grade trypsin (Promega) was added at 1:20 (w/w) to the proteins in TEAB buffer and incubated overnight at 37°C. Each peptide sample of individual proteins extracted from the sham, control, and WE-treated rats was divided into two fractions and individually labeled using a TMT sixplex reagent kit (No. 90064, Thermo Scientific) following the manufacturer's protocol as follows: TMT-126 and TMT-129 for the sham, TMT-127 and TMT-130 for the control, and TMT-128 and TMT-131 for the WE-treated rats. Aqueous hydroxylamine solution (5% w/v) was added to quench the reaction. The six samples were then combined, speed vacuum dried, and then dissolved in 50 µl of Milli-Q water containing 0.1% formic acid for the 2D-LC-MS/MS analysis.

2D-LC-MS/MS analysis. The TMT-labelled samples were analysed using a 2D-LC-MS/MS system consisting of a nanoACQUITY UltraPerformance LC System (Waters) and an LTQ Orbitrap Elite mass spectrometer (Thermo Scientific) equipped with a nano-electrospray source. A detailed description of the 2D-LC-MS/MS analysis can be found in our previous study[5]. Briefly, a strong cation

exchange (SCX, 5 μm, 3 cm) column was used for peptide fractionation over a salt gradient. The fractionated peptides were separated on a 200-mm homemade C18 capillary column (Aqua; 3-μm particle size, 100-μm silica tubing, and 5-μm orifice id). The MS data were acquired in five data-dependent collision-induced dissociation high-energy collision dissociation (CID-HCD) dual MS/MS scans per full scan.

Protein identification and quantification. The MS/MS spectra were analyzed using the IPI rat database (IPI.Rat_v387. 9.11.2012) with the reversed sequences of all proteins. ProLucid[6] was used to identify the peptides with a precursor mass error of 25 ppm and a fragment ion mass error of 600 ppm. Trypsin was selected as the enzyme, which has three potential missed cleavages. TMT modification (+ 229.1629) at the N-terminus and lysine residues and carbamidomethylation at the cysteine residues were chosen as the static modifications. Oxidation at the methionine residues was chosen as a variable modification. The CID and HCD tandem MS spectra from the same precursor ions are often combined using software to allow for better peptide identification and quantification[7]. We used our own software in which reporter ions from the HCD spectrum were inserted into the CID spectrum with the same precursor ion as the previous scan. The output data files were filtered and sorted into the protein list by DTASelect[8], which included two or more peptide assignments for a protein identification and a false positive rate of less than 0.01. A quantitative analysis was conducted using Census in the IP2 pipeline (Integrated Proteomics). The intensity at a reporter ion channel for a protein was calculated as the average of this reporter ion's intensities from all constituent peptides in the identified

protein.[9] To control the quality of protein quantification, we labelled the same sample with two different TMT reagents. When comparing different TMT labels for the same sample, the data with more than 30% variation were excluded. The TMT ratios for the proteins were calculated for the average reporter ion intensities over all the peptides assigned to a protein subgroup. DAVID bioinformatics resources (v6.7) was applied for the differentially expressed proteins to obtain the gene ontology (GO) annotations and term enrichment categorizations for various biological processes.[10] Genes of unknown function were not included in the analysis.

Western blots. Aliquots of protein extracts were thawed and separated on 10, 12, or 15% SDS-polyacrylamide gels according to the molecular weight of a target protein. The proteins were transferred to Immobilon-P PVDF membranes (Millipore), the membranes were stained with MemCode reversible protein stain (Pierce Biotechnology) and imaged to verify that the protein loads were uniform and ensure that efficient electrotransfer occurred, and the membranes were destained with Milli-Q water and blocked with non-fat dry milk prior to incubation with each primary antibody. The detailed information (company, catalogue code, dilutions and Research Resource Identifiers (RRIDs)) of antibodies used in this study is described in Table S5. HRP (horseradish peroxidase)-labeled secondary antibodies (Sigma-Aldrich) and Amersham ECL Prime Western Blotting Detection Reagent (GE Healthcare) were used. X-ray film images were background subtracted, and protein bands were quantitated using Discovery Series Quantity One 1-D Analysis software (Bio-Rad, v4.6.9). ERK1 was employed as a loading control.

References

1. Han, H. S. & Lee, J. Attenuation of brain injury by water extract of goat's-beard (Aruncus dioicus) and its ethyl acetate fraction in a rat model of ischemia-reperfusion. *Prev Nutr Food Sci* **16**, 217-223, (2011).
2. Kim, M. Y., Lim, S. H. & Lee, J. Intake of hot water-extracted apple protects against myocardial injury by inhibiting apoptosis in an ischemia/reperfusion rat model. *Nutr Res* **34**, 951-960, (2014).
3. Baek, H. S., Lim, S. H., Ahn, K. S. & Lee, J. Hot water extract of Triticum aestivum L. (common wheat) ameliorates renal injury by inhibiting apoptosis in a rat model of ischemia/reperfusion. *Kor J Herbology* **28**, 7-15, (2013).
4. Lim, S. H. & Lee, J. Methanol Extract of Cassia mimosoides var. nomame Attenuates Myocardial Injury by Inhibition of Apoptosis in a Rat Model of Ischemia-Reperfusion. *Prev Nutr Food Sci* **17**, 177-183, (2012).
5. Hong, I. *et al.* Quantitative proteomic analysis of the hippocampus in the 5XFAD mouse model at early stages of Alzheimer's disease pathology. *J Alzheimers Dis* **36**, 321-334, (2013).
6. Carvalho, P. C. *et al.* YADA: a tool for taking the most out of high-resolution spectra. *Bioinformatics* **25**, 2734-2736, (2009).
7. Li, Z. *et al.* Systematic comparison of label-free, metabolic labeling, and isobaric chemical labeling for quantitative proteomics on LTQ Orbitrap Velos. *J Proteome Res* **11**, 1582-1590, (2012).
8. Tabb, D. L., McDonald, W. H. & Yates, J. R., 3rd. DTASelect and Contrast:

tools for assembling and comparing protein identifications from shotgun proteomics. *J Proteome Res* **1**, 21-26, (2002).

9. Raso, C. *et al.* Characterization of breast cancer interstitial fluids by TmT labeling, LTQ-Orbitrap Velos mass spectrometry, and pathway analysis. *J Proteome Res* **11**, 3199-3210, (2012).

10. Huang da, W., Sherman, B. T. & Lempicki, R. A. Systematic and integrative analysis of large gene lists using DAVID bioinformatics resources. *Nat Protoc* **4**, 44-57, (2009).

11. Knudsen, K. E. Fiber and nonstarch polysaccharide content and variation in common crops used in broiler diets. *Poult Sci* **93**, 2380-2393, (2014).

Supplementary Figures

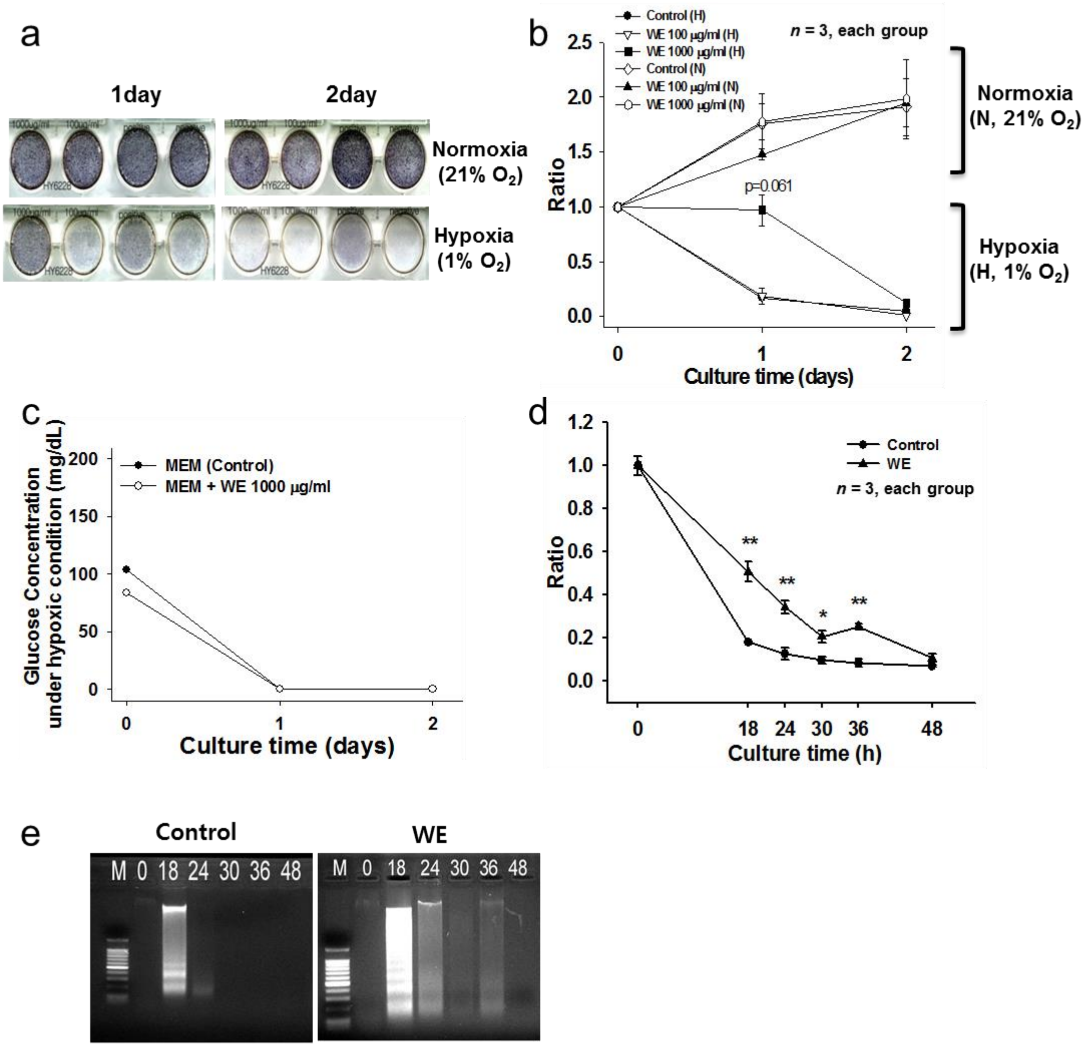

Supplementary Figure S1. WE treatment improves cell survival by inhibiting apoptosis under ischemic conditions

(**a** to **c**) Improvement of cell survival by WE under ischemic conditions. HepG2 cells were cultured under normoxic (21% O_2) and hypoxic (1% O_2) conditions. Live cells were assessed qualitatively by MTT assay (**a**) and quantitatively by cell counting (**b**). The glucose concentrations in the medium were also measured (**c**). At day 1 of culture under hypoxic condition, the medium became ischemic (hypoxia and hypoglycemia) (**c**), under which condition WE treatment (1,000 μg/ml) improved cell survival without inhibiting cell proliferation (**b**). (**d** and **e**) Apoptosis inhibition under ischemic conditions. WE treatment (400 μg/ml) also improved cell survival under ischemic condition (**d**) and maintained the appearance of a DNA ladder longer (**e**). The findings suggest that WE treatment improves cell survival by inhibiting apoptosis. *$P < 0.05$, **$P < 0.01$ compared to controls.

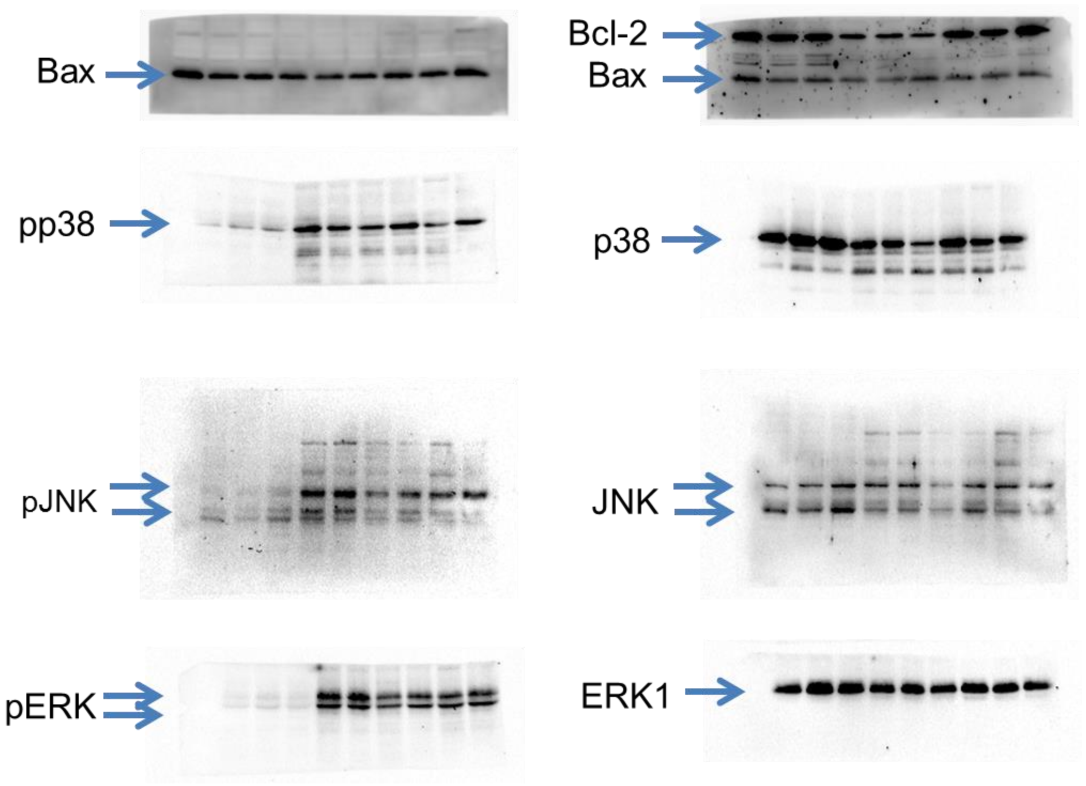

Supplementary Figure S2. The original bands for Figure 1f and g in the main manuscript.

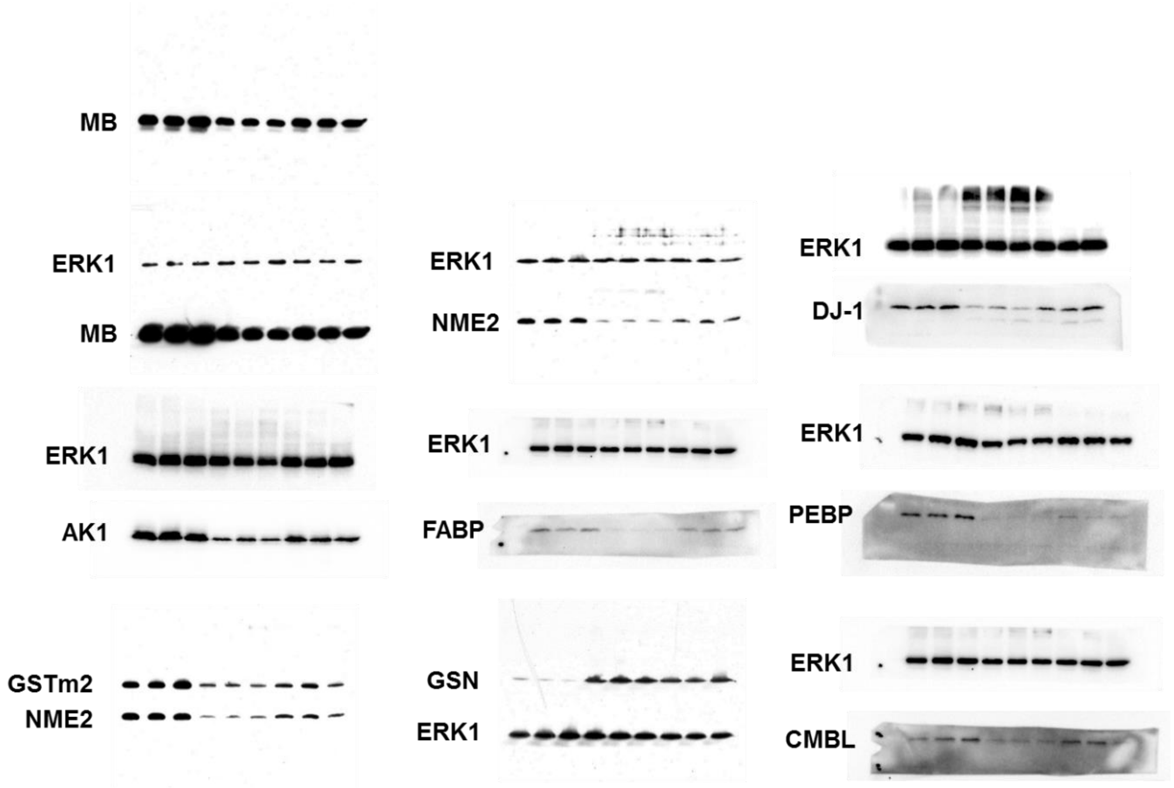

Supplementary Figure S3. The original bands for Figure 2c in the main manuscript.

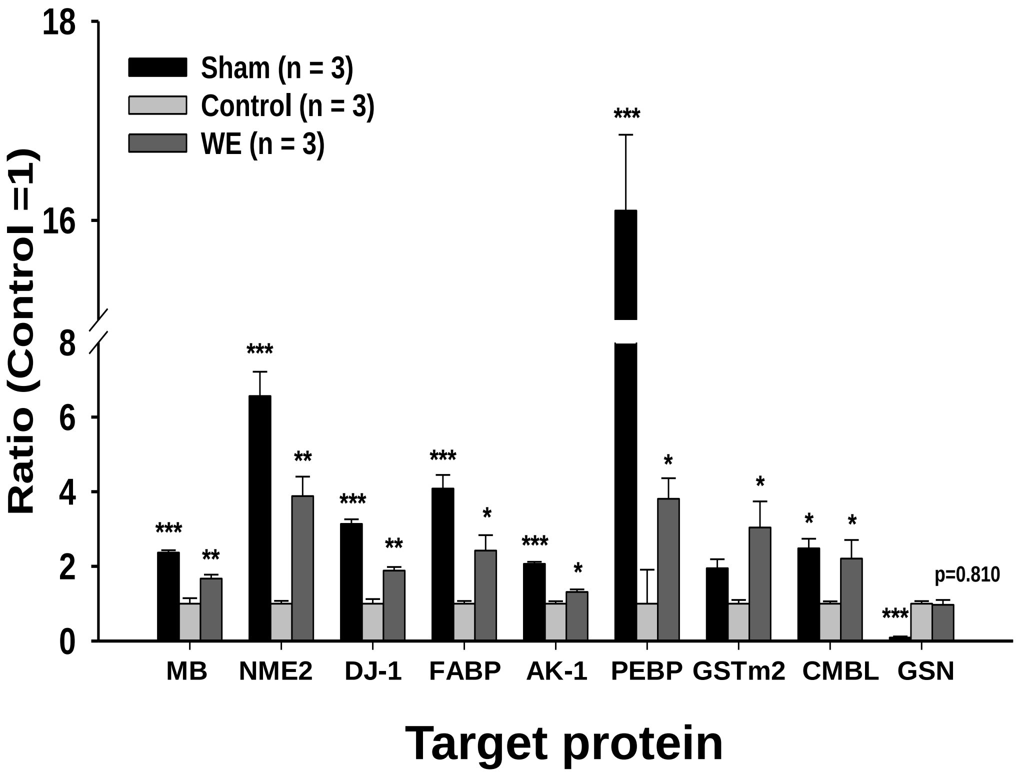

Supplementary Figure S4. Histogram of the quantitative Western results of 9 candidates from biological triplicate

See the text for more details and the legend of Figure 2c. The ratio of each protein in Sham and WE was calculated by setting that of the control group 1. *$P < 0.05$, **$P < 0.01$, ***$P < 0.001$ compared to controls.

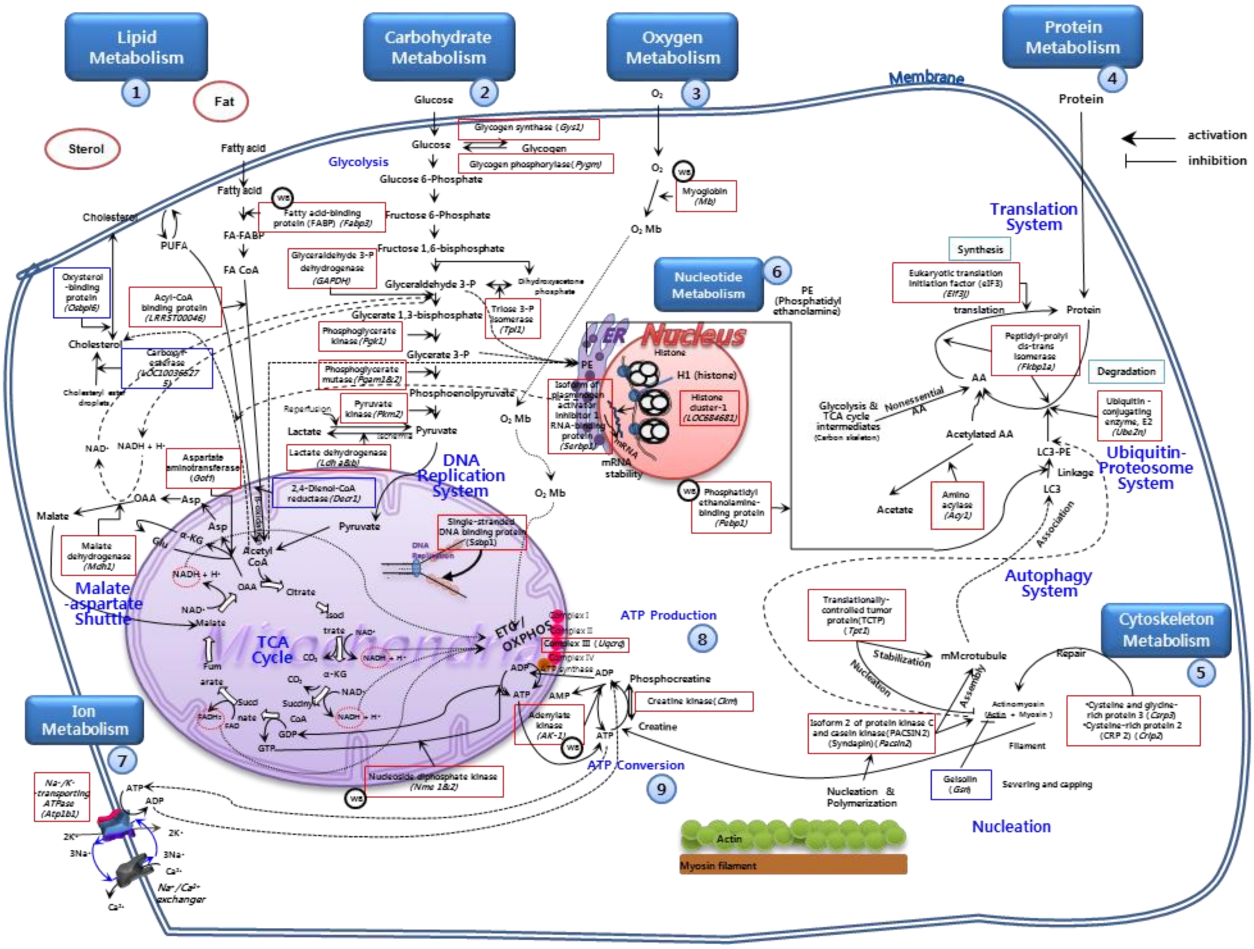

Supplementary Figure S5. Metabolic networks modulated by the WE treatment

WE intake also modulates lipid, carbohydrate, oxygen, protein, cytoskeleton, nucleotide, and ion metabolism (pathways 1-7). Of these, fatty acid catabolism was enhanced by the WE treatment through β-oxidation (pathway 1), glucose catabolism mediated through glycolysis and the malate-aspartate shuttle (pathway 2) and oxygen reserve catabolism mediated through myoglobin (pathway 3). These processes all lead to the increased production of ATP through the TCA cycle and oxidative phosphorylation (pathway 8). WE treatment also enhances the buffering capacity of ATP through ATP conversion (pathway 9). Furthermore, WE treatment maintains protein homeostasis through increased protein synthesis and degradation (pathway 4) and supports the cytoskeleton through the increased assembly and disassembly of actin filaments and microtubules (pathway 5). See the legend of Figure 3 for additional details. AA, amino acids; Asp, aspartate; ETC/OXPHOS, electron transport chain/oxidative phosphorylation; FA CoA; fatty acyl CoA; Glu, glutamate; α-KG, α-ketoglutarate; OAA, oxaloacetate.

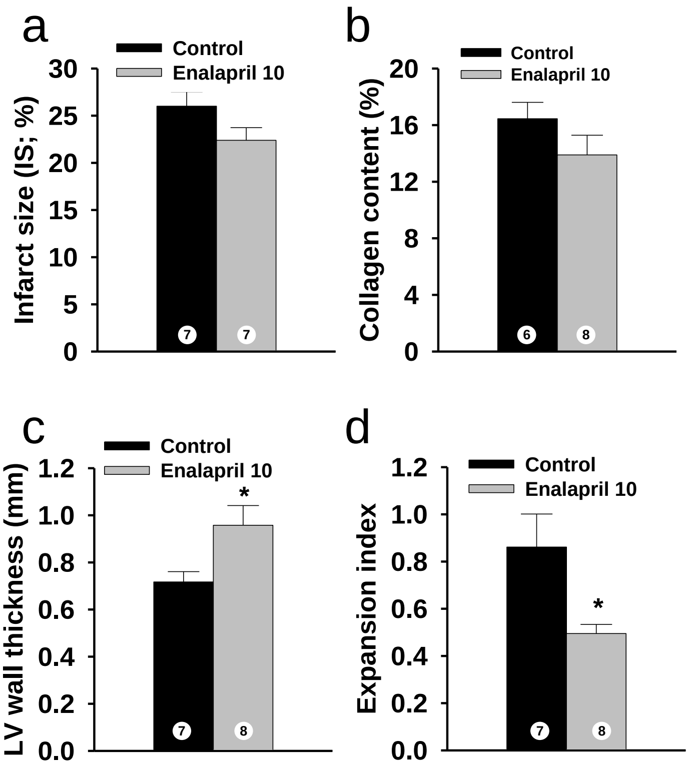

Supplementary Figure S6. The effect of enalapril supplementation in rat LI models

Enalapril, an angiotensin-converting enzyme inhibitor used in the treatment of chronic heart failure, was supplemented with 10 mg/kg/day for 7 days in the LI models. The numbers inside bars indicate the number of animals per group. $^{*}P <$ 0.05 compared to controls.

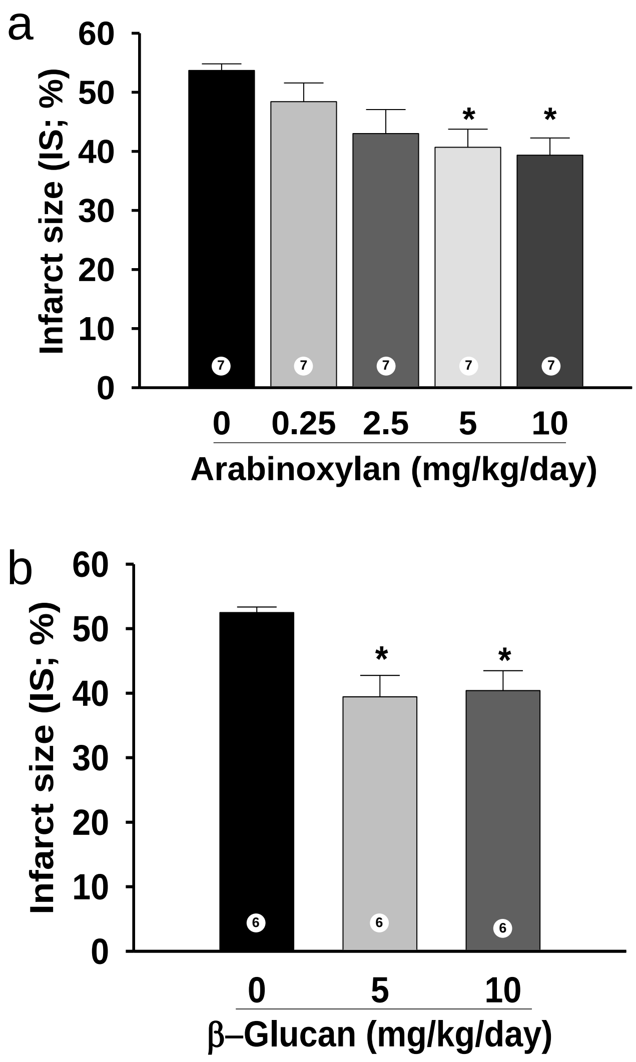

Supplementary Figure S7. Effect of varying dosages of arabinoxylan and β-glucan on infarct size in the SISR model

(**a**) Infarct size corresponding to various arabinoxylan dosages. (**b**) Infarct size corresponding to two β-glucan dosages. The range of the dosages was calculated based on the assumption that both water-soluble arabinoxylan (1.8% of the wheat grain) and β-glucan (1.0% of the wheat grain)[11] account for up to 2% of the 400 mg/kg/day WE dosage, which is equivalent to 8 mg/kg/day arabinoxylan and β-glucan. The numbers inside bars indicate the number of animals per group. $^{*}P < 0.05$ compared to controls.

a

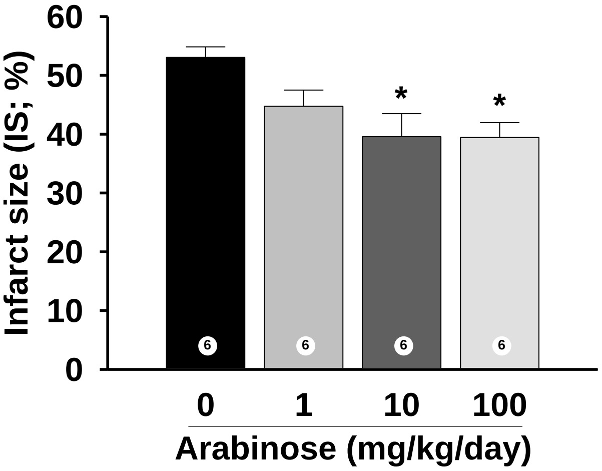

b

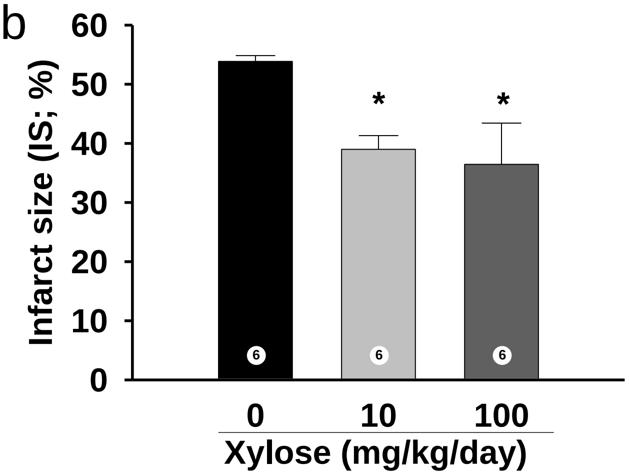

Supplementary Figure S8. Effect of varying dosages of arabinose and xylose on infarct size in the SISR model

(**a**) Infarct size after the supplementation of various arabinose dosages. (**b**) Infarct size after the supplementation of two different xylose dosages. The range of the dosages was calculated based on the assumption that arabinoxylan consists of only arabinose or xylose in the maximal dosage used in Supplementary Figure S7 (10 mg/kg/day), which is equivalent to 10 mg/kg/day. Note that dosages over 10 mg/kg/day show efficacy. The numbers inside bars indicate the number of animals per group. $^{*}P < 0.05$ compared to controls.

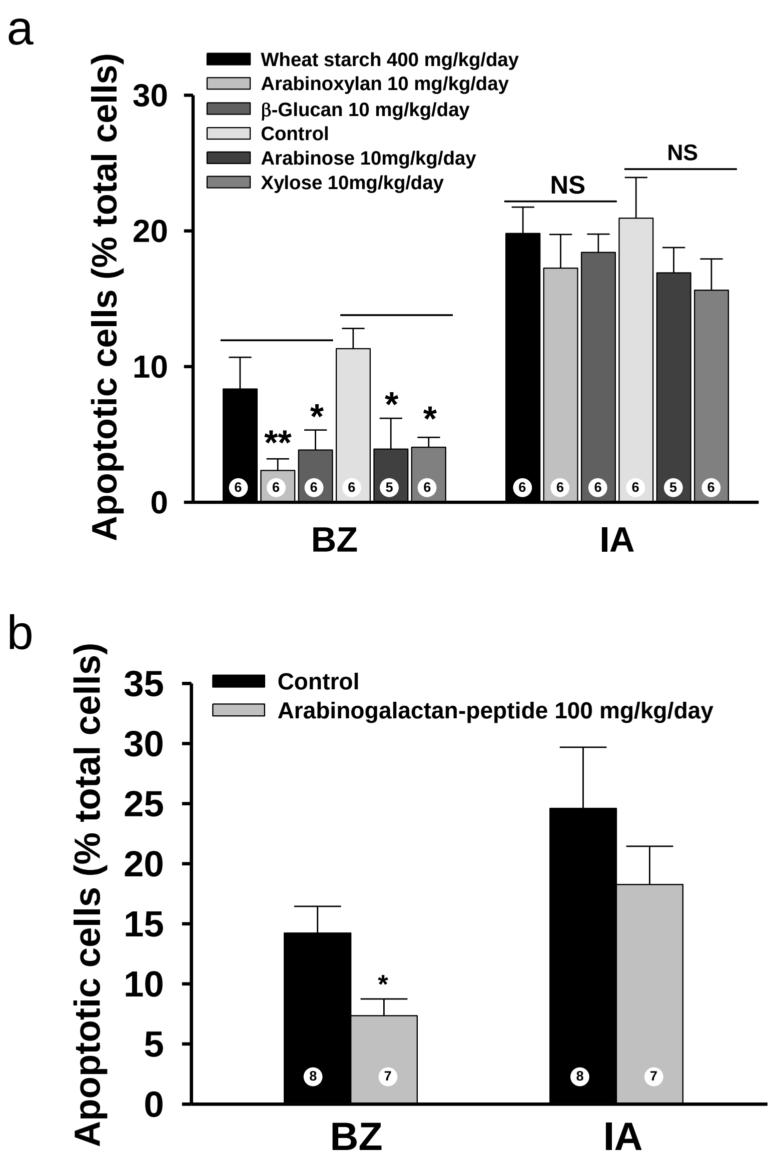

Supplementary Figure S9. Effect of various wheat components on apoptosis in the SISR model

(**a, b**) Ratio of apoptotic cells to total cells in border zone (BZ) and infarct area (IA) for arabinoxylan, β-glucan, arabinose, xylose (**a**) and arabinoglactan-peptide (**b**). Data divided into polysaccharide and monosaccharide groups are shown as horizontal lines. NS indicates no significance by statistical analysis. The numbers inside bars indicate the number of animals per group. $^{*}P < 0.05$, $^{**}P < 0.01$ compared to controls.

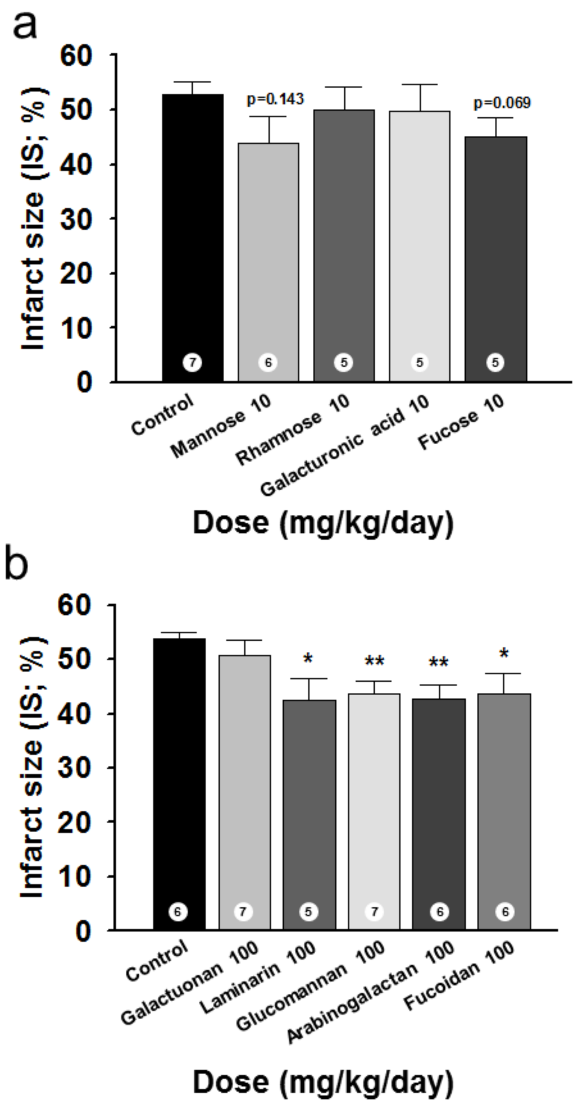

Supplementary Figure S10. Effects of various cell wall mono- and polysaccharides enriched in plant-based foods on infarct size in the SISR model

(**a**) Infarct size for various cell wall monosaccharides. (**b**) Infarct size for various cell wall polysaccharides. The numbers inside bars indicate the number of animals per group. $^{*}P < 0.05$, $^{**}P < 0.01$ compared with the controls.

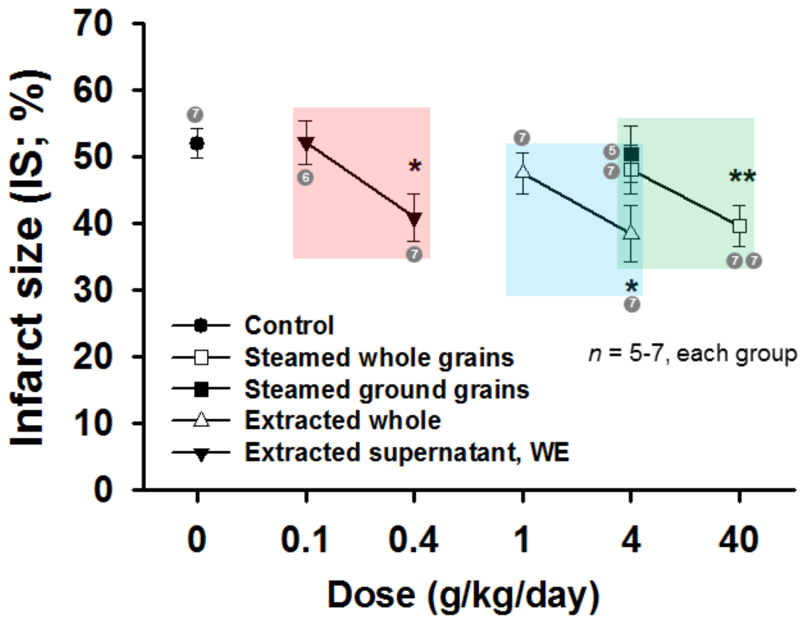

Supplementary Figure S11. Effect of wheat grain processing on infarct size
We chose wheat as a model food component and processed the wheat grains using the following methods (see *Materials and Methods* for more details): whole grains were steam cooked (Steamed whole grains), steam cooked and ground (Steamed ground grains), or ground and extracted with hot water before freeze-drying. In the last case, the grains were either extracted as a whole (Extracted whole) or were centrifuged to obtain a supernatant (Extracted supernatant, i.e., WE). The intake of steamed grains (whole and ground), extracted whole, and WE demonstrated efficacy at dosages of 40, 4, and 0.4 g/kg/day, respectively, indicating that the method of extraction via hot water and the concentration of the active polysaccharides are crucial for reducing the effective dosage through the increased availability of polysaccharides via the acid in the stomach and microorganisms in the large intestine. The range of dosages tested for each processing is highlighted as a color rectangular box. The numbers inside a graph indicate the number of animals per group. $^{*}P < 0.05$, $^{**}P < 0.01$ compared to controls.

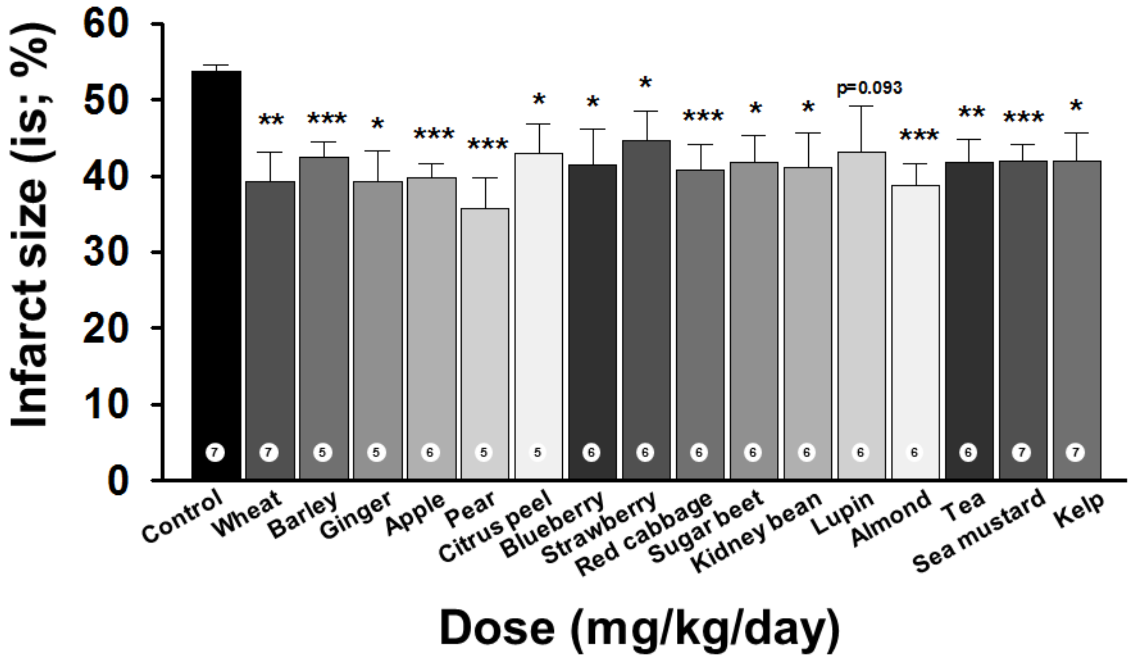

Supplementary Figure S12. Effects of various foods components on infarct size in the SISR model

Infarct size for various food components that compose plant-based diets at a dosage of 400 mg/kg/day each. The numbers inside bars indicate the number of animals per group. $^{*}P < 0.05$, $^{**}P < 0.01$, $^{***}P < 0.001$ compared with the controls.

Supplementary Tables

Table S1. Proteins exhibiting significant quantitative differences in the comparison of both the sham and control.

Table S1-E1

PLINE	DESCRIPTION	PEP_NUM	Log2_Ratio(avg.Sham/avg.Control)
**IPI00476246.4**	Tax_Id=10116 Gene_Symbol=Igh-6 Igh-6 protein	4	-2.34
**IPI00200102.1**	Tax_Id=10116 Gene_Symbol=Apoc1 Apolipoprotein C-I	3	-2.33
**IPI00778265.1**	Tax_Id=10116 Gene_Symbol=Cfh Uncharacterized protein	13	-2.29
**IPI00206780.1**	Tax_Id=10116 Gene_Symbol=Plg Plasminogen	40	-2.15
**IPI00210120.2**	Tax_Id=10116 Gene_Symbol=Vtn Uncharacterized protein	15	-2.06
**IPI00188225.1**	Tax_Id=10116 Gene_Symbol=Crp C-reactive protein	80	-1.95
**IPI00778633.1**	Tax_Id=10116 Gene_Symbol=Apoh Uncharacterized protein	28	-1.90
**IPI00210340.3**	Tax_Id=10116 Gene_Symbol=Serpina10 Protein Z-dependent protease inhibitor	2	-1.84
**IPI00876581.1**	Tax_Id=10116 Gene_Symbol=Rbp4 Retinol-binding protein 4	6	-1.84
**IPI00205389.6**	Tax_Id=10116 Gene_Symbol=Fgb Isoform 1 of Fibrinogen beta chain	41	-1.82
**IPI00327745.1**	Tax_Id=10116 Gene_Symbol=Apcs Serum amyloid P-component	2	-1.78
**IPI00188541.3**	Tax_Id=10116 Gene_Symbol=Itih4 inter-alpha-inhibitor H4 heavy chain	36	-1.74
**IPI00212666.2**	Tax_Id=10116 Gene_Symbol=Mug1;LOC297568 Isoform 1 of Murinoglobulin-1	212	-1.71
**IPI00201262.1**	Tax_Id=10116 Gene_Symbol=Mug1;LOC297568 Alpha-1-inhibitor 3	226	-1.70
**IPI00368704.3**	Tax_Id=10116 Gene_Symbol=Cpamd8 similar to Murinoglobulin 1 homolog	162	-1.70
**IPI00231370.7**	Tax_Id=10116 Gene_Symbol=S100a8 Protein S100-A8	4	-1.69
**IPI00191737.6**	Tax_Id=10116 Gene_Symbol=Alb Serum albumin	491	-1.69
**IPI00564327.3**	Tax_Id=10116 Gene_Symbol=Mug2 Uncharacterized protein	139	-1.68
**IPI00957456.1**	Tax_Id=10116 Gene_Symbol=LOC100365275 microsomal carboxyesterase E1-like	16	-1.68
**IPI00231262.7**	Tax_Id=10116 Gene_Symbol=S100a9 Protein S100-A9	9	-1.67
**IPI00923716.1**	Tax_Id=10116 Gene_Symbol=Gsn Isoform 2 of Gelsolin	34	-1.65
**IPI00199695.3**	Tax_Id=10116 Gene_Symbol=Serpinf2 Uncharacterized protein	3	-1.63
**IPI00957813.1**	Tax_Id=10116 Gene_Symbol=LOC684726 carboxypeptidase B2-like	2	-1.63
**IPI00949032.1**	Tax_Id=10116 Gene_Symbol=Itih3 Uncharacterized protein	14	-1.62
**IPI00567916.3**	Tax_Id=10116 Gene_Symbol=Fetub Fetuin-B	9	-1.62
**IPI00382317.1**	Tax_Id=10116 Gene_Symbol=Fga Uncharacterized protein	29	-1.61
**IPI00480639.3**	Tax_Id=10116 Gene_Symbol=C3 Complement C3 (Fragment)	158	-1.58
**IPI00206786.3**	Tax_Id=10116 Gene_Symbol=F10 Coagulation factor X	2	-1.57
**IPI00324019.1**	Tax_Id=10116 Gene_Symbol=Serpina1 Alpha-1-antiproteinase	82	-1.56
**IPI00230944.2**	Tax_Id=10116 Gene_Symbol=Fgg Isoform Gamma-A of Fibrinogen gamma chain	23	-1.50
**IPI00211075.2**	Tax_Id=10116 Gene_Symbol=Serpina3n Serpina3n-like protein	15	-1.49
**IPI00422011.2**	Tax_Id=10116 Gene_Symbol=Cfb complement factor B	3	-1.49
**IPI00197703.2**	Tax_Id=10116 Gene_Symbol=Apoa1 Apolipoprotein A-I	37	-1.47
**IPI00200593.1**	Tax_Id=10116 Gene_Symbol=Serpina3k Serine protease inhibitor A3K	81	-1.46
**IPI00326140.3**	Tax_Id=10116 Gene_Symbol=Pzp Alpha-1-macroglobulin	88	-1.43
**IPI00327469.1**	Tax_Id=10116 Gene_Symbol=Ahsg Alpha-2-HS-glycoprotein	21	-1.40
**IPI00231423.6**	Tax_Id=10116 Gene_Symbol=C9 Uncharacterized protein	9	-1.37
**IPI00679202.2**	Tax_Id=10116 Gene_Symbol=Tf Isoform 1 of Serotransferrin	123	-1.36
**IPI00188967.1**	Tax_Id=10116 Gene_Symbol=LOC500183 Ig kappa chain C region, A allele	6	-1.35
**IPI00207668.1**	Tax_Id=10116 Gene_Symbol=Afm Afamin	17	-1.31
**IPI00204451.1**	Tax_Id=10116 Gene_Symbol=Cfi Complement factor I	7	-1.31
**IPI00190701.5**	Tax_Id=10116 Gene_Symbol=Apoe Apolipoprotein E	16	-1.31

**IPI00950336.1**	Tax_Id=10116 Gene_Symbol=Cp Uncharacterized protein	22	-1.30
**IPI00200591.1**	Tax_Id=10116 Gene_Symbol=LOC299282 Serine protease inhibitor A3L	83	-1.28
**IPI00198667.7**	Tax_Id=10116 Gene_Symbol=Clu Clusterin	12	-1.27
**IPI00958555.1**	Tax_Id=10116 Gene_Symbol=- Uncharacterized protein	2	-1.26
**IPI00392216.1**	Tax_Id=10116 Gene_Symbol=LOC299282 Liver regeneration protein lrryan	76	-1.23
**IPI00195516.6**	Tax_Id=10116 Gene_Symbol=Hpx Hemopexin	70	-1.22
**IPI00194097.5**	Tax_Id=10116 Gene_Symbol=Gc Vitamin D-binding protein	22	-1.21
**IPI00209744.1**	Tax_Id=10116 Gene_Symbol=Agt Angiotensinogen	4	-1.20
**IPI00189981.1**	Tax_Id=10116 Gene_Symbol=F2 Prothrombin (Fragment)	21	-1.19
**IPI00324380.3**	Tax_Id=10116 Gene_Symbol=Ttr Transthyretin	19	-1.17
**IPI00231264.7**	Tax_Id=10116 Gene_Symbol=Pon1 Serum paraoxonase/arylesterase 1	4	-1.11
**IPI00231982.1**	Tax_Id=10116 Gene_Symbol=Fn1 Isoform 2 of Fibronectin	17	-1.09
**IPI00368608.2**	Tax_Id=10116 Gene_Symbol=IgG-2a IgG-2a protein	17	-1.05
**IPI00557598.3**	Tax_Id=10116 Gene_Symbol=LOC100363606 Uncharacterized protein (Fragment)	12	-1.04
**IPI00191715.1**	Tax_Id=10116 Gene_Symbol=Orm1 Alpha-1-acid glycoprotein	13	-1.02
**IPI01016420.1**	Tax_Id=10116 Gene_Symbol=Serpina3m Serine protease inhibitor A3M (Fragment)	8	-1.01
**IPI00471828.1**	Tax_Id=10116 Gene_Symbol=Map3k11 Mitogen-activated protein kinase kinase kinase 11	2	-0.99
**IPI00372792.1**	Tax_Id=10116 Gene_Symbol=Serping1 Plasma protease C1 inhibitor	4	-0.98
**IPI00778978.2**	Tax_Id=10116 Gene_Symbol=Slc4a1 Uncharacterized protein	9	-0.98
**IPI00389609.4**	Tax_Id=10116 Gene_Symbol=Cybb Uncharacterized protein	3	-0.95
**IPI00324272.1**	Tax_Id=10116 Gene_Symbol=Apoa4 Apolipoprotein A-IV	13	-0.95
**IPI00421355.1**	Tax_Id=10116 Gene_Symbol=- LRRGT00057	4	-0.93
**IPI00231282.1**	Tax_Id=10116 Gene_Symbol=Kng2;Kng1 Isoform LMW of Kininogen-1	3	-0.89
**IPI00205568.1**	Tax_Id=10116 Gene_Symbol=Serpina4 Kallistatin	7	-0.88
**IPI00769165.1**	Tax_Id=10116 Gene_Symbol=LOC681544 histidine-rich glycoprotein-like	4	-0.88
**IPI00777179.1**	Tax_Id=10116 Gene_Symbol=Anxa1 Uncharacterized protein	9	-0.84
**IPI00565708.2**	Tax_Id=10116 Gene_Symbol=Hp Isoform 1 of Haptoglobin	5	-0.83
**IPI00948338.1**	Tax_Id=10116 Gene_Symbol=C4b complement C4 precursor	12	-0.82
**IPI00201333.5**	Tax_Id=10116 Gene_Symbol=Ganab Uncharacterized protein	2	-0.82
**IPI00561922.5**	Tax_Id=10116 Gene_Symbol=Hrg Uncharacterized protein	4	-0.81
**IPI00679245.2**	Tax_Id=10116 Gene_Symbol=Kng1l1 T-kininogen 2	6	-0.80
**IPI00210900.1**	Tax_Id=10116 Gene_Symbol=Ambp Protein AMBP	9	-0.78
**IPI00991200.1**	Tax_Id=10116 Gene_Symbol=Ryr2 Islet-type ryanodine receptor	32	-0.76
**IPI00209973.2**	Tax_Id=10116 Gene_Symbol=C4bpa Uncharacterized protein	2	-0.74
**IPI00950891.1**	Tax_Id=10116 Gene_Symbol=Pdzd8 Uncharacterized protein	2	-0.64
**IPI00765471.2**	Tax_Id=10116 Gene_Symbol=Rabgap1l Uncharacterized protein	2	-0.64
**IPI00960040.1**	Tax_Id=10116 Gene_Symbol=LOC680097 histone cluster 1, H2ae-like	26	-0.63
**IPI00201057.5**	Tax_Id=10116 Gene_Symbol=Hk2 Hexokinase-2	5	-0.62
**IPI00358463.1**	Tax_Id=10116 Gene_Symbol=Arhgdib Uncharacterized protein	2	-0.61
**IPI00362791.3**	Tax_Id=10116 Gene_Symbol=Ubqln2 Uncharacterized protein	2	-0.61
**IPI00951116.1**	Tax_Id=10116 Gene_Symbol=Hbb 16 kDa protein	632	-0.61
**IPI00231192.5**	Tax_Id=10116 Gene_Symbol=LOC689064;LOC100134871;Hbb Hemoglobin subunit beta-2	499	-0.60
**IPI00230897.5**	Tax_Id=10116 Gene_Symbol=Hbb Hemoglobin subunit beta-1	641	-0.60
**IPI00231677.5**	Tax_Id=10116 Gene_Symbol=Ywhah 14-3-3 protein eta	13	0.60
**IPI00207675.4**	Tax_Id=10116 Gene_Symbol=Zeb2 Zfhx1b zinc finger homeobox 1b	3	0.60
**IPI00959144.1**	Tax_Id=10116 Gene_Symbol=LOC363331 plasma membrane associated protein, S3-12	7	0.60
**IPI00464791.5**	Tax_Id=10116 Gene_Symbol=Acy1 Aminoacylase-1A	2	0.60
**IPI00559898.2**	Tax_Id=10116 Gene_Symbol=RGD1564688 Glyceraldehyde-3-phosphate dehydrogenase	41	0.60
**IPI00393489.2**	Tax_Id=10116 Gene_Symbol=Sorbs2 Uncharacterized protein	11	0.61

**IPI00364189.2**	Tax_Id=10116 Gene_Symbol=Eif3j Eukaryotic translation initiation factor 3 subunit J	3	0.62
**IPI00209938.1**	Tax_Id=10116 Gene_Symbol=Nexn Isoform 1 of Nexilin	7	0.62
**IPI00209789.1**	Tax_Id=10116 Gene_Symbol=Fkbp3 peptidyl-prolyl cis-trans isomerase FKBP3	2	0.63
**IPI00767147.1**	Tax_Id=10116 Gene_Symbol=Eno1-ps1 enolase 3	101	0.63
**IPI00950965.1**	Tax_Id=10116 Gene_Symbol=Aprt Adenine phosphoribosyltransferase	10	0.63
**IPI00948742.1**	Tax_Id=10116 Gene_Symbol=- Uncharacterized protein	2	0.64
**IPI00882501.1**	Tax_Id=10116 Gene_Symbol=Ncl Nucleolin	3	0.64
**IPI00231434.6**	Tax_Id=10116 Gene_Symbol=Fkbp1a Peptidyl-prolyl cis-trans isomerase FKBP1A	4	0.64
**IPI00568369.3**	Tax_Id=10116 Gene_Symbol=Dstn Destrin	7	0.65
**IPI00203352.1**	Tax_Id=10116 Gene_Symbol=Bzw2 Basic leucine zipper and W2 domain-containing protein 2	2	0.65
**IPI00364474.1**	Tax_Id=10116 Gene_Symbol=Itpa Uncharacterized protein	2	0.65
**IPI00372839.3**	Tax_Id=10116 Gene_Symbol=Col6a2 collagen, type VI, alpha 2	3	0.65
**IPI00781155.1**	Tax_Id=10116 Gene_Symbol=Akr1cl1 Uncharacterized protein	7	0.66
**IPI00656375.3**	Tax_Id=10116 Gene_Symbol=RGD1562758 glyceraldehyde-3-phosphate dehydrogenase-like	45	0.66
**IPI00361208.4**	Tax_Id=10116 Gene_Symbol=Trim72 Tripartite motif-containing protein 72	20	0.67
**IPI00915560.1**	Tax_Id=10116 Gene_Symbol=Ppa1 inorganic pyrophosphatase	6	0.68
**IPI00214477.1**	Tax_Id=10116 Gene_Symbol=Hspb3 Heat shock protein beta-3	2	0.68
**IPI00231148.8**	Tax_Id=10116 Gene_Symbol=Gpd1 Glycerol-3-phosphate dehydrogenase [NAD+], cytoplasmic	9	0.68
**IPI00190179.3**	Tax_Id=10116 Gene_Symbol=Pygl Glycogen phosphorylase, liver form	17	0.69
**IPI00209848.1**	Tax_Id=10116 Gene_Symbol=Cstb Cystatin-B	3	0.70
**IPI00231609.5**	Tax_Id=10116 Gene_Symbol=- 60S ribosomal protein L6	4	0.70
**IPI00213807.2**	Tax_Id=10116 Gene_Symbol=LOC685718;LOC683302 tumor protein, translationally-controlled 1-like	6	0.71
**IPI00371853.4**	Tax_Id=10116 Gene_Symbol=Col6a1 collagen, type VI, alpha 1	19	0.71
**IPI00949622.1**	Tax_Id=10116 Gene_Symbol=Ptgr2 Uncharacterized protein	14	0.73
**IPI00197711.1**	Tax_Id=10116 Gene_Symbol=Ldha L-lactate dehydrogenase A chain	55	0.74
**IPI00781133.1**	Tax_Id=10116 Gene_Symbol=Aco1 Uncharacterized protein	2	0.74
**IPI00470288.4**	Tax_Id=10116 Gene_Symbol=Ckb Creatine kinase B-type	62	0.74
**IPI00359074.1**	Tax_Id=10116 Gene_Symbol=Ciapin1 Anamorsin	7	0.74
**IPI00949068.1**	Tax_Id=10116 Gene_Symbol=Pgm1 Uncharacterized protein	8	0.74
**IPI00948869.1**	Tax_Id=10116 Gene_Symbol=Pygb glycogen phosphorylase, brain form	46	0.75
**IPI00231929.7**	Tax_Id=10116 Gene_Symbol=Pkm2 Isoform M1 of Pyruvate kinase isozymes M1/M2	75	0.75
**IPI00231506.5**	Tax_Id=10116 Gene_Symbol=Pgam2 Phosphoglycerate mutase 2	28	0.76
**IPI00231963.5**	Tax_Id=10116 Gene_Symbol=Ddt D-dopachrome decarboxylase	10	0.76
**IPI00368053.2**	Tax_Id=10116 Gene_Symbol=Glod4 Glyoxalase domain-containing protein 4	2	0.76
**IPI00362395.5**	Tax_Id=10116 Gene_Symbol=Esd S-formylglutathione hydrolase	4	0.76
**IPI00198497.1**	Tax_Id=10116 Gene_Symbol=Csrp3 Cysteine and glycine-rich protein 3	18	0.77
**IPI00370752.3**	Tax_Id=10116 Gene_Symbol=Nit2 Omega-amidase NIT2	4	0.77
**IPI00205601.1**	Tax_Id=10116 Gene_Symbol=Smpx Small muscular protein	4	0.78
**IPI00364311.1**	Tax_Id=10116 Gene_Symbol=Gpi Glucose-6-phosphate isomerase	34	0.78
**IPI00203443.3**	Tax_Id=10116 Gene_Symbol=Ptgr1 Prostaglandin reductase 1	9	0.78
**IPI00895591.1**	Tax_Id=10116 Gene_Symbol=Pygm glycogen phosphorylase, muscle form	79	0.78
**IPI00968497.1**	Tax_Id=10116 Gene_Symbol=Acyp2-ps1 Acylphosphatase-2	2	0.79
**IPI00781084.2**	Tax_Id=10116 Gene_Symbol=Ppp1r12b Uncharacterized protein	2	0.79
**IPI00211053.6**	Tax_Id=10116 Gene_Symbol=Ckm Creatine kinase M-type	250	0.80
**IPI00231737.5**	Tax_Id=10116 Gene_Symbol=Akr1b1 Aldose reductase	20	0.81
**IPI00957862.1**	Tax_Id=10116 Gene_Symbol=LOC100362999 hsc70-interacting protein-like	5	0.81
**IPI00231770.5**	Tax_Id=10116 Gene_Symbol=Prkar1a cAMP-dependent protein kinase type I-alpha regulatory subunit	3	0.82
**IPI00421428.9**	Tax_Id=10116 Gene_Symbol=Pgam1 Phosphoglycerate mutase 1	17	0.83
**IPI00212523.1**	Tax_Id=10116 Gene_Symbol=Park7 Protein DJ-1	41	0.84

**IPI00230902.5**	Tax_Id=10116 Gene_Symbol=Gyg1 Glycogenin-1	5	0.84
**IPI00421434.3**	Tax_Id=10116 Gene_Symbol=Sdhd Uncharacterized protein	2	0.86
**IPI00562366.3**	Tax_Id=10116 Gene_Symbol=- Uncharacterized protein (Fragment)	4	0.87
**IPI00231783.5**	Tax_Id=10116 Gene_Symbol=Ldhb L-lactate dehydrogenase B chain	201	0.88
**IPI00231767.5**	Tax_Id=10116 Gene_Symbol=Tpi1 Triosephosphate isomerase	64	0.90
**IPI00951939.1**	Tax_Id=10116 Gene_Symbol=Srsf2 Uncharacterized protein	2	0.93
**IPI00421513.8**	Tax_Id=10116 Gene_Symbol=Got1 Aspartate aminotransferase, cytoplasmic	62	0.93
**IPI00194404.5**	Tax_Id=10116 Gene_Symbol=Nme1 Nucleoside diphosphate kinase A	13	0.93
**IPI00208306.1**	Tax_Id=10116 Gene_Symbol=Tpt1 Translationally-controlled tumor protein	13	0.94
**IPI00368005.2**	Tax_Id=10116 Gene_Symbol=Irf2bpl Interferon regulatory factor 2-binding protein-like	2	0.94
**IPI00188304.3**	Tax_Id=10116 Gene_Symbol=Glo1 Lactoylglutathione lyase	11	0.95
**IPI00231260.5**	Tax_Id=10116 Gene_Symbol=Prdx6 Peroxiredoxin-6	48	0.96
**IPI00231734.5**	Tax_Id=10116 Gene_Symbol=Aldoa Fructose-bisphosphate aldolase A	145	0.96
**IPI00231426.6**	Tax_Id=10116 Gene_Symbol=Pgk1 Phosphoglycerate kinase 1	53	0.98
**IPI00325189.4**	Tax_Id=10116 Gene_Symbol=Nme2 Nucleoside diphosphate kinase B	43	0.98
**IPI00951813.1**	Tax_Id=10116 Gene_Symbol=Ugp2 Uncharacterized protein (Fragment)	9	1.01
**IPI00214052.1**	Tax_Id=10116 Gene_Symbol=Cmbl Carboxymethylenebutenolidase homolog	6	1.02
**IPI00198717.8**	Tax_Id=10116 Gene_Symbol=Mdh1 Malate dehydrogenase, cytoplasmic	73	1.03
**IPI00230937.5**	Tax_Id=10116 Gene_Symbol=Pebp1 Phosphatidylethanolamine-binding protein 1	29	1.08
**IPI00210351.2**	Tax_Id=10116 Gene_Symbol=Ak1 Adenylate kinase isoenzyme 1	23	1.09
**IPI00231069.5**	Tax_Id=10116 Gene_Symbol=LOC100365425;Dbi Acyl-CoA-binding protein	7	1.10
**IPI00231650.7**	Tax_Id=10116 Gene_Symbol=Hist1h1d Histone H1.2	5	1.10
**IPI00958032.1**	Tax_Id=10116 Gene_Symbol=LOC100363145 rCG42396-like isoform 2	2	1.10
**IPI00191897.4**	Tax_Id=10116 Gene_Symbol=- Nucleoside diphosphate kinase	10	1.11
**IPI00411230.3**	Tax_Id=10116 Gene_Symbol=Gstm2 Glutathione S-transferase Mu 2	17	1.15
**IPI00210090.3**	Tax_Id=10116 Gene_Symbol=Hnrnpu SP120	2	1.20
**IPI00193108.1**	Tax_Id=10116 Gene_Symbol=Pycard Apoptosis-associated speck-like protein	2	1.25
**IPI00231971.5**	Tax_Id=10116 Gene_Symbol=Fabp3 Fatty acid-binding protein, heart	523	1.49
**IPI00214517.3**	Tax_Id=10116 Gene_Symbol=Mb Myoglobin	562	1.64

Table S1-E2

IPI NO.	DESCRIPTION	PEP_NUM	Log2_Ratio(avg.Sham/avg.Control)
**IPI00188225.1**	Tax=10116 GS=Crp C-reactive protein	45	-2.98
**IPI00210120.2**	Tax=10116 GS=Vtn Uncharacterized protein	17	-2.51
**IPI00778633.1**	Tax=10116 GS=Apoh Uncharacterized protein	12	-2.24
**IPI00189981.1**	Tax=10116 GS=F2 Prothrombin (Fragment)	19	-2.17
**IPI00206780.1**	Tax=10116 GS=Plg Plasminogen	25	-2.15
**IPI00231423.6**	Tax=10116 GS=C9 Uncharacterized protein	3	-2.04
**IPI00949032.1**	Tax=10116 GS=Itih3 Uncharacterized protein	7	-2.02
**IPI00923716.1**	Tax=10116 GS=Gsn Isoform 2 of Gelsolin	37	-1.96
**IPI00207668.1**	Tax=10116 GS=Afm Afamin	18	-1.84
**IPI00230944.2**	Tax=10116 GS=Fgg Isoform Gamma-A of Fibrinogen gamma chain	21	-1.80
**IPI00480639.3**	Tax=10116 GS=C3 Complement C3 (Fragment)	116	-1.78
**IPI00188541.3**	Tax=10116 GS=Itih4 inter-alpha-inhibitor H4 heavy chain	28	-1.75
**IPI00561922.5**	Tax=10116 GS=Hrg Uncharacterized protein	6	-1.72
**IPI00191737.6**	Tax=10116 GS=Alb Serum albumin	574	-1.72
**IPI00197703.2**	Tax=10116 GS=Apoa1 Apolipoprotein A-I	42	-1.71
**IPI00780167.2**	Tax=10116 GS=Mug1;LOC297568 Uncharacterized protein	135	-1.70
**IPI00368704.3**	Tax=10116 GS=Cpamd8 similar to Murinoglobulin 1 homolog	102	-1.69

**IPI00190701.5**	Tax=10116 GS=Apoe Apolipoprotein E	13	-1.68
**IPI00564327.3**	Tax=10116 GS=Mug2 Uncharacterized protein	129	-1.64
**IPI00194097.5**	Tax=10116 GS=Gc Vitamin D-binding protein	14	-1.62
**IPI00382317.1**	Tax=10116 GS=Fga Uncharacterized protein	40	-1.62
**IPI00201262.1**	Tax=10116 GS=Mug1;LOC297568 Alpha-1-inhibitor 3	180	-1.62
**IPI00326140.3**	Tax=10116 GS=Pzp Alpha-1-macroglobulin	65	-1.60
**IPI00957456.1**	Tax=10116 GS=LOC100365275 microsomal carboxyesterase E1-like	18	-1.59
**IPI00200591.1**	Tax=10116 GS=LOC299282 Serine protease inhibitor A3L	90	-1.55
**IPI00324019.1**	Tax=10116 GS=Serpina1 Alpha-1-antiproteinase	88	-1.53
**IPI00948614.1**	Tax=10116 GS=Fgb Uncharacterized protein	30	-1.53
**IPI00324272.1**	Tax=10116 GS=Apoa4 Apolipoprotein A-IV	9	-1.53
**IPI00200593.1**	Tax=10116 GS=Serpina3k Serine protease inhibitor A3K	78	-1.51
**IPI00392216.1**	Tax=10116 GS=LOC299282 Liver regeneration protein lrryan	84	-1.51
**IPI00198667.7**	Tax=10116 GS=Clu Clusterin	11	-1.50
**IPI00876581.1**	Tax=10116 GS=Rbp4 Retinol-binding protein 4	10	-1.49
**IPI00948338.1**	Tax=10116 GS=C4b complement C4 precursor	4	-1.41
**IPI00567916.3**	Tax=10116 GS=Fetub Fetuin-B	11	-1.40
**IPI00327469.1**	Tax=10116 GS=Ahsg Alpha-2-HS-glycoprotein	43	-1.40
**IPI00679202.2**	Tax=10116 GS=Tf Isoform 1 of Serotransferrin	169	-1.38
**IPI00655254.3**	Tax=10116 GS=RGD1310507 Uncharacterized protein	2	-1.33
**IPI00476292.4**	Tax=10116 GS=Cp Ceruloplasmin	21	-1.32
**IPI00958555.1**	Tax=10116 GS=- Uncharacterized protein	2	-1.27
**IPI00957985.1**	Tax=10116 GS=Fn1 Uncharacterized protein	9	-1.27
**IPI00679245.2**	Tax=10116 GS=Kng1l1 T-kininogen 2	5	-1.26
**IPI00372372.2**	Tax=10116 GS=Serpinc1 Uncharacterized protein	8	-1.24
**IPI01016420.1**	Tax=10116 GS=Serpina3m Serine protease inhibitor A3M (Fragment)	12	-1.24
**IPI00422011.2**	Tax=10116 GS=Cfb complement factor B	6	-1.21
**IPI00231264.7**	Tax=10116 GS=Pon1 Serum paraoxonase/arylesterase 1	3	-1.21
**IPI00782787.1**	Tax=10116 GS=LOC362795 Ig gamma-2C chain C region	2	-1.13
**IPI00195516.6**	Tax=10116 GS=Hpx Hemopexin	69	-1.11
**IPI00565708.2**	Tax=10116 GS=Hp Isoform 1 of Haptoglobin	16	-1.09
**IPI00778265.1**	Tax=10116 GS=Cfh Uncharacterized protein	5	-1.08
**IPI00362791.3**	Tax=10116 GS=Ubqln2 Uncharacterized protein	2	-0.97
**IPI00886485.9**	Tax=10116 GS=Kng1 Rat T-kininogen	7	-0.97
**IPI00211075.2**	Tax=10116 GS=Serpina3n Serpina3n-like protein	7	-0.92
**IPI00210900.1**	Tax=10116 GS=Ambp Protein AMBP	6	-0.90
**IPI00339124.3**	Tax=10116 GS=Atp1b1 Sodium/potassium-transporting ATPase subunit beta-1	6	-0.83
**IPI00777179.1**	Tax=10116 GS=Anxa1 Uncharacterized protein	6	-0.80
**IPI00568389.2**	Tax=10116 GS=- LOC500183 protein	2	-0.74
**IPI00948792.1**	Tax=10116 GS=Maoa Uncharacterized protein	10	-0.74
**IPI00368608.2**	Tax=10116 GS=IgG-2a IgG-2a protein	10	-0.71
**IPI00779086.2**	Tax=10116 GS=- Uncharacterized protein (Fragment)	2	-0.68
**IPI00204451.1**	Tax=10116 GS=Cfi Complement factor I	5	-0.67
**IPI00387739.3**	Tax=10116 GS=- Uncharacterized protein (Fragment)	2	-0.65
**IPI00991200.1**	Tax=10116 GS=Ryr2 Islet-type ryanodine receptor	59	-0.63
**IPI00359098.3**	Tax=10116 GS=- Uncharacterized protein	3	-0.60
**IPI00470317.5**	Tax=10116 GS=Eef1g Elongation factor 1-gamma	4	0.60
**IPI00656375.3**	Tax=10116 GS=RGD1562758 glyceraldehyde-3-phosphate dehydrogenase-like	77	0.60
**IPI00213013.1**	Tax=10116 GS=Stip1 Stress-induced-phosphoprotein 1	6	0.61
**IPI00895591.1**	Tax=10116 GS=Pygm glycogen phosphorylase, muscle form	130	0.61

**IPI00765535.1**	Tax=10116 GS=Hmcn1 hemicentin 1	3	0.61
**IPI00951991.1**	Tax=10116 GS=Aldoa 45 kDa protein	156	0.61
**IPI00231275.7**	Tax=10116 GS=Lgals1 Galectin-1	34	0.61
**IPI00948869.1**	Tax=10116 GS=Pygb glycogen phosphorylase, brain form	76	0.61
**IPI00212258.3**	Tax=10116 GS=Phpt1 phosphohistidine phosphatase 1	5	0.62
**IPI00361815.3**	Tax=10116 GS=- Uncharacterized protein	167	0.63
**IPI00949068.1**	Tax=10116 GS=Pgm1 Uncharacterized protein	9	0.63
**IPI00208026.1**	Tax=10116 GS=Selenbp1 Selenium-binding protein 1	10	0.63
**IPI00231146.7**	Tax=10116 GS=Hint1;Hint1-ps1 Histidine triad nucleotide-binding protein 1	6	0.63
**IPI00396852.2**	Tax=10116 GS=Nexn Isoform 2 of Nexilin	9	0.64
**IPI00231929.7**	Tax=10116 GS=Pkm2 Isoform M1 of Pyruvate kinase isozymes M1/M2	88	0.64
**IPI00231737.5**	Tax=10116 GS=Akr1b1 Aldose reductase	25	0.64
**IPI00959144.1**	Tax=10116 GS=LOC363331 plasma membrane associated protein, S3-12	16	0.65
**IPI00421428.9**	Tax=10116 GS=Pgam1 Phosphoglycerate mutase 1	14	0.67
**IPI00365217.1**	Tax=10116 GS=Cmpk1 UMP-CMP kinase	4	0.68
**IPI00200352.1**	Tax=10116 GS=Crip2 Cysteine-rich protein 2	14	0.69
**IPI00190559.3**	Tax=10116 GS=Ube2n Ubiquitin-conjugating enzyme E2 N	2	0.69
**IPI00211053.6**	Tax=10116 GS=Ckm Creatine kinase M-type	383	0.70
**IPI00561262.3**	Tax=10116 GS=- Uncharacterized protein	19	0.71
**IPI00951911.1**	Tax=10116 GS=Hprt1 Uncharacterized protein	8	0.71
**IPI00197711.1**	Tax=10116 GS=Ldha L-lactate dehydrogenase A chain	56	0.72
**IPI00231783.5**	Tax=10116 GS=Ldhb L-lactate dehydrogenase B chain	187	0.72
**IPI00951813.1**	Tax=10116 GS=Ugp2 Uncharacterized protein (Fragment)	7	0.73
**IPI00231426.6**	Tax=10116 GS=Pgk1 Phosphoglycerate kinase 1	82	0.73
**IPI00231434.6**	Tax=10116 GS=Fkbp1a Peptidyl-prolyl cis-trans isomerase FKBP1A	2	0.74
**IPI00900498.1**	Tax=10116 GS=Ptgr2 Isoform 1 of Prostaglandin reductase 2	7	0.75
**IPI00421513.8**	Tax=10116 GS=Got1 Aspartate aminotransferase, cytoplasmic	82	0.75
**IPI00193233.1**	Tax=10116 GS=Cyb5b Cytochrome b5 type B	3	0.75
**IPI00194404.5**	Tax=10116 GS=Nme1 Nucleoside diphosphate kinase A	26	0.75
**IPI00208306.1**	Tax=10116 GS=Tpt1 Translationally-controlled tumor protein	18	0.75
**IPI00464791.5**	Tax=10116 GS=Acy1 Aminoacylase-1A	5	0.77
**IPI00231260.5**	Tax=10116 GS=Prdx6 Peroxiredoxin-6	26	0.79
**IPI00325189.4**	Tax=10116 GS=Nme2 Nucleoside diphosphate kinase B	48	0.80
**IPI00198717.8**	Tax=10116 GS=Mdh1 Malate dehydrogenase, cytoplasmic	105	0.84
**IPI00212523.1**	Tax=10116 GS=Park7 Protein DJ-1	21	0.86
**IPI00214052.1**	Tax=10116 GS=Cmbl Carboxymethylenebutenolidase homolog	13	0.87
**IPI00454264.1**	Tax=10116 GS=- LRRGT00046	16	0.89
**IPI00231506.5**	Tax=10116 GS=Pgam2 Phosphoglycerate mutase 2	26	0.91
**IPI00230942.5**	Tax=10116 GS=Gstm3 Glutathione S-transferase Yb-3	6	0.94
**IPI00230937.5**	Tax=10116 GS=Pebp1 Phosphatidylethanolamine-binding protein 1	55	0.95
**IPI00411230.3**	Tax=10116 GS=Gstm2 Glutathione S-transferase Mu 2	5	0.95
**IPI00231767.5**	Tax=10116 GS=Tpi1 Triosephosphate isomerase	59	0.99
**IPI00210351.2**	Tax=10116 GS=Ak1 Adenylate kinase isoenzyme 1	47	1.00
**IPI00231194.5**	Tax=10116 GS=Ddah1 N(G),N(G)-dimethylarginine dimethylaminohydrolase 1	3	1.04
**IPI00763589.2**	Tax=10116 GS=LOC684681 histone cluster 1, H1c-like	5	1.06
**IPI00949396.1**	Tax=10116 GS=- Uncharacterized protein	7	1.06
**IPI00208636.3**	Tax=10116 GS=Gstm5 Glutathione S-transferase Mu 5	2	1.07
**IPI00198497.1**	Tax=10116 GS=Csrp3 Cysteine and glycine-rich protein 3	20	1.10
**IPI00951939.1**	Tax=10116 GS=Srsf2 Uncharacterized protein	3	1.15
**IPI00231971.5**	Tax=10116 GS=Fabp3 Fatty acid-binding protein, heart	805	1.30
**IPI00214517.3**	Tax=10116 GS=Mb Myoglobin	352	1.34

Table S1-common

IPI NO.	DESCRIPTION	Gene Name	Biological Process	(Sub)Cellular Location	Log2_Ratio(avg.Sham/avg.Control)_E1	Log2_Ratio(avg.Sham/avg.Control)_E2	Ratio(avg.Sham/avg.Control)_E1	Ratio(avg.Sham/avg.Control)_E2
**IPI00189981.1**	Tax_Id=10116 Gene_Symbol=F2 Prothrombin (Fragment)	F2	response to stimulus	Blood	-1.19	-2.17	0.44	0.22
**IPI00191737.6**	Tax_Id=10116 Gene_Symbol=Alb Serum albumin	Alb	biological regulation	Blood	-1.69	-1.72	0.31	0.30
**IPI00194097.5**	Tax_Id=10116 Gene_Symbol=Gc Vitamin D-binding protein	Gc	localization	Blood	-1.21	-1.62	0.43	0.32
**IPI00195516.6**	Tax_Id=10116 Gene_Symbol=Hpx Hemopexin	Hpx	metabolic process	Blood	-1.22	-1.11	0.43	0.46
**IPI00197703.2**	Tax_Id=10116 Gene_Symbol=Apoa1 Apolipoprotein A-I	Apoa1	biological regulation	Blood	-1.47	-1.71	0.36	0.30
**IPI00197711.1**	Tax_Id=10116 Gene_Symbol=Ldha L-lactate dehydrogenase A chain	Ldha	metabolic process	Cell	0.74	0.72	1.67	1.64
**IPI00198497.1**	Tax_Id=10116 Gene_Symbol=Csrp3 Cysteine and glycine-rich protein 3	Csrp3	biological regulation	Cell	0.77	1.10	1.71	2.14
**IPI00198667.7**	Tax_Id=10116 Gene_Symbol=Clu Clusterin	Clu	biological regulation	Blood	-1.27	-1.50	0.41	0.35
**IPI00198717.8**	Tax_Id=10116 Gene_Symbol=Mdh1 Malate dehydrogenase, cytoplasmic	Mdh1	metabolic process	Cell	1.03	0.84	2.04	1.79
**IPI00200591.1**	Tax_Id=10116 Gene_Symbol=LOC299282 Serine protease inhibitor A3L	Serpina3l	biological regulation	Blood	-1.28	-1.55	0.41	0.34
**IPI00200593.1**	Tax_Id=10116 Gene_Symbol=Serpina3k Serine protease inhibitor A3K	Serpina3k	biological regulation	Blood	-1.46	-1.51	0.36	0.35
**IPI00201262.1**	Tax_Id=10116 Gene_Symbol=Mug1;LOC297568 Alpha-1-inhibitor 3	Mug1	response to stimulus	Blood	-1.70	-1.62	0.31	0.33
**IPI00206780.1**	Tax_Id=10116 Gene_Symbol=Plg Plasminogen	Plg	metabolic process	Blood	-2.15	-2.15	0.23	0.23
**IPI00210120.2**	Tax_Id=10116 Gene_Symbol=Vtn Uncharacterized protein	Vtn	biological regulation	Blood	-2.06	-2.51	0.24	0.18
**IPI00210351.2**	Tax_Id=10116 Gene_Symbol=Ak1 Adenylate kinase isoenzyme 1	Ak1	metabolic process	Cell	1.09	1.00	2.13	2.00
**IPI00210900.1**	Tax_Id=10116 Gene_Symbol=Ambp Protein AMBP	Ambp	metabolic process	Blood	-0.78	-0.90	0.58	0.53
**IPI00211053.6**	Tax_Id=10116 Gene_Symbol=Ckm Creatine kinase M-type	Ckm	metabolic process	Cell	0.80	0.70	1.74	1.62
**IPI00212523.1**	Tax_Id=10116 Gene_Symbol=Park7 Protein DJ-1	Park7	response to stimulus	Cell	0.84	0.86	1.78	1.82
**IPI00214517.3**	Tax_Id=10116 Gene_Symbol=Mb Myoglobin	Mb	response to stimulus	Cell	1.64	1.34	3.11	2.52
**IPI00230937.5**	Tax_Id=10116 Gene_Symbol=Pebp1 Phosphatidylethanolamine-binding protein 1	Pebp1	biological regulation	Cell	1.08	0.95	2.11	1.93
**IPI00230944.2**	Tax_Id=10116 Gene_Symbol=Fgg Isoform Gamma-A of Fibrinogen gamma chain	Fgg	response to stimulus	Blood	-1.50	-1.80	0.35	0.29
**IPI00231260.5**	Tax_Id=10116 Gene_Symbol=Prdx6 Peroxiredoxin-6	Prdx6	metabolic process	Cell	0.96	0.79	1.94	1.73
**IPI00231426.6**	Tax_Id=10116 Gene_Symbol=Pgk1 Phosphoglycerate kinase 1	Pgk1	metabolic process	Cell	0.98	0.73	1.97	1.66
**IPI00231506.5**	Tax_Id=10116 Gene_Symbol=Pgam2 Phosphoglycerate mutase 2	Pgam2	metabolic process	Cell	0.76	0.91	1.69	1.87
**IPI00231737.5**	Tax_Id=10116 Gene_Symbol=Akr1b1 Aldose reductase	Akr1b1	metabolic process	Cell	0.81	0.64	1.75	1.56
**IPI00231767.5**	Tax_Id=10116 Gene_Symbol=Tpi1 Triosephosphate isomerase	Tpi1	metabolic process	Cell	0.90	0.99	1.87	1.99
**IPI00231783.5**	Tax_Id=10116 Gene_Symbol=Ldhb L-lactate dehydrogenase B chain	Ldhb	metabolic process	Cell	0.88	0.72	1.84	1.65
**IPI00231929.7**	Tax_Id=10116 Gene_Symbol=Pkm2 Isoform M1 of Pyruvate kinase isozymes M1/M2	Pkm2	metabolic process	Cell	0.75	0.64	1.69	1.56
**IPI00231971.5**	Tax_Id=10116 Gene_Symbol=Fabp3 Fatty acid-binding protein, heart	Fabp3	metabolic process	Cell	1.49	1.30	2.82	2.46
**IPI00324019.1**	Tax_Id=10116 Gene_Symbol=Serpina1 Alpha-1-antiproteinase	Serpina1	biological regulation	Blood	-1.56	-1.53	0.34	0.35
**IPI00324272.1**	Tax_Id=10116 Gene_Symbol=Apoa4 Apolipoprotein A-IV	Apoa4	biological regulation	Blood	-0.95	-1.53	0.52	0.35
**IPI00325189.4**	Tax_Id=10116 Gene_Symbol=Nme2 Nucleoside diphosphate kinase B	Nme2	metabolic process	Cell	0.98	0.80	1.97	1.74
**IPI00326140.3**	Tax_Id=10116 Gene_Symbol=Pzp Alpha-1-macroglobulin	Pzp	biological regulation	Blood	-1.43	-1.60	0.37	0.33
**IPI00327469.1**	Tax_Id=10116 Gene_Symbol=Ahsg Alpha-2-HS-glycoprotein	Ahsg	response to stimulus	Blood	-1.40	-1.40	0.38	0.38

**IPI00362791.3**	Tax_Id=10116 Gene_Symbol=Ubqln2 Uncharacterized protein	Ubql n2	biological regulation	Cell	-0.61	-0.97	0.66	0.51
**IPI00368608.2**	Tax_Id=10116 Gene_Symbol=IgG-2a IgG-2a protein	IgG-2a	biological regulation	Bloo d	-1.05	-0.71	0.48	0.61
**IPI00368704.3**	Tax_Id=10116 Gene_Symbol=Cpamd8 similar to Murinoglobulin 1 homolog	Cpa md8	response to stimulus	Bloo d	-1.70	-1.69	0.31	0.31
**IPI00382317.1**	Tax_Id=10116 Gene_Symbol=Fga Uncharacterized protein	Fga	response to stimulus	Bloo d	-1.61	-1.62	0.33	0.32
**IPI00421428.9**	Tax_Id=10116 Gene_Symbol=Pgam1 Phosphoglycerate mutase 1	Pga m1	metabolic process	Cell	0.83	0.67	1.77	1.59
**IPI00421513.8**	Tax_Id=10116 Gene_Symbol=Got1 Aspartate aminotransferase, cytoplasmic	Got1	metabolic process	Cell	0.93	0.75	1.91	1.68
**IPI00480639.3**	Tax_Id=10116 Gene_Symbol=C3 Complement C3 (Fragment)	C3	response to stimulus	Bloo d	-1.58	-1.78	0.33	0.29
**IPI00564327.3**	Tax_Id=10116 Gene_Symbol=Mug2 Uncharacterized protein	Mug 2	response to stimulus	Bloo d	-1.68	-1.64	0.31	0.32
**IPI00565708.2**	Tax_Id=10116 Gene_Symbol=Hp Isoform 1 of Haptoglobin	Hp	response to stimulus	Bloo d	-0.83	-1.09	0.56	0.47
**IPI00567916.3**	Tax_Id=10116 Gene_Symbol=Fetub Fetuin-B	Fetu b	biological regulation	Bloo d	-1.62	-1.40	0.33	0.38
**IPI00679202.2**	Tax_Id=10116 Gene_Symbol=Tf Isoform 1 of Serotransferrin	Tf	response to stimulus	Bloo d	-1.36	-1.38	0.39	0.38
**IPI00778633.1**	Tax_Id=10116 Gene_Symbol=Apoh Uncharacterized protein	Apoh	biological regulation	Bloo d	-1.90	-2.24	0.27	0.21
**IPI00895591.1**	Tax_Id=10116 Gene_Symbol=Pygm glycogen phosphorylase, muscle form	Pyg m	metabolic process	Cell	0.78	0.61	1.72	1.52
**IPI00923716.1**	Tax_Id=10116 Gene_Symbol=Gsn Isoform 2 of Gelsolin	Gsn	metabolic process	Cell	-1.65	-1.96	0.32	0.26
**IPI00948869.1**	Tax_Id=10116 Gene_Symbol=Pygb glycogen phosphorylase, brain form	Pygb	metabolic process	Cell	0.75	0.61	1.68	1.53
**IPI00949032.1**	Tax_Id=10116 Gene_Symbol=Itih3 Uncharacterized protein	Itih3	metabolic process	Bloo d	-1.62	-2.02	0.33	0.25
**IPI00188225.1**	Tax_Id=10116 Gene_Symbol=Crp C-reactive protein	Crp	response to stimulus	Bloo d	-1.95	-2.98	0.26	0.13
**IPI00188541.3**	Tax_Id=10116 Gene_Symbol=Itih4 inter-alpha-inhibitor H4 heavy chain	Itih4	metabolic process	Bloo d	-1.74	-1.75	0.30	0.30
**IPI00190701.5**	Tax_Id=10116 Gene_Symbol=Apoe Apolipoprotein E	Apoe	biological regulation	Bloo d	-1.31	-1.68	0.40	0.31
**IPI00194404.5**	Tax_Id=10116 Gene_Symbol=Nme1 Nucleoside diphosphate kinase A	Nme 1	metabolic process	Cell	0.93	0.75	1.91	1.68
**IPI00204451.1**	Tax_Id=10116 Gene_Symbol=Cfi Complement factor I	Cfi	response to stimulus	Bloo d	-1.31	-0.67	0.40	0.63
**IPI00207668.1**	Tax_Id=10116 Gene_Symbol=Afm Afamin	Afm	localization	Bloo d	-1.31	-1.84	0.40	0.28
**IPI00208306.1**	Tax_Id=10116 Gene_Symbol=Tpt1 Translationally-controlled tumor protein	Tpt1	biological regulation	Cell	0.94	0.75	1.91	1.69
**IPI00211075.2**	Tax_Id=10116 Gene_Symbol=Serpina3n Serpina3n-like protein	Serpi na3n	biological regulation	Bloo d	-1.49	-0.92	0.36	0.53
**IPI00214052.1**	Tax_Id=10116 Gene_Symbol=Cmbl Carboxymethylenebutenolidase homolog	Cmbl	metabolic process	Cell	1.02	0.87	2.03	1.83
**IPI00231264.7**	Tax_Id=10116 Gene_Symbol=Pon1 Serum paraoxonase/arylesterase 1	Pon1	metabolic process	Bloo d	-1.11	-1.21	0.46	0.43
**IPI00231423.6**	Tax_Id=10116 Gene_Symbol=C9 Uncharacterized protein	C9	response to stimulus	Bloo d	-1.37	-2.04	0.39	0.24
**IPI00231434.6**	Tax_Id=10116 Gene_Symbol=Fkbp1a Peptidyl-prolyl cis-trans isomerase FKBP1A	Fkbp 1a	metabolic process	Cell	0.64	0.74	1.56	1.67
**IPI00392216.1**	Tax_Id=10116 Gene_Symbol=LOC299282 Liver regeneration protein lrryan	LOC 2992 82	biological regulation	Bloo d	-1.23	-1.51	0.42	0.35
**IPI00411230.3**	Tax_Id=10116 Gene_Symbol=Gstm2 Glutathione S-transferase Mu 2	Gstm 2	metabolic process	Cell	1.15	0.95	2.23	1.93
**IPI00422011.2**	Tax_Id=10116 Gene_Symbol=Cfb complement factor B	Cfb	response to stimulus	Bloo d	-1.49	-1.21	0.36	0.43
**IPI00464791.5**	Tax_Id=10116 Gene_Symbol=Acy1 Aminoacylase-1A	Acy1	metabolic process	Cell	0.60	0.77	1.51	1.70
**IPI00561922.5**	Tax_Id=10116 Gene_Symbol=Hrg Uncharacterized protein	Hrg	biological regulation	Bloo d	-0.81	-1.72	0.57	0.30
**IPI00656375.3**	Tax_Id=10116 Gene_Symbol=RGD1562758 glyceraldehyde-3-phosphate dehydrogenase-like	RGD 1562 758	metabolic process	Cell	0.66	0.60	1.58	1.51
**IPI00679245.2**	Tax_Id=10116 Gene_Symbol=Kng1l1 T-kininogen 2	Kng1 l1	biological regulation	Bloo d	-0.80	-1.26	0.57	0.42
**IPI00777179.1**	Tax_Id=10116 Gene_Symbol=Anxa1 Uncharacterized protein	Anxa 1	biological regulation	Cell	-0.84	-0.80	0.56	0.58
**IPI00778265.1**	Tax_Id=10116 Gene_Symbol=Cfh Uncharacterized protein	Cfh	response to stimulus	Bloo d	-2.29	-1.08	0.20	0.47
**IPI00876581.1**	Tax_Id=10116 Gene_Symbol=Rbp4 Retinol-binding protein 4	Rbp4	biological regulation	Bloo d	-1.84	-1.49	0.28	0.36
**IPI00948338.1**	Tax_Id=10116 Gene_Symbol=C4b complement C4 precursor	C4b	response to stimulus	Bloo d	-0.82	-1.41	0.57	0.38

**IPI00949068.1**	Tax_Id=10116 Gene_Symbol=Pgm1 Uncharacterized protein	Pgm 1	metabolic process	Cell	0.74	0.63	1.67	1.55
**IPI00951813.1**	Tax_Id=10116 Gene_Symbol=Ugp2 Uncharacterized protein (Fragment)	Ugp2	metabolic process	Cell	1.01	0.73	2.02	1.66
**IPI00951939.1**	Tax_Id=10116 Gene_Symbol=Srsf2 Uncharacterized protein	Srsf2	metabolic process	Cell	0.93	1.15	1.90	2.22
**IPI00957456.1**	Tax_Id=10116 Gene_Symbol=LOC100365275 microsomal carboxyesterase E1-like	LOC 1003 6527 5	metabolic process	Cell	-1.68	-1.59	0.31	0.33
**IPI00958555.1**	Tax_Id=10116 Gene_Symbol=- Uncharacterized protein	N/A	N/A	N/A	-1.26	-1.27	0.42	0.41
**IPI00991200.1**	Tax_Id=10116 Gene_Symbol=Ryr2 Islet-type ryanodine receptor	Ryr2	response to stimulus	Cell mem bran e	-0.76	-0.63	0.59	0.65
**IPI01016420.1**	Tax_Id=10116 Gene_Symbol=Serpina3m Serine protease inhibitor A3M (Fragment)	Serpi na3 m	biological regulation	Bloo d	-1.01	-1.24	0.50	0.42

Table S2. Proteins exhibiting significant quantitative differences in the comparison of both the WE and control.

Table S2-WE_control

IPI NO.	DESCRIPTION	Gene Name	Biological Process	(Sub)Cellular Location	Log2_ Ratio(avg.WE/avg.Control)	Ratio(avg.WE/avg.Control)
**IPI00188225.1**	Tax=10116 GS=Crp C-reactive protein	Crp	response to stimulus	Blood	-1.99	0.25
**IPI00188541.3**	Tax=10116 GS=Itih4 inter-alpha-inhibitor H4 heavy chain	Itih4	metabolic process	Blood	-1.44	0.37
**IPI00189981.1**	Tax=10116 GS=F2 Prothrombin (Fragment)	F2	response to stimulus	Blood	-1.91	0.27
**IPI00190559.3**	Tax=10116 GS=Ube2n Ubiquitin-conjugating enzyme E2 N	Ube2n	metabolic process	Cell	0.76	1.69
**IPI00190701.5**	Tax=10116 GS=Apoe Apolipoprotein E	Apoe	biological regulation	Blood	-1.53	0.35
**IPI00191737.6**	Tax=10116 GS=Alb Serum albumin	Alb	biological regulation	Blood	-1.11	0.46
**IPI00193233.1**	Tax=10116 GS=Cyb5b Cytochrome b5 type B	Cyb5b	metabolic process	Cell	0.60	1.51
**IPI00194097.5**	Tax=10116 GS=Gc Vitamin D-binding protein	Gc	localization	Blood	-1.21	0.43
**IPI00194404.5**	Tax=10116 GS=Nme1 Nucleoside diphosphate kinase A	Nme1	metabolic process	Cell	0.64	1.56
**IPI00195067.1**	Tax=10116 GS=Rasgrf2 Ras-specific guanine nucleotide-releasing factor 2	Rasgrf2	biological regulation	Cell	-0.61	0.66
**IPI00195516.6**	Tax=10116 GS=Hpx Hemopexin	Hpx	metabolic process	Blood	-0.92	0.53
**IPI00196750.1**	Tax=10116 GS=Ssbp1 Single-stranded DNA-binding protein, mitochondrial	Ssbp1	metabolic process	Cell	0.60	1.51
**IPI00197703.2**	Tax=10116 GS=Apoa1 Apolipoprotein A-I	Apoa1	biological regulation	Blood	-1.27	0.41
**IPI00197711.1**	Tax=10116 GS=Ldha L-lactate dehydrogenase A chain	Ldha	metabolic process	Cell	0.81	1.75
**IPI00198497.1**	Tax=10116 GS=Csrp3 Cysteine and glycine-rich protein 3	Csrp3	biological regulation	Cell	1.04	2.06
**IPI00198667.7**	Tax=10116 GS=Clu Clusterin	Clu	biological regulation	Blood	-1.47	0.36
**IPI00198717.8**	Tax=10116 GS=Mdh1 Malate dehydrogenase, cytoplasmic	Mdh1	metabolic process	Cell	0.94	1.91
**IPI00200352.1**	Tax=10116 GS=Crip2 Cysteine-rich protein 2	Crip2	biological regulation	Cell	1.07	2.09
**IPI00200591.1**	Tax=10116 GS=LOC299282 Serine protease inhibitor A3L	Serpina3l	biological regulation	Blood	-1.23	0.43
**IPI00200593.1**	Tax=10116 GS=Serpina3k Serine protease inhibitor A3K	Serpina3k	biological regulation	Blood	-1.13	0.46
**IPI00201262.1**	Tax=10116 GS=Mug1;LOC297568 Alpha-1-inhibitor 3	Mug1	response to stimulus	Blood	-1.19	0.44
**IPI00203972.2**	Tax=10116 GS=Gys1 Glycogen [starch] synthase, muscle	Gys1	metabolic process	Cell	0.61	1.52
**IPI00206399.1**	Tax=10116 GS=Txn2 Thioredoxin, mitochondrial	Txn2	response to stimulus	Cell	0.66	1.58
**IPI00206780.1**	Tax=10116 GS=Plg Plasminogen	Plg	metabolic process	Blood	-1.82	0.28
**IPI00207668.1**	Tax=10116 GS=Afm Afamin	Afm	localization	Blood	-1.09	0.47
**IPI00208306.1**	Tax=10116 GS=Tpt1 Translationally-controlled tumor protein	Tpt1	biological regulation	Cell	0.62	1.54
**IPI00208636.3**	Tax=10116 GS=Gstm5 Glutathione S-transferase Mu 5	Gstm5	metabolic process	Cell	0.84	1.79
**IPI00210120.2**	Tax=10116 GS=Vtn Uncharacterized protein	Vtn	biological regulation	Blood	-2.21	0.22
**IPI00210351.2**	Tax=10116 GS=Ak1 Adenylate kinase isoenzyme 1	Ak1	metabolic process	Cell	0.79	1.73
**IPI00210900.1**	Tax=10116 GS=Ambp Protein AMBP	Ambp	metabolic process	Blood	-0.71	0.61
**IPI00211053.6**	Tax=10116 GS=Ckm Creatine kinase M-type	Ckm	metabolic process	Cell	0.61	1.53
**IPI00211075.2**	Tax=10116 GS=Serpina3n Serpina3n-like protein	Serpina3n	biological regulation	Blood	-0.87	0.55
**IPI00212523.1**	Tax=10116 GS=Park7 Protein DJ-1	Park7	response to stimulus	Cell	0.69	1.61

**IPI00213659.3**	Tax=10116 GS=Decr1 2,4-dienoyl-CoA reductase, mitochondrial	Decr1	metabolic process	Cell	-0.60	0.66
**IPI00214052.1**	Tax=10116 GS=Cmbl Carboxymethylenebutenolidase homolog	Cmbl	metabolic process	Cell	0.73	1.65
**IPI00214517.3**	Tax=10116 GS=Mb Myoglobin	Mb	response to stimulus	Cell	0.86	1.81
**IPI00230937.5**	Tax=10116 GS=Pebp1 Phosphatidylethanolamine-binding protein 1	Pebp1	biological regulation	Cell	0.66	1.58
**IPI00230942.5**	Tax=10116 GS=Gstm3 Glutathione S-transferase Yb-3	Gstm3	metabolic process	Cell	0.69	1.62
**IPI00230944.2**	Tax=10116 GS=Fgg Isoform Gamma-A of Fibrinogen gamma chain	Fgg	response to stimulus	Blood	-1.76	0.30
**IPI00231194.5**	Tax=10116 GS=Ddah1 N(G),N(G)-dimethylarginine dimethylaminohydrolase 1	Ddah1	biological regulation	Cell	0.85	1.81
**IPI00231215.1**	Tax=10116 GS=Pacsin2 Isoform 2 of Protein kinase C and casein kinase substrate in neurons 2 protein	Pacsin 2	biological regulation	Cell	0.70	1.63
**IPI00231264.7**	Tax=10116 GS=Pon1 Serum paraoxonase/arylesterase 1	Pon1	metabolic process	Blood	-0.91	0.53
**IPI00231275.7**	Tax=10116 GS=Lgals1 Galectin-1	Lgals1	biological regulation	Cell	0.61	1.53
**IPI00231423.6**	Tax=10116 GS=C9 Uncharacterized protein	C9	response to stimulus	Blood	-1.88	0.27
**IPI00231426.6**	Tax=10116 GS=Pgk1 Phosphoglycerate kinase 1	Pgk1	metabolic process	Cell	0.63	1.55
**IPI00231434.6**	Tax=10116 GS=Fkbp1a Peptidyl-prolyl cis-trans isomerase FKBP1A	Fkbp1a	metabolic process	Cell	0.73	1.66
**IPI00231506.5**	Tax=10116 GS=Pgam2 Phosphoglycerate mutase 2	Pgam2	metabolic process	Cell	0.85	1.80
**IPI00231767.5**	Tax=10116 GS=Tpi1 Triosephosphate isomerase	Tpi1	metabolic process	Cell	0.75	1.68
**IPI00231783.5**	Tax=10116 GS=Ldhb L-lactate dehydrogenase B chain	Ldhb	metabolic process	Cell	0.81	1.76
**IPI00231929.7**	Tax=10116 GS=Pkm2 Isoform M1 of Pyruvate kinase isozymes M1/M2	Pkm2	metabolic process	Cell	0.64	1.56
**IPI00231971.5**	Tax=10116 GS=Fabp3 Fatty acid-binding protein, heart	Fabp3	metabolic process	Cell	0.82	1.77
**IPI00324019.1**	Tax=10116 GS=Serpina1 Alpha-1-antiproteinase	Serpin a1	biological regulation	Blood	-1.22	0.43
**IPI00324272.1**	Tax=10116 GS=Apoa4 Apolipoprotein A-IV	Apoa4	biological regulation	Blood	-1.04	0.49
**IPI00325189.4**	Tax=10116 GS=Nme2 Nucleoside diphosphate kinase B	Nme2	metabolic process	Cell	0.68	1.60
**IPI00326140.3**	Tax=10116 GS=Pzp Alpha-1-macroglobulin	Pzp	biological regulation	Blood	-1.13	0.46
**IPI00327469.1**	Tax=10116 GS=Ahsg Alpha-2-HS-glycoprotein	Ahsg	response to stimulus	Blood	-0.96	0.51
**IPI00339124.3**	Tax=10116 GS=Atp1b1 Sodium/potassium-transporting ATPase subunit beta-1	Atp1b1	response to stimulus	Cell	0.71	1.64
**IPI00359098.3**	Tax=10116 GS=- Uncharacterized protein	N/A	N/A	N/A	-1.61	0.33
**IPI00361815.3**	Tax=10116 GS=- Uncharacterized protein	N/A	N/A	N/A	0.61	1.52
**IPI00362791.3**	Tax=10116 GS=Ubqln2 Uncharacterized protein	Ubqln2	biological regulation	Cell	-0.63	0.65
**IPI00364189.2**	Tax=10116 GS=Eif3j Eukaryotic translation initiation factor 3 subunit J	Eif3j	metabolic process	Cell	0.65	1.57
**IPI00368704.3**	Tax=10116 GS=Cpamd8 similar to Murinoglobulin 1 homolog	Cpamd 8	response to stimulus	Blood	-1.18	0.44
**IPI00371663.4**	Tax=10116 GS=- Glyceraldehyde-3-phosphate dehydrogenase (Fragment)	Gapdh(fragme nt)	metabolic process	Cell	0.62	1.53
**IPI00372372.2**	Tax=10116 GS=Serpinc1 Uncharacterized protein	Serpinc 1	biological regulation	Blood	-1.03	0.49
**IPI00382312.3**	Tax=10116 GS=Uqcrq Cytochrome b-c1 complex subunit 8	Uqcrq	metabolic process	Cell	0.64	1.56
**IPI00382317.1**	Tax=10116 GS=Fga Uncharacterized protein	Fga	response to stimulus	Blood	-1.59	0.33
**IPI00391297.1**	Tax=10116 GS=Osbpl6 Oxysterol-binding protein	Osbpl6	localization	Cell	-0.74	0.60
**IPI00392216.1**	Tax=10116 GS=LOC299282 Liver regeneration protein lrryan	LOC29 9282	biological regulation	Blood	-1.20	0.44
**IPI00411230.3**	Tax=10116 GS=Gstm2 Glutathione S-transferase Mu 2	Gstm2	metabolic process	Cell	0.70	1.63
**IPI00421428.9**	Tax=10116 GS=Pgam1 Phosphoglycerate mutase 1	Pgam1	metabolic process	Cell	0.69	1.61
**IPI00421513.8**	Tax=10116 GS=Got1 Aspartate aminotransferase, cytoplasmic	Got1	metabolic process	Cell	0.77	1.70
**IPI00422011.2**	Tax=10116 GS=Cfb complement factor B	Cfb	response to stimulus	Blood	-0.82	0.57
**IPI00454264.1**	Tax=10116 GS=- LRRGT00046 (Acyl-CoA-binding protein)	Dbi	metabolic process	Cell	0.82	1.76

**IPI00464791.5**	Tax=10116 GS=Acy1 Aminoacylase-1A	Acy1	metabolic process	Cell	0.84	1.79
**IPI00471687.1**	Tax=10116 GS=Serbp1 Isoform 1 of Plasminogen activator inhibitor 1 RNA-binding protein	Serbp1	biological regulation	Cell	0.71	1.64
**IPI00476292.4**	Tax=10116 GS=Cp Ceruloplasmin	Cp	metabolic process	Blood	-1.05	0.48
**IPI00480639.3**	Tax=10116 GS=C3 Complement C3 (Fragment)	C3	response to stimulus	Blood	-1.36	0.39
**IPI00559057.2**	Tax=10116 GS=- Uncharacterized protein (Fragment)	N/A	N/A	N/A	0.61	1.52
**IPI00559898.2**	Tax=10116 GS=RGD1564688 Glyceraldehyde-3-phosphate dehydrogenase	RGD1564688	metabolic process	Cell	0.63	1.54
**IPI00561262.3**	Tax=10116 GS=- Uncharacterized protein	N/A	N/A	N/A	0.62	1.53
**IPI00561922.5**	Tax=10116 GS=Hrg Uncharacterized protein	Hrg	biological regulation	Blood	-0.88	0.54
**IPI00564327.3**	Tax=10116 GS=Mug2 Uncharacterized protein	Mug2	response to stimulus	Blood	-1.22	0.43
**IPI00565708.2**	Tax=10116 GS=Hp Isoform 1 of Haptoglobin	Hp	response to stimulus	Blood	-0.74	0.60
**IPI00567916.3**	Tax=10116 GS=Fetub Fetuin-B	Fetub	biological regulation	Blood	-1.05	0.48
**IPI00568389.2**	Tax=10116 GS=- LOC500183 protein	LOC500183	N/A	N/A	-0.66	0.63
**IPI00568931.1**	Tax=10116 GS=- Glyceraldehyde-3-phosphate dehydrogenase (Fragment)	Gapdh(fragment)	metabolic process	Cell	0.61	1.53
**IPI00655254.3**	Tax=10116 GS=RGD1310507 Uncharacterized protein	RGD1310507	biological regulation	Blood	-0.89	0.54
**IPI00656375.3**	Tax=10116 GS=RGD1562758 glyceraldehyde-3-phosphate dehydrogenase-like	RGD1562758	metabolic process	Cell	0.63	1.55
**IPI00679202.2**	Tax=10116 GS=Tf Isoform 1 of Serotransferrin	Tf	response to stimulus	Blood	-0.87	0.55
**IPI00679245.2**	Tax=10116 GS=Kng1l1 T-kininogen 2	Kng1l1	biological regulation	Blood	-0.76	0.59
**IPI00763589.2**	Tax=10116 GS=LOC684681 histone cluster 1, H1c-like	LOC684681	localization	Cell	0.98	1.97
**IPI00777179.1**	Tax=10116 GS=Anxa1 Uncharacterized protein	Anxa1	biological regulation	Cell	-0.92	0.53
**IPI00778265.1**	Tax=10116 GS=Cfh Uncharacterized protein	Cfh	response to stimulus	Blood	-0.94	0.52
**IPI00778633.1**	Tax=10116 GS=Apoh Uncharacterized protein	Apoh	biological regulation	Blood	-1.69	0.31
**IPI00780167.2**	Tax=10116 GS=Mug1;LOC297568 Uncharacterized protein	Mug1	response to stimulus	Blood	-1.22	0.43
**IPI00876581.1**	Tax=10116 GS=Rbp4 Retinol-binding protein 4	Rbp4	biological regulation	Blood	-1.05	0.48
**IPI00886485.9**	Tax=10116 GS=Kng1 Rat T-kininogen	Kng1	biological regulation	Blood	-0.68	0.62
**IPI00895591.1**	Tax=10116 GS=Pygm glycogen phosphorylase, muscle form	Pygm	metabolic process	Cell	0.61	1.53
**IPI00900498.1**	Tax=10116 GS=Ptgr2 Isoform 1 of Prostaglandin reductase 2	Ptgr2	metabolic process	Cell	0.62	1.54
**IPI00923716.1**	Tax=10116 GS=Gsn Isoform 2 of Gelsolin	Gsn	metabolic process	Cell	-1.55	0.34
**IPI00948338.1**	Tax=10116 GS=C4b complement C4 precursor	C4b	response to stimulus	Blood	-1.27	0.41
**IPI00948380.2**	Tax=10116 GS=LOC100363606 mCG147639-like (Ig gamma-2B chain C region)	Igh-1a	N/A	Blood	-1.04	0.49
**IPI00948614.1**	Tax=10116 GS=Fgb Uncharacterized protein	Fgb	response to stimulus	Blood	-1.45	0.37
**IPI00948792.1**	Tax=10116 GS=Maoa Uncharacterized protein	Maoa	metabolic process	Cell	-0.61	0.65
**IPI00949032.1**	Tax=10116 GS=Itih3 Uncharacterized protein	Itih3	metabolic process	Blood	-1.39	0.38
**IPI00949396.1**	Tax=10116 GS=- Uncharacterized protein	N/A	N/A	N/A	1.03	2.04
**IPI00957456.1**	Tax=10116 GS=LOC100365275 microsomal carboxyesterase E1-like	LOC100365275	metabolic process	Cell	-1.33	0.40
**IPI00957985.1**	Tax=10116 GS=Fn1 Uncharacterized protein	Fn1	localization	Blood	-1.38	0.38
**IPI00958011.1**	Tax=10116 GS=LOC100365148;LOC100359883 rCG32723-like	LOC100365148	N/A	N/A	-0.82	0.57
**IPI00958555.1**	Tax=10116 GS=- Uncharacterized protein	N/A	N/A	N/A	-0.85	0.56
**IPI00991200.1**	Tax=10116 GS=Ryr2 Islet-type ryanodine receptor	Ryr2	response to stimulus	Cell membrane	-0.72	0.61
**IPI01016420.1**	Tax=10116 GS=Serpina3m Serine protease inhibitor A3M (Fragment)	Serpina3m	biological regulation	Blood	-1.00	0.50

Table S2-all_common

IPI NO.	DESCRIPTION	Log2_ Ratio(avg.Sham/avg. Control)_E1	Log2_ Ratio(avg.Sham/avg. Control)_E2	Log2_ Ratio(avg.WE/avg. Control)
**IPI00189981.1**	Tax_Id=10116 Gene_Symbol=F2 Prothrombin (Fragment)	-1.19	-2.17	-1.91
**IPI00191737.6**	Tax_Id=10116 Gene_Symbol=Alb Serum albumin	-1.69	-1.72	-1.11
**IPI00194097.5**	Tax_Id=10116 Gene_Symbol=Gc Vitamin D-binding protein	-1.21	-1.62	-1.21
**IPI00195516.6**	Tax_Id=10116 Gene_Symbol=Hpx Hemopexin	-1.22	-1.11	-0.92
**IPI00197703.2**	Tax_Id=10116 Gene_Symbol=Apoa1 Apolipoprotein A-I	-1.47	-1.71	-1.27
**IPI00197711.1**	Tax_Id=10116 Gene_Symbol=Ldha L-lactate dehydrogenase A chain	0.74	0.72	0.81
**IPI00198497.1**	Tax_Id=10116 Gene_Symbol=Csrp3 Cysteine and glycine-rich protein 3	0.77	1.10	1.04
**IPI00198667.7**	Tax_Id=10116 Gene_Symbol=Clu Clusterin	-1.27	-1.50	-1.47
**IPI00198717.8**	Tax_Id=10116 Gene_Symbol=Mdh1 Malate dehydrogenase, cytoplasmic	1.03	0.84	0.94
**IPI00200591.1**	Tax_Id=10116 Gene_Symbol=LOC299282 Serine protease inhibitor A3L	-1.28	-1.55	-1.23
**IPI00200593.1**	Tax_Id=10116 Gene_Symbol=Serpina3k Serine protease inhibitor A3K	-1.46	-1.51	-1.13
**IPI00201262.1**	Tax_Id=10116 Gene_Symbol=Mug1;LOC297568 Alpha-1-inhibitor 3	-1.70	-1.62	-1.19
**IPI00206780.1**	Tax_Id=10116 Gene_Symbol=Plg Plasminogen	-2.15	-2.15	-1.82
**IPI00210120.2**	Tax_Id=10116 Gene_Symbol=Vtn Uncharacterized protein	-2.06	-2.51	-2.21
**IPI00210351.2**	Tax_Id=10116 Gene_Symbol=Ak1 Adenylate kinase isoenzyme 1	1.09	1.00	0.79
**IPI00210900.1**	Tax_Id=10116 Gene_Symbol=Ambp Protein AMBP	-0.78	-0.90	-0.71
**IPI00211053.6**	Tax_Id=10116 Gene_Symbol=Ckm Creatine kinase M-type	0.80	0.70	0.61
**IPI00212523.1**	Tax_Id=10116 Gene_Symbol=Park7 Protein DJ-1	0.84	0.86	0.69
**IPI00214517.3**	Tax_Id=10116 Gene_Symbol=Mb Myoglobin	1.64	1.34	0.86
**IPI00230937.5**	Tax_Id=10116 Gene_Symbol=Pebp1 Phosphatidylethanolamine-binding protein 1	1.08	0.95	0.66
**IPI00230944.2**	Tax_Id=10116 Gene_Symbol=Fgg Isoform Gamma-A of Fibrinogen gamma chain	-1.50	-1.80	-1.76
**IPI00231260.5**	Tax_Id=10116 Gene_Symbol=Prdx6 Peroxiredoxin-6	0.96	0.79	0.59
**IPI00231426.6**	Tax_Id=10116 Gene_Symbol=Pgk1 Phosphoglycerate kinase 1	0.98	0.73	0.63
**IPI00231506.5**	Tax_Id=10116 Gene_Symbol=Pgam2 Phosphoglycerate mutase 2	0.76	0.91	0.85
**IPI00231767.5**	Tax_Id=10116 Gene_Symbol=Tpi1 Triosephosphate isomerase	0.90	0.99	0.75
**IPI00231783.5**	Tax_Id=10116 Gene_Symbol=Ldhb L-lactate dehydrogenase B chain	0.88	0.72	0.81
**IPI00231929.7**	Tax_Id=10116 Gene_Symbol=Pkm2 Isoform M1 of Pyruvate kinase isozymes M1/M2	0.75	0.64	0.64
**IPI00231971.5**	Tax_Id=10116 Gene_Symbol=Fabp3 Fatty acid-binding protein, heart	1.49	1.30	0.82
**IPI00324019.1**	Tax_Id=10116 Gene_Symbol=Serpina1 Alpha-1-antiproteinase	-1.56	-1.53	-1.22
**IPI00324272.1**	Tax_Id=10116 Gene_Symbol=Apoa4 Apolipoprotein A-IV	-0.95	-1.53	-1.04
**IPI00325189.4**	Tax_Id=10116 Gene_Symbol=Nme2 Nucleoside diphosphate kinase B	0.98	0.80	0.68
**IPI00326140.3**	Tax_Id=10116 Gene_Symbol=Pzp Alpha-1-macroglobulin	-1.43	-1.60	-1.13
**IPI00327469.1**	Tax_Id=10116 Gene_Symbol=Ahsg Alpha-2-HS-glycoprotein	-1.40	-1.40	-0.96
**IPI00362791.3**	Tax_Id=10116 Gene_Symbol=Ubqln2 Uncharacterized protein	-0.61	-0.97	-0.63
**IPI00368704.3**	Tax_Id=10116 Gene_Symbol=Cpamd8 similar to Murinoglobulin 1 homolog	-1.70	-1.69	-1.18
**IPI00382317.1**	Tax_Id=10116 Gene_Symbol=Fga Uncharacterized protein	-1.61	-1.62	-1.59
**IPI00421428.9**	Tax_Id=10116 Gene_Symbol=Pgam1 Phosphoglycerate mutase 1	0.83	0.67	0.69
**IPI00421513.8**	Tax_Id=10116 Gene_Symbol=Got1 Aspartate aminotransferase, cytoplasmic	0.93	0.75	0.77
**IPI00480639.3**	Tax_Id=10116 Gene_Symbol=C3 Complement C3 (Fragment)	-1.58	-1.78	-1.36
**IPI00564327.3**	Tax_Id=10116 Gene_Symbol=Mug2 Uncharacterized protein	-1.68	-1.64	-1.22
**IPI00565708.2**	Tax_Id=10116 Gene_Symbol=Hp Isoform 1 of Haptoglobin	-0.83	-1.09	-0.74
**IPI00567916.3**	Tax_Id=10116 Gene_Symbol=Fetub Fetuin-B	-1.62	-1.40	-1.05
**IPI00679202.2**	Tax_Id=10116 Gene_Symbol=Tf Isoform 1 of Serotransferrin	-1.36	-1.38	-0.87

**IPI00778633.1**	Tax_Id=10116 Gene_Symbol=Apoh Uncharacterized protein	-1.90	-2.24	-1.69
**IPI00895591.1**	Tax_Id=10116 Gene_Symbol=Pygm glycogen phosphorylase, muscle form	0.78	0.61	0.61
**IPI00923716.1**	Tax_Id=10116 Gene_Symbol=Gsn Isoform 2 of Gelsolin	-1.65	-1.96	-1.55
**IPI00949032.1**	Tax_Id=10116 Gene_Symbol=Itih3 Uncharacterized protein	-1.62	-2.02	-1.39
**IPI00188225.1**	Tax_Id=10116 Gene_Symbol=Crp C-reactive protein	-1.95	-2.98	-1.99
**IPI00188541.3**	Tax_Id=10116 Gene_Symbol=Itih4 inter-alpha-inhibitor H4 heavy chain	-1.74	-1.75	-1.44
**IPI00190701.5**	Tax_Id=10116 Gene_Symbol=Apoe Apolipoprotein E	-1.31	-1.68	-1.53
**IPI00194404.5**	Tax_Id=10116 Gene_Symbol=Nme1 Nucleoside diphosphate kinase A	0.93	0.75	0.64
**IPI00207668.1**	Tax_Id=10116 Gene_Symbol=Afm Afamin	-1.31	-1.84	-1.09
**IPI00208306.1**	Tax_Id=10116 Gene_Symbol=Tpt1 Translationally-controlled tumor protein	0.94	0.75	0.62
**IPI00211075.2**	Tax_Id=10116 Gene_Symbol=Serpina3n Serpina3n-like protein	-1.49	-0.92	-0.87
**IPI00214052.1**	Tax_Id=10116 Gene_Symbol=Cmbl Carboxymethylenebutenolidase homolog	1.02	0.87	0.73
**IPI00231264.7**	Tax_Id=10116 Gene_Symbol=Pon1 Serum paraoxonase/arylesterase 1	-1.11	-1.21	-0.91
**IPI00231423.6**	Tax_Id=10116 Gene_Symbol=C9 Uncharacterized protein	-1.37	-2.04	-1.88
**IPI00231434.6**	Tax_Id=10116 Gene_Symbol=Fkbp1a Peptidyl-prolyl cis-trans isomerase FKBP1A	0.64	0.74	0.73
**IPI00392216.1**	Tax_Id=10116 Gene_Symbol=LOC299282 Liver regeneration protein lrryan	-1.23	-1.51	-1.20
**IPI00411230.3**	Tax_Id=10116 Gene_Symbol=Gstm2 Glutathione S-transferase Mu 2	1.15	0.95	0.70
**IPI00422011.2**	Tax_Id=10116 Gene_Symbol=Cfb complement factor B	-1.49	-1.21	-0.82
**IPI00464791.5**	Tax_Id=10116 Gene_Symbol=Acy1 Aminoacylase-1A	0.60	0.77	0.84
**IPI00561922.5**	Tax_Id=10116 Gene_Symbol=Hrg Uncharacterized protein	-0.81	-1.72	-0.88
**IPI00656375.3**	Tax_Id=10116 Gene_Symbol=RGD1562758 glyceraldehyde-3-phosphate dehydrogenase-like	0.66	0.60	0.63
**IPI00679245.2**	Tax_Id=10116 Gene_Symbol=Kng1l1 T-kininogen 2	-0.80	-1.26	-0.76
**IPI00777179.1**	Tax_Id=10116 Gene_Symbol=Anxa1 Uncharacterized protein	-0.84	-0.80	-0.92
**IPI00778265.1**	Tax_Id=10116 Gene_Symbol=Cfh Uncharacterized protein	-2.29	-1.08	-0.94
**IPI00876581.1**	Tax_Id=10116 Gene_Symbol=Rbp4 Retinol-binding protein 4	-1.84	-1.49	-1.05
**IPI00948338.1**	Tax_Id=10116 Gene_Symbol=C4b complement C4 precursor	-0.82	-1.41	-1.27
**IPI00949068.1**	Tax_Id=10116 Gene_Symbol=Pgm1 Uncharacterized protein	0.74	0.63	0.59
**IPI00957456.1**	Tax_Id=10116 Gene_Symbol=LOC100365275 microsomal carboxyesterase E1-like	-1.68	-1.59	-1.33
**IPI00958555.1**	Tax_Id=10116 Gene_Symbol=- Uncharacterized protein	-1.26	-1.27	-0.85
**IPI00991200.1**	Tax_Id=10116 Gene_Symbol=Ryr2 Islet-type ryanodine receptor	-0.76	-0.63	-0.72
**IPI01016420.1**	Tax_Id=10116 Gene_Symbol=Serpina3m Serine protease inhibitor A3M (Fragment)	-1.01	-1.24	-1.00

Table S3. DAVID Gene Ontology enrichment analyses of proteins exhibiting significant quantitative differences in the comparisons of both the WE and control, and both the WE and control.

Table S3-Sham_control

Category	Term	Count	%	PValue	Genes
**GOTERM_BP_1**	GO:0050896~ response to stimulus	40	58.82	3.55E-08	Pgm1, Tf, Plg, Kng1l1, Park7, C9, Vtn, Cpamd8, Apoh, Gsn, Prdx6, Fga, Pkm2, Alb, Akr1b1, Got1, Serpina3n, Gstm2, Mb, Cfh, Clu, Fkbp1a, Mug2, Pgam2, Serpina1, Rbp4, Fabp3, Cfb, Pon1, Nme1, F2, Ldha, Anxa1, Apoe, Pebp1, Fgg, Crp, Pygm, Hp, Ahsg
**GOTERM_BP_1**	GO:0008152~ metabolic process	42	61.76	2.01E-04	Pgk1, Pgm1, Plg, Park7, C9, RGD1562758, Apoh, Acy1, Prdx6, Pkm2, Akr1b1, Got1, Gstm2, Cfh, Ckm, Fkbp1a, Hpx, Pgam2, Ambp, Serpina1, Ldhb, Ugp2, Itih3, Nme2, Tpi1, Rbp4, Fabp3, Cfb, Pon1, F2, Nme1, Ldha, Anxa1, Pygb, Srsf2, Apoe, Crp, Pygm, Hp, Mdh1, Itih4, Gc
**GOTERM_BP_1**	GO:0009987~ cellular process	47	69.12	5.38E-03	Pgk1, Pgm1, Tpt1, Tf, Plg, Park7, RGD1562758, Vtn, Csrp3, C9, Apoh, Acy1, Gsn, Prdx6, Fga, Pkm2, Alb, Akr1b1, Got1, Gstm2, Mb, Ckm, Clu, Fkbp1a, Hpx, Ambp, Pgam2, Serpina1, Ldhb, Ugp2, Nme2, Tpi1, Rbp4, Fabp3, Pon1, Nme1, F2, Ldha, Anxa1, Pygb, Srsf2, Apoe, Pebp1, Fgg, Pygm, Ahsg, Mdh1
**GOTERM_BP_1**	GO:0065007~ biological regulation	36	52.94	1.12E-02	Pgm1, Tpt1, Tf, Plg, Kng1l1, Park7, Csrp3, C9, Vtn, Apoh, Gsn, Prdx6, Fga, Alb, Akr1b1, Got1, Mb, Cfh, Hrg, Clu, Fkbp1a, Hpx, Nme2, Rbp4, Cfb, Pon1, F2, Nme1, Ldha, Anxa1, Apoe, Pebp1, Crp, Fgg, Pygm, Ahsg
**GOTERM_BP_1**	GO:0000003~ reproduction	9	13.24	1.45E-02	Clu, Tpt1, Anxa1, Pgam2, Pebp1, Rbp4, Ahsg, Hp, Nme1
**GOTERM_BP_1**	GO:0051234~ establishment of localization	17	25.00	3.96E-02	Hpx, Fkbp1a, Ambp, Tf, Park7, Fabp3, Apoh, Rbp4, Nme1, Gsn, Anxa1, Apoe, Alb, Crp, Mb, Afm, Gc
**GOTERM_BP_1**	GO:0016265~ death	6	8.82	4.91E-02	Gsn, Clu, Pkm2, Plg, RGD1562758, C9
**GOTERM_BP_1**	GO:0032502~ developmental process	19	27.94	5.60E-02	Clu, Fkbp1a, Plg, Tpi1, Csrp3, Apoh, Rbp4, Nme1, Ldha, Gsn, Fga, Anxa1, Pkm2, Pebp1, Apoe, Gstm2, Hp, Ahsg, Mb
**GOTERM_BP_1**	GO:0040007~ growth	4	5.88	8.27E-02	Gsn, Fga, Pkm2, Plg
**GOTERM_BP_1**	GO:0032501~ multicellular organismal process	27	39.71	9.69E-02	Tpt1, Plg, C9, Park7, Kng1l1, Csrp3, Apoh, Gsn, Fga, Pkm2, Alb, Gstm2, Mb, Clu, Fkbp1a, Pgam2, Tpi1, Rbp4, Ldha, Nme1, F2, Anxa1, Apoe, Pebp1, Fgg, Hp, Ahsg

Table S3-WE_control

Category	Term	Count	%	PValue	Genes
**GOTERM_BP_1**	GO:0050896~ response to stimulus	47	48.42	1.12E-06	Tf, Plg, Kng1l1, Park7, Vtn, C9, Cpamd8, Apoh, Gsn, Fga, Pkm2, Alb, Txn2, Got1, Ube2n, Atp1b1, Serpina3n, Gstm2, Mb, Cfh, Clu, Fkbp1a, Serpinc1, Pgam2, Mug2, Serpina1, Kng1, Rbp4, Fabp3, Cfb, Pon1, Nme1, F2, Ldha, Anxa1, Ddah1, Apoe, Pebp1, Mug1, Fgb, Fgg, Crp, Pygm, Hp, Ahsg, Cp, Gstm3
**GOTERM_BP_1**	GO:0008152~ metabolic process	53	54.74	3.84E-04	Pgk1, Eif3j, C9, N/A(IPI00361815), RGD1562758, Ptgr2, Txn2, Ube2n, Atp1b1, Cfh, Hpx, Ambp, Serpina1, Tpi1, Fabp3, Rbp4, Ldha, Pon1, Anxa1, Crp, Hp, Mdh1, Gstm3, Cyb5b, Plg, RGD1564688, Park7, Apoh, Acy1, Decr1, Ssbp1, Pkm2, Uqcrq, Got1, Gstm2, Ckm, Fkbp1a, Pgam2, Maoa, Ldhb, Itih3, Nme2, Cfb, Gys1, F2, Nme1, Gstm5, Ddah1, Apoe, Pygm, Cp, Gc, Itih4
**GOTERM_BP_1**	GO:0065007~ biological regulation	48	50.53	2.32E-03	Lgals1, Tpt1, Tf, Plg, Crip2, Kng1l1, Park7, Vtn, C9, Csrp3, Rasgrf2, Apoh, Gsn, Fga, Ssbp1, Alb, Txn2, Got1, Ube2n, Mb, Cfh, Hrg, Clu, Serbp1, Fkbp1a, Hpx, Serpinc1, Maoa, Nme2, Kng1, Rbp4, Cfb, Pon1, Ldha, RGD1310507, Nme1, F2, Anxa1, Ddah1, Apoe, Pebp1, Fgb, Pacsin2, Fgg, Crp, Pygm, Ahsg, Cp

**GOTERM_BP_1**	GO:0051234~establishment of localization	24	25.26	5.43E-03	Cyb5b, Hpx, Fkbp1a, Ambp, Tf, Park7, Fabp3, Apoh, Rbp4, Nme1, RGD1310507, Gsn, Anxa1, Apoe, Uqcrq, Alb, Txn2, Pacsin2, Crp, Atp1b1, Cp, Mb, Afm, Gc
**GOTERM_BP_1**	GO:0009987~cellular process	61	63.16	5.83E-03	Pgk1, Tpt1, Eif3j, Vtn, C9, RGD1562758, N/A(IPI00361815), Gsn, Txn2, Ube2n, Atp1b1, Mb, Hpx, Ambp, Serpina1, Tpi1, Fabp3, Rbp4, Ldha, RGD1310507, Pon1, Anxa1, Pebp1, Pacsin2, Fgg, Ahsg, Mdh1, Gstm3, Cyb5b, Lgals1, Tf, Plg, Park7, RGD1564688, Csrp3, Apoh, Acy1, Decr1, Fga, Ssbp1, Pkm2, Uqcrq, Alb, Got1, Gstm2, Ckm, Clu, Fkbp1a, Pgam2, Maoa, Ldhb, Nme2, Kng1, Gys1, F2, Nme1, Ddah1, Apoe, Fgb, Pygm, Cp
**GOTERM_BP_1**	GO:0032502~developmental process	25	26.32	2.78E-02	Lgals1, Clu, Fkbp1a, Plg, Crip2, Tpi1, Csrp3, Apoh, Rbp4, Gys1, Ldha, Nme1, Gsn, Anxa1, Fga, Ssbp1, Ddah1, Pkm2, Pebp1, Apoe, Gstm2, Hp, Mb, Ahsg, Cp
**GOTERM_BP_1**	GO:0051179~localization	24	25.26	3.19E-02	Cyb5b, Hpx, Fkbp1a, Ambp, Tf, Park7, Fabp3, Apoh, Rbp4, Nme1, RGD1310507, Gsn, Anxa1, Apoe, Uqcrq, Alb, Txn2, Pacsin2, Crp, Atp1b1, Cp, Mb, Afm, Gc
**GOTERM_BP_1**	GO:0002376~immune system process	10	10.53	3.51E-02	Ddah1, Fkbp1a, Crip2, Crp, Ube2n, Vtn, C9, Mb, Cfb, Cfh
**GOTERM_BP_1**	GO:0016265~death	8	7.37	4.87E-02	Gsn, Clu, Ddah1, Pkm2, Plg, RGD1562758, N/A(IPI00361815), C9
**GOTERM_BP_1**	GO:0000003~reproduction	9	9.47	6.26E-02	Clu, Tpt1, Anxa1, Pgam2, Pebp1, Rbp4, Ahsg, Hp, Nme1

Table S4. Structures and description of the polysaccharides investigated in this study.

Polysaccharide	Structure	Description
Starch[1)] (amylopectin)	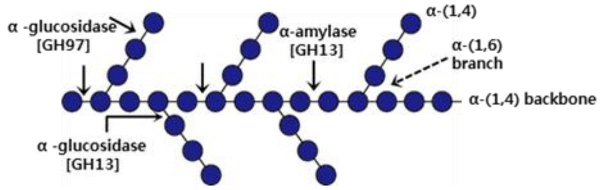	Starch consisting of amylose and amylopectin: amylose, linear α-(1,4)-linked glucose backbone; amylopectin, α-(1,4)-linked glucose backbone with α-(1,6) D-glucose branches.
Arabinoxylan[2)]	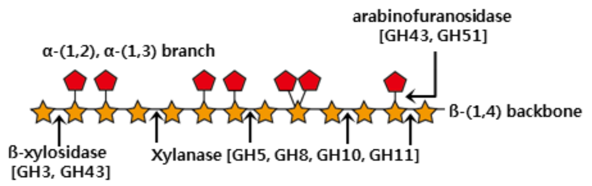	β-(1,4)-linked D-xylose backbone (xylan) substituted with L-arabinose residues. Only 1.8% of the arabinoxylan in wheat grain is water soluble.
Cellulose[2)]	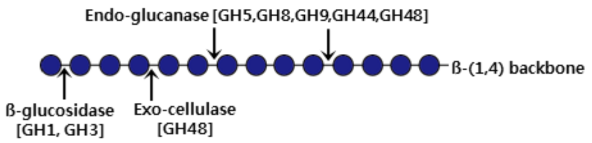	Cellulose that is composed of only a β-(1,4)-linked glucose backbone is insoluble in water, whereas cellulose composed of β-glucan, which is composed of a glucose backbone linked through a mixture of β-(1,4) and β-(1,3) linkages, is water soluble[1].
ß-Glucan[2)]	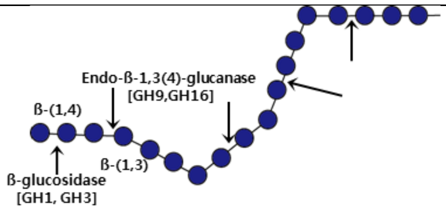	Mixed β-(1,4)- and β-(1,3)-linked glucose backbone. β-glucan is enriched in barley[2].
Arabinogalactan - peptide[2)]	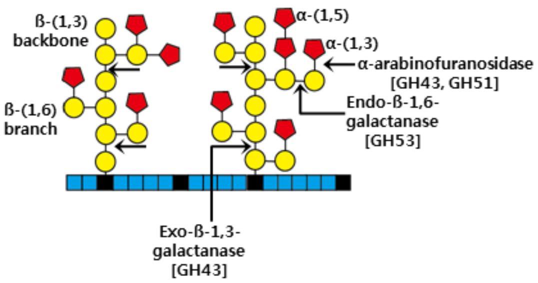	Composed of 90% polysaccharide arabinogalactan and 10% peptides. The arabinogalactan in arabinogalactan-peptide consists of a β-(1,3)-linked D-galactose backbone (galactan) with β-(1,6) D-galactose side chains, which are then substituted with arabinose residues. Arabinogalactan is enriched in lupin[3].
Xylan[2)]	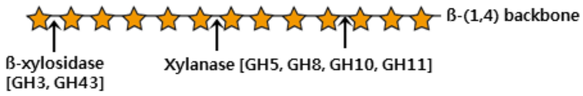	β-(1,4)-linked xylose backbone.

Pectin[2)]	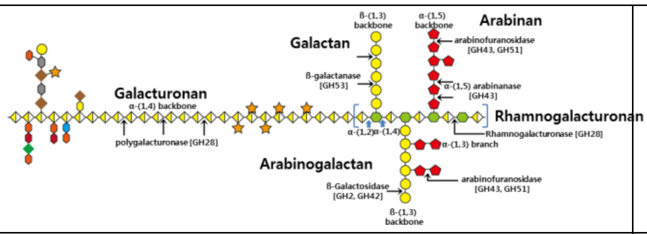	Composed of galacturonan, rhamnogalacturonan, galactan, arabinan and arabinogalactan. In particular, arabinan, a domain of pectin, is enriched in sugar beet[4].
Xyloglucan[2)]	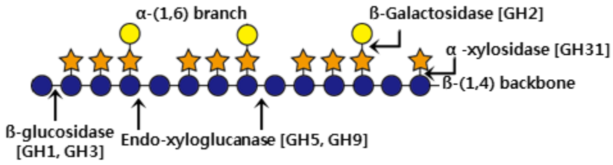	Composed of β-(1,4)-linked glucose backbone (glucan) substituted with xylose residues. Xyloglucan is a major cell wall polysaccharide of dicots[5].
Glucomannan[2)]	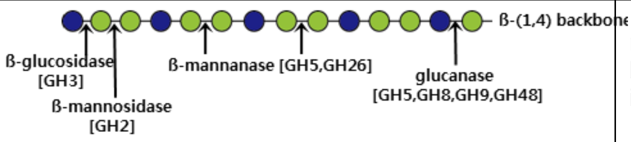	Mixed β-(1,4)-linked mannose and glucose backbone. Glucomannan is enriched in Konjac[6].
Laminarin[3)]	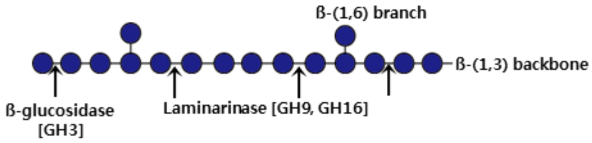	β-(1,3)-linked glucose backbone (glucan) substituted with glucose residues. laminarin is enriched in kelp (*Laminaria*)[7].
Fucoidan[3)]	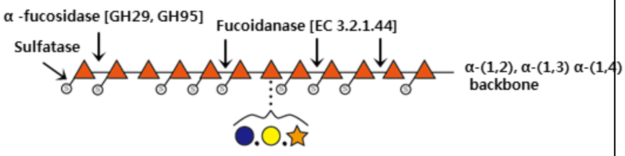	β-(1,3)-linked galactose backbone substituted with glucose, galactose and xylose. Fucoidan is enriched in kelp, sea mustard (*Undaria*) and *Fucus*[7].
Symbols	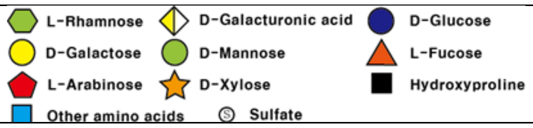	Definitions of symbols used.

For each polysaccharide, the constituent monosaccharides and their linkages are presented. Arrows indicate cleavage sites of the representative enzymes that belong to the corresponding glycoside hydrolase (GH) family numbers. [1)]A starch polysaccharide, amylopectin. [2)]Cell wall polysaccharides of land plants. Of these, pectin is composed of various polysaccharide domains (G). [3)]Cell wall polysaccharides of seaweeds.

References

1. Knudsen, K. E. Fiber and nonstarch polysaccharide content and variation in common crops used in broiler diets. *Poult Sci* **93**, 2380-2393, (2014).
2. Andersson, A. A. *et al.* Phytochemical and dietary fiber components in barley varieties in the HEALTHGRAIN Diversity Screen. *J Agric Food Chem* **56**, 9767-9776, (2008).
3. Buckeridge, M. S. Seed cell wall storage polysaccharides: models to

understand cell wall biosynthesis and degradation. *Plant Physiol* **154**, 1017-1023, (2010).

4. Al-Tamimi, M. A., Palframan, R. J., Cooper, J. M., Gibson, G. R. & Rastall, R. A. In vitro fermentation of sugar beet arabinan and arabino-oligosaccharides by the human gut microflora. *J Appl Microbiol* **100**, 407-414, (2006).
5. Vogel, J. Unique aspects of the grass cell wall. *Curr Opin Plant Biol* **11**, 301-307, (2008).
6. Fang, W. & Wu, P. Variations of Konjac glucomannan (KGM) from Amorphophallus konjac and its refined powder in China. *Food Hydrocolloid* **18**, 167-170, (2004).
7. Holdt, S. L. & Kraan, S. Bioactive compounds in seaweed: functional food applications and legistration. *J Appl Phycol* **23**, 543-597, (2011).

Table S5. Detailed information of antibodies used in this study.

Antibody name	Company	Catalogue code	Dilutions	RRID
anti-Bcl-2 pAb	Santa Cruz Biotechnology	SC-492	1:1000	AB_2064290
anti-Bax pAb	Santa Cruz Biotechnology	SC-493	1:1000	AB_2227995
anti-Phospho-p38 MAPK (Thr180/Tyr182) mAb	Cell Signaling Technology	4511S	1:1000	AB_2139682
anti-p38 MAPK pAb	Cell Signaling Technology	9212	1:1000	AB_330713
anti-Phospho-SAPK/JNK (Thr183/Tyr185) mAb	Cell Signaling Technology	4668S	1:1000	AB_823588
anti-JNK1/3 pAb	Santa Cruz Biotechnology	SC-474	1:1000	AB_632383
anti-Phospho-p44/42 MAPK (Erk1/2) mAb	Cell Signaling Technology	4695S	1:1000	AB_10694379
anti-Myoglobin mAb	Abcam	Ab77232	1:1000	AB_1523998
anti-Gelsolin mAb	Abcam	Ab134183	1:1000	–
anti-Adenylate kinase isoenzyme 1 pAb	Aviva Systems Biology	ARP48148_P050	1:1000	AB_2044888
anti-Fatty acid-binding protein mAb	Abcam	Ab133585	1:1000	–
anti-Carboxymethylenebutenolidase homolog pAb	Proteintech Group	17409-1-AP	1:1000	AB_10596776
anti-Glutathione S-transferase Mu 5 pAb	Aviva Systems Biology	ABIN1078085	1:1000	–
anti-Protein DJ-1 pAb	Cell Signaling Technology	2134S	1:1000	AB_10693043
anti-Nucleoside diphosphate kinase B mAb	Abcam	Ab60602	1:1000	AB_944445
anti-Phosphatidylethanolamine-binding protein 1 pAb	Cell Signaling Technology	5291S	1:1000	AB_1904083
ERK1 pAb	Santa Cruz Biotechnology	SC-93	1:1000	AB_631453
